# Multi-disease transcriptomic analysis of sex hormone genes reveals a novel prognostic model for thyroid cancer with breast cancer correlations

**DOI:** 10.3389/fonc.2025.1641195

**Published:** 2025-09-02

**Authors:** Lixue Qiao, Hao Li, Keyu Yin, Runsheng Ma, Yifei Zhang, Yue Guo, Detao Yin

**Affiliations:** ^1^ Thyroid Surgery Department, The First Affiliated Hospital of Zhengzhou University, Zhengzhou, China; ^2^ Department of Hepatobiliary and Pancreatic Surgery, The First Affiliated Hospital of Zhengzhou University, Zhengzhou, China; ^3^ School of Basic Medical Sciences, Lanzhou University, Lanzhou, Gansu, China; ^4^ Engineering Research Center of Multidisciplinary Diagnosis and Treatment of Thyroid Cancer of Henan Province, Zhengzhou,, China; ^5^ Key Medicine Laboratory of Thyroid Cancer of Henan Province, Zhengzhou, China

**Keywords:** thyroid cancer, breast cancer, sex hormone metabolism-related gene, prognostic model, immune infiltrate

## Abstract

**Background:**

There is a potential bidirectional pathogenicity between thyroid and breast cancers. The association between sex hormones and two types of malignant tumors has emerged as a topic of intense academic debate in recent years. However, the role of sex hormone metabolism-related genes in thyroid cancer still needs to be further explored.

**Methods:**

We obtained thyroid and breast cancer transcriptome data from the TCGA database and sex hormone metabolism-related gene sets from the MSigDB database, thus screening for sex hormone metabolism-related genes linked to the two malignant tumors. Univariate cox regression analysis was used for the screening of disease-free survival (DFS)-associated genes. The TCGA-THCA patients were classified as two categories via a consistent clustering algorithm, and the differential genes between the two categories were subsequently screened. A sex hormone metabolism-related prognostic model (TBSMRPM) of thyroid cancer versus breast cancer consisting of 10 genes was developed by Cox regression analyses and least absolute shrinkage with selection operator (LASSO) cox regression analysis. Finally, we performed clinicopathological subgroup analyses to analyze the correlation between TBSMRPM and clinical characteristics, immune infiltration, tumor mutation burden (TMB), and chemosensitivity, and verified the expression of TBSMRPM signature genes by qRT-PCR.

**Results:**

We identified 2 clusters correlated with sex hormone metabolism, and screened 10 prognostic differential genes related to thyroid cancer, breast cancer and sex hormone metabolism. After establishing the two risk groups for thyroid cancer originated from TBSMRPM, the results showed that the high-risk group exhibited the shorter DFS (*P*<0.05). In further clinical stratification analysis, immune infiltration analysis, TMB and drug sensitivity analysis, the two TBSMRPM groups showed significant differences. The qRT-PCR results showed that *C2CD4A*, *CERS1*, *MMP9*, *SLC5A1*, *HORMAD2* were highly expressed in the IHH4, KTC-1, and TPC-1 cell lines, while *SLITRK2*, *ARHGEF37*, *PLP1*, *RNF223*, and *F3* were lowly expressed.

**Conclusion:**

The TBSMRPM established in this study has a certain value for the prognosis of thyroid cancer and contributes to refine clinicians’ treatment protocols.

## 1 Introduction

Thyroid cancer (THCA) and breast cancer (BRCA), as common hormone-dependent malignancies, their bidirectional pathogenic association and the regulatory role of sex hormones therein have become a focus of oncology research. Epidemiological data clearly demonstrate a significant interaction between these two organ malignancies: Nielsen et al. confirmed that individuals with a history of thyroid cancer have a 1.32-fold higher risk of developing breast cancer compared to the general population, while breast cancer patients have a 1.55-fold increased risk of subsequent thyroid cancer (1). This bidirectional risk suggests that, as hormone-dependent organs, the malignant progression of the thyroid and mammary gland is not an isolated event, and their development is influenced by a complex regulatory network (1–6). Currently, research on their association has involved multiple dimensions, including genetic alterations, hypothalamic-pituitary axis regulation, metabolic abnormalities (e.g., diabetes, obesity), and surveillance bias. Among these, hormone-related mechanisms are particularly critical—various hormone-related factors such as thyroid hormones, sex hormones (with estrogen as the core), endocrine-disrupting chemicals, and adipokines have been confirmed to be involved in the development of both tumors (2). Sex hormones, in particular, have been identified as core regulatory factors due to their direct role in regulating cell proliferation, differentiation, and malignant transformation in thyroid and breast tissues (2). Therefore, systematically elucidating the roles and mechanisms of sex hormone metabolism-related genes associated with both cancers is of great significance for clarifying cross-cancer associations and optimizing clinical diagnosis and treatment.

The metabolic balance of sex hormones is precisely regulated by a network of genes encoding metabolic enzymes and receptors, collectively referred to as sex hormone metabolism-related genes (SMRGs). To date, the roles of SMRGs in breast cancer and thyroid cancer have been partially elucidated. In the field of breast cancer, the clinical value of SMRGs has been verified: for example, aromatase encoded by CYP19A1 is a key rate-limiting enzyme in estrogen synthesis, and its inhibitors have become standard therapeutic agents for ER-positive breast cancer by reducing estrogen production (7); both two-sample Mendelian randomization studies and epidemiological data have shown that elevated levels of total testosterone and bioavailable testosterone increase the risk of ER+ breast cancer, with this association being more pronounced in postmenopausal women (8, 9). In thyroid cancer research, the roles of SMRGs have also been gradually revealed, with the regulatory mechanisms of estrogen and its receptors receiving the most attention: estrogen can enhance the proliferation, migration, and invasion abilities of papillary thyroid carcinoma (PTC) through the ERα/KRT19 signaling axis (10); ERβ is highly expressed in PTC stem cells (PTCSCs), and its knockout can reduce the expression of stemness-related factors, decrease the ALDH+ cell population, and inhibit tumor sphere formation and growth (11); in addition, ERα36, GRP78, and GRP94 are upregulated in primary PTC tissues, and their expression levels can directly affect the malignant phenotype of PTC-derived BCPAP cells (12). Beyond estrogen, androgen receptors (AR), progesterone receptors, and prolactin receptors are also expressed in PTC lesions (13, 14), and AR activation can exert antiproliferative effects (e.g., inducing cellular senescence) in PTC cell models (15, 16). These findings collectively suggest that SMRGs hold promise as novel molecular targets for diagnostic prediction, endocrine therapy, and prognostic evaluation in both cancers.

Despite certain progress in SMRG research, significant limitations remain. Existing studies have shown that Fu et al. identified the COMP gene, which is highly expressed in both breast cancer and thyroid cancer, and confirmed that its overexpression can promote the occurrence and progression of both cancers through the estrogen signaling pathway (17); Jin et al. found that the ratio of ESR1 to ESR can serve as a prognostic marker for predicting survival in female PTC patients and has the potential to be a therapeutic target (18); Zhang et al. constructed a model by screening estrogen-related differential genes associated with THCA and found that this model is closely related to immune infiltration and therapeutic response in THCA (19). However, current research still has three shortcomings: first, the exploration of SMRGs is insufficiently systematic, with most focusing on estrogen metabolism-related genes in PTC, while neglecting the contribution of other sex hormone metabolism genes (such as those involved in androgen and progesterone metabolism) to the development of thyroid cancer; second, most studies are limited to thyroid cancer alone, failing to incorporate its bidirectional risk characteristics with breast cancer, making it difficult to reflect the cross-regulatory association of hormones between the two; third, some studies only focus on the predictive efficacy of SMRGs in PTC, lacking in-depth analysis of their underlying pathogenic mechanisms (e.g., associations with the tumor microenvironment and therapeutic sensitivity). Therefore, there is an urgent need to systematically explore the prognostic efficacy and potential mechanisms of SMRGs associated with both cancers in PTC from a cross-cancer perspective.

To address the above research gaps, this study integrates transcriptomic data of THCA and BRCA from The Cancer Genome Atlas (TCGA) database, and screens SMRGs directly or potentially associated with both cancers through cross-analysis, providing a molecular basis for deciphering cross-cancer metabolic associations. On this basis, consensus clustering and LASSO Cox regression methods are used to construct a thyroid cancer prognostic model (TBSMRPM). The innovation of this study lies in: analyzing the role of SMRGs for the first time from a cross-disease perspective, overcoming the limitations of single-cancer research; and systematically associating the model with immune infiltration, tumor mutational burden (TMB), and drug sensitivity to provide multidimensional references for clinical treatment. Ultimately, it aims to provide new insights for the prognostic evaluation and treatment strategy optimization of thyroid cancer, while laying a foundation for elucidating the role of SMRGs in cross-cancer regulation.

## 2 Materials and methods

### 2.1 Acquisition and processing of information

**Figure 1:**
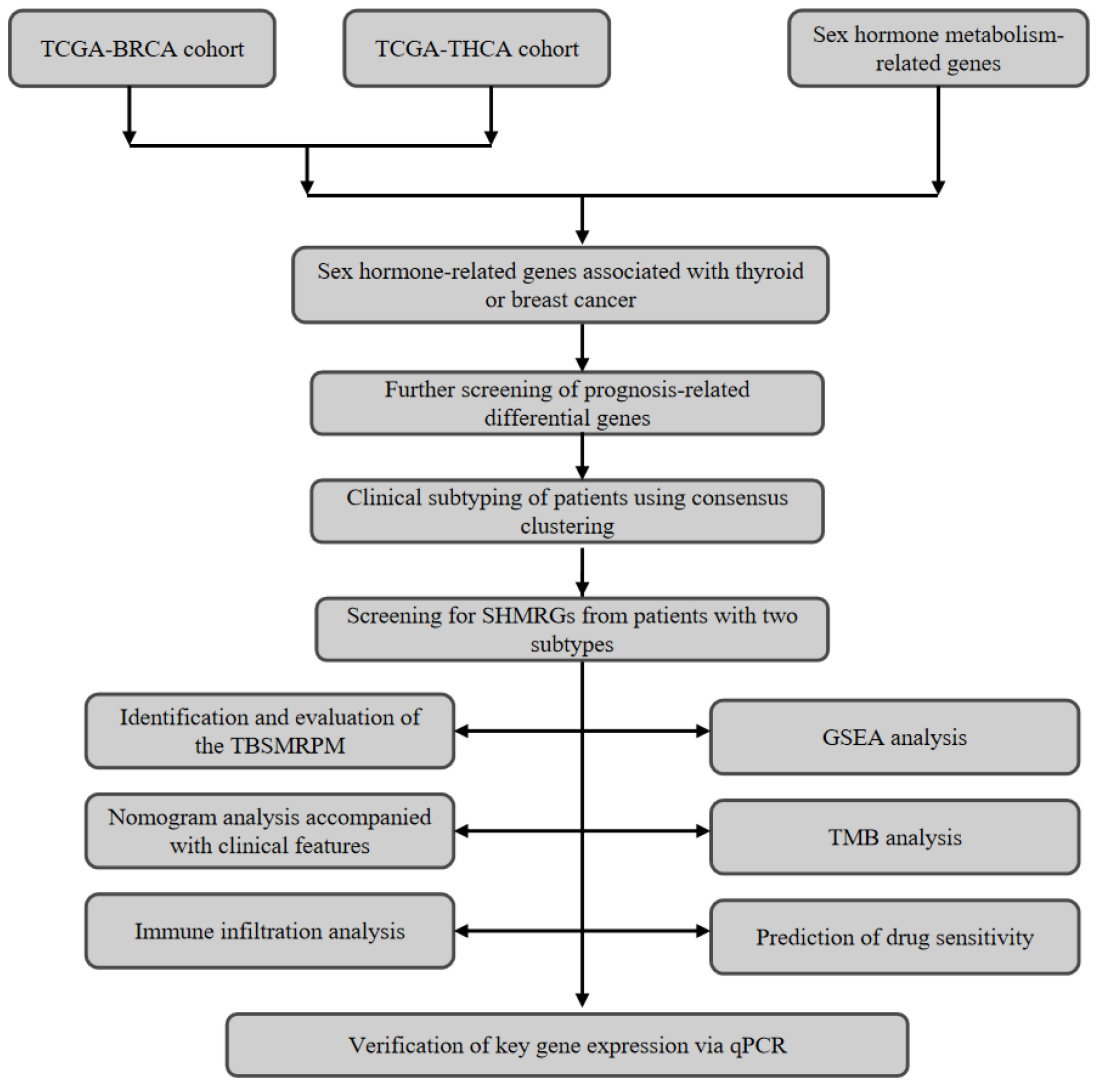
Sex hormone-related gene analysis workflow.

We obtained transcriptomic data for thyroid and breast cancers from the TCGA database and also downloaded clinical and survival information for thyroid cancer. The THCA data lacking DFS survival information were excluded, and the final THCA cohort used for the study included 498 tumor samples and 59 normal samples. The BRCA cohort included 1,118 tumor samples and 113 normal samples. The baseline characteristics table of the patient cohort is shown in **Supplementary Table 1**. We also logged on to MSigDB (Molecular Signatures Database) to obtain 401 sex hormone metabolism-related genes (20). This research was authorized by the Ethics Committee of the First Affiliated Hospital of Zhengzhou University [approval number: 2020-KY-0075-002]. The overall flowchart of this study is shown in **Figure 1**.

### 2.2 Consistent clustering based on gene features

We firstly extracted the different genes of thyroid cancer and breast cancer from the acquired transcriptome data, and then intersected with the sex hormone metabolism-related genes to extract the sex hormone metabolism-related genes associated with thyroid cancer or breast cancer. We further obtained thyroid cancer prognosis-related genes via univariate Cox analysis. By utilizing the “ConsensusClusterPlus” R package, we accomplished consensus clustering on the basis of genetic characteristics for the THCA patients (patients were classified as two subclasses, C1 and C2) (21). The specific steps are as follows: First, z-score standardization was performed on the gene expression profiles to eliminate differences in dimensions. The key parameters were set as follows: the number of repeated sampling (reps) was 500, with 80% of samples randomly selected in each sampling, while all features (pFeature=1) were retained for clustering analysis to ensure the robustness of results against data perturbations. The “km” (k-means clustering) algorithm was used for clustering, with Euclidean distance as the distance measurement method, and the range of potential clustering numbers evaluated was k=2 to k=10. The optimal number of clusters was determined through cumulative distribution function (CDF) curve analysis. And we plotted KM curves to determine the DFS difference between the clusters. After that, we also assessed the differences in immune infiltration between clusters and evaluated the stromal and immune scores utilizing the ESTIMATE algorithm.

### 2.3 Identification of differential genes and establishment of TBSMRPM

We identified differential genes in different clusters using the “Deseq2” package (22). A total of 498 thyroid cancer (THCA) patients were randomly divided into a training set and a test set at a ratio of 5:5, with 249 patients in each of the training set and the test set. We used the training set to screen the optimal differential genes by univariate Cox, LASSO cox and stepwise cox regression analyses, respectively, in which the “glmnet” R package was called (23). The median risk scores established by multivariate Cox regression was utilized for the differentiating of the risk groups. We also plotted risk factor linkage charts and KM curves to show the differences in population proportions, survival (DFS) status, and gene expression across risk groups. The sensitivity and specificity of the above differential genomes are reflected by receiver operating characteristic curves (ROCs).

### 2.4 Exploration of model-clinical correlations

To explore the correlation between TBSMRPM and clinical features, we plotted box line plots of the relationship between risk scores and age, gender, stage, extra-glandular invasion, T stage, N stage, M stage, and tumor burden. Stratified analysis further demonstrated the variability of the risk groups within each clinical subgroup.

### 2.5 Modeling and evaluation of the clinical factor-related nomogram

Our Cox regression analysis included TBSMRPM and clinical factors. The univariate Cox regression analysis was utilized for the screening of model components (24). Furthermore, we plotted ROC curves, calibration curves, and decision curves to fully assess the predictive efficacy, calibration, and clinical utility of the model, respectively.

### 2.6 Immunocorrelation analysis and gene set enrichment analysis

We assessed the correlation of immune cells with risk scores by means of seven algorithms (25–30). The immune checkpoints, immune pathways and immune cells were evaluated between the two risk groups (31). The ESTIMATE algorithm was used for the calculating of immune and stromal scores (32). In addition, changes in pathway activity between different risk groups were analyzed by GSEA (33).

### 2.7 Tumor mutational burden

TMB is the counting of somatic mutation sites in the tumor genome, usually described as mutations per million bases (mut/Mb). This indicator may provide some indication of a tumor’s ability to generate neoantigens and predict the efficacy of tumor immunotherapy (34). After integrating the data, we analyzed the somatic mutations associated with risk groupings using the maftools R package (35).

### 2.8 Drug sensitivity analysis

We screened potential therapeutic agents by correlating drug sensitivity with risk subgroups and invoked the “oncoPredict” R package to complete the calculating of drug half-maximal inhibitory concentration (IC50) (36).

### 2.9 Procedures of quantitative real-time PCR

Our research purchased the human papillary thyroid cancer cell lines (IHH4, KTC-1, TPC-1) and normal thyroid cell line (Nthy ori-3-1) from the Shanghai Cell Biochemical Institute (Shanghai, China). These cell lines were cultured in RPMI-1640 medium (Gemini) containing 10% fetal bovine serum (Gemini). Then the medium was placed in an incubator at 5% CO2 and 37°C for further incubation. The extraction of total RNA was performed utilizing TRIzol reagent (Invitrogen). After that, the Prime Script RT reagent kit with gDNA Eraser (Takara) was utilized for the reverse transcription process. We referred to the instructions to complete the PCR reaction by using the SYBR Green Detection kit (Takara) and utilized the 2^-ΔΔCt^ method to complete the calculation of relative gene expression levels. Detailed primer sequences are shown in **Supplementary Table 4**.

### 2.10 Statistical analysis

All data analyses were finished by R software (version 4.3.1) and GraphPad Prism (version 8.0.2). Differences between the groups were compared using the Wilcoxon test. Kaplan-Meier curves and log-rank tests were utilized for assessment of the DFS discrepancy between the groups. *P*<0.05 was considered statistically significant.

## 3 Results

### 3.1 Construction of the consistent clustering

**Figure 2:**
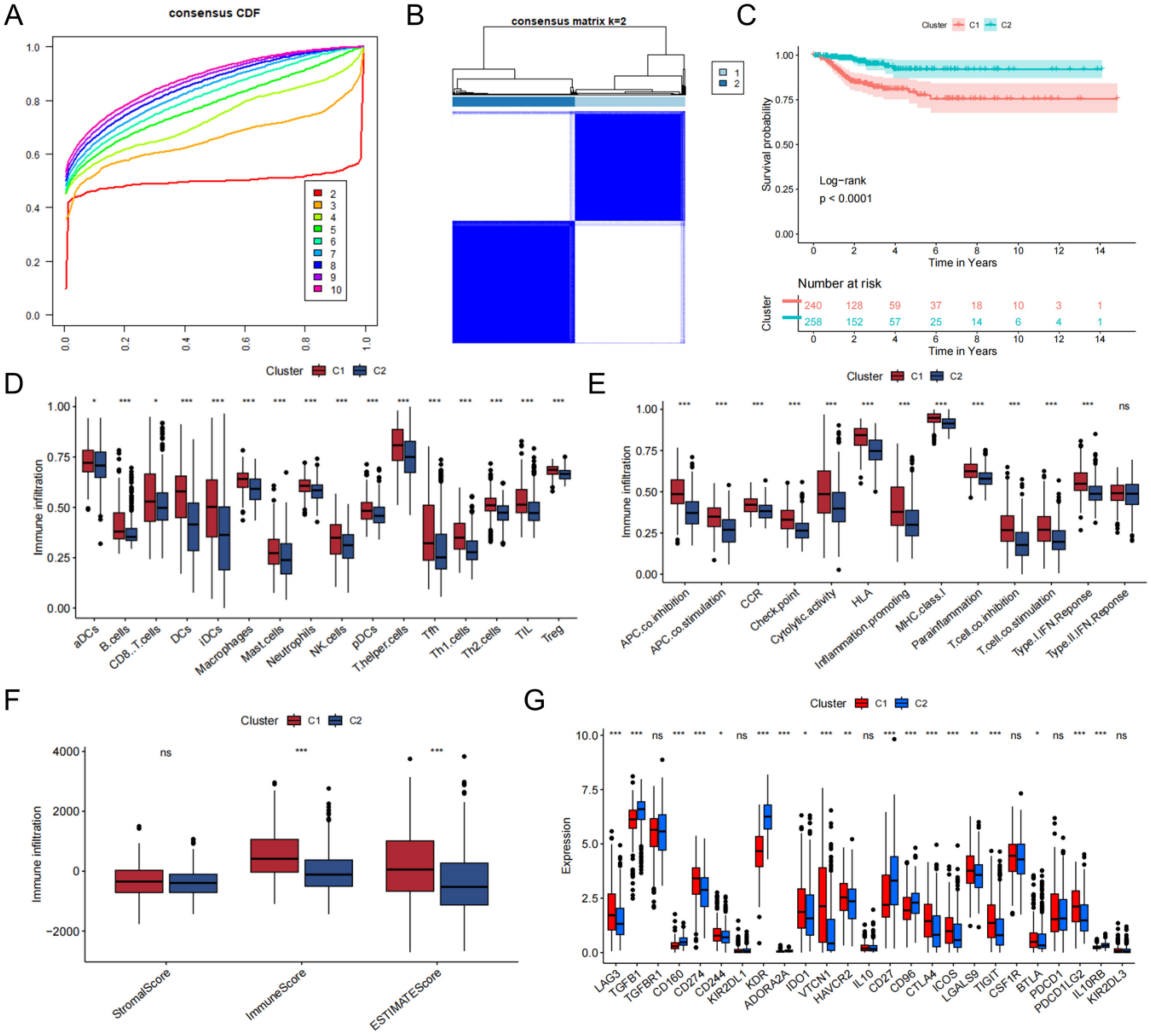
Construction of the consensus clustering. **(A)** The result of consensus cumulative distribution function. **(B)** Determination of the ideal number of clusters. **(C)** K-M curves with between-cluster differences. **(D)** The ssGSEA scores of 16 kinds of immune cells. **(E)** Boxplot presenting significant differences in immune pathway enrichment between the two clusters. **(F)** Correlations between the tumor microenvironment score and the two clusters. **(G)** The presence of differentially expressed immune checkpoints. ns, no significance, *P<0.05; **P<0.01, ***P<0.001.

By screening tumor and normal tissue differential genes from the TCGA-THCA cohort and TCGA-BRCA cohort, and then taking the intersection with sex hormone metabolism-related genes, we obtained a total of 159 sex hormone-related genes associated with thyroid or breast cancer (**Supplementary Table 2**, **Supplementary Figure 1C**). We then extracted 26 differentially expressed genes associated with DFS by univariate Cox regression. According to the gene characteristics, the consistent clustering results showed that the curves showed a clear flattening trend and the best clustering effect when k = 2 (**Figures 2A, B**,**Supplementary Figures 1D, E**). KM curves of the clusters also demonstrated marked differences (**Figure 2C**, **Supplementary Table 3**). Enrichment scores for immune cells were assessed in the two clusters using ssGSEA analysis (**Figure 2D**), which showed that the cells with significant immune infiltration in the two clusters included T helper cells, aDCs, and so on. And the result demonstrated between-cluster variability in infiltration of all immune cells (*P*<0.05). The immune pathway result illustrated that the differential genes of the two clusters were clearly correlated with pathways of MHC class-I, HLA, and parainflammation (**Figure 2E**). The enrichment of the 15 immunization pathways also differed significantly across clusters (*P*<0.05). C1 corresponded to higher stromal and immune scores based on ESTIMATE results (**Figure 2F**). In addition, the immune checkpoints that were significantly expressed in C2 over C1 included TGFB1, KDR, and CD27, and those that were significantly expressed in C1 over C2 included CD274, VTCN1, and LGALS9 (**Figure 2G**).

### 3.2 Identification and evaluation of the TBSMRPM

**Figure 3:**
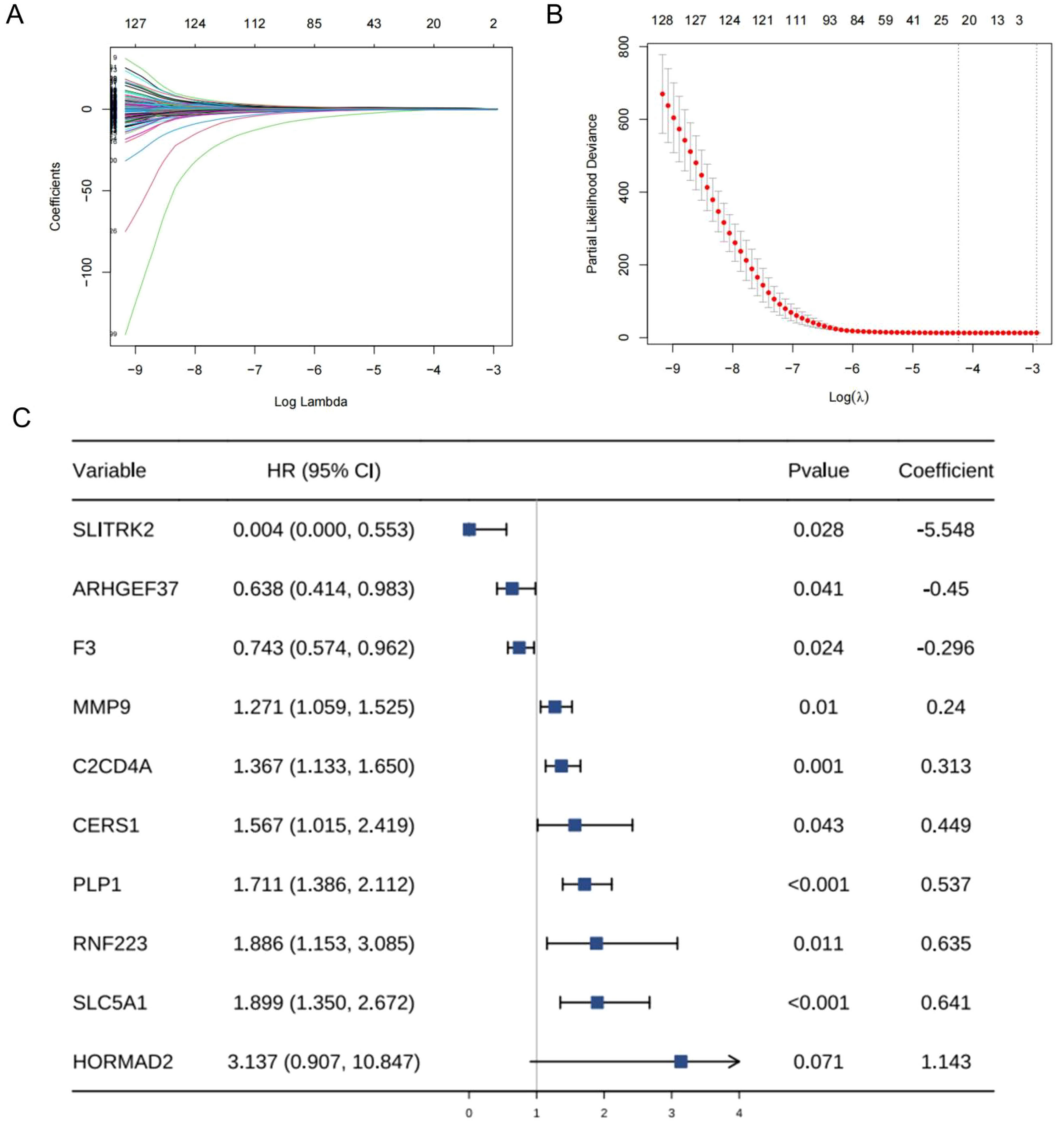
Establishment of sex hormone metabolism-related genes prognostic model. **(A, B)** Parameterization for LASSO analysis. **(C)** Forest plots for stepwise cox analysis.

After consistent clustering, we performed differential gene analysis for both clusters and screened a total of 838 differential genes (**Supplementary Table 4**). Univariate cox regression analysis, LASSO cox regression analysis, and stepwise cox regression analysis was used to screen for the prognostically strong related genes and to fit the optimal model. Ultimately, we screened out a total of 10 differential genes correlated with DFS to establish the TBSMRPM (**Figure 3C**). The formula is as follows: Risk score = (-5.548expression of SLITRK2) + (-0.450* expression of ARHGEF37) + (-0.296* expression of F3) + (0.240* expression of MMP9) + (0.313* expression of C2CD4A) + (0.449* expression of CERS1) + (0.537* expression of PLP1) + (0.635* expression of RNF223) + (0.641* expression of SLC5A1) + (1.143* expression of HORMAD2). A total of 498 THCA patients were randomly divided into a training set and a test set at a ratio of 5:5, with 249 patients in each of the training set and the test set. The expression distributions, survival status and risk scores of the 10 genes are displayed in **Supplementary Figure 2A**. The KM curves of the DFS status of the two groups in the training, test, and total sets showed significant differences (**Supplementary Figure 2B**, **Supplementary Table 3**), and the ROC curves demonstrated the good predictive efficacy of the model (**Supplementary Figure 2C**) (33), the area under the ROC curves (AUCs) of the training-focused model to predict 1-, 3-, and 5-year DFS were 0.914, 0.859, and 0.772, respectively, and in the validation-focused model, they were 0.750, 0.795, and 0.770.

### 3.3 Correlation of TBSMRPM with clinical features

**Figure 4:**
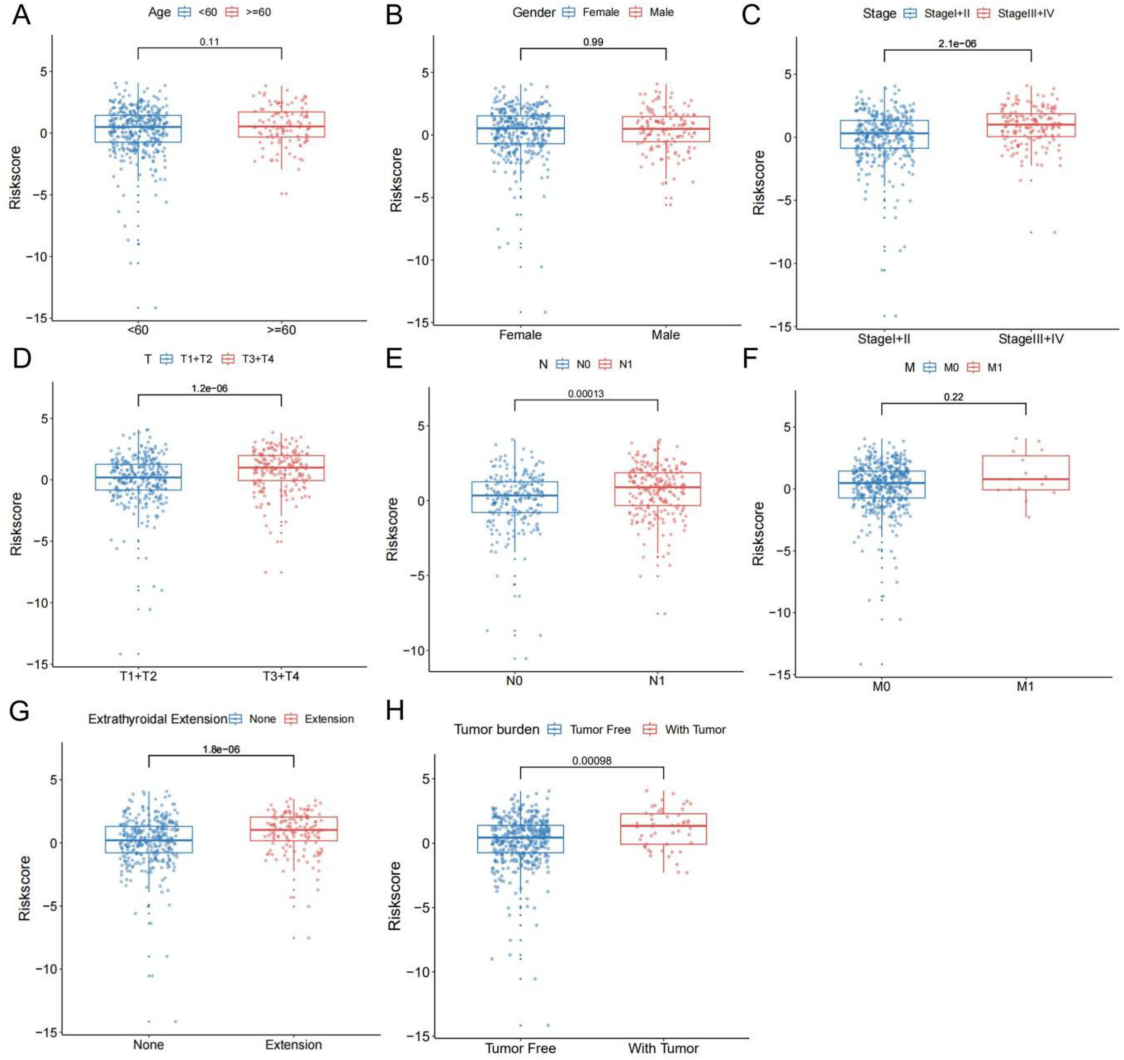
Risk scores of the patients are classified by common clinical features.

**Figure 5:**
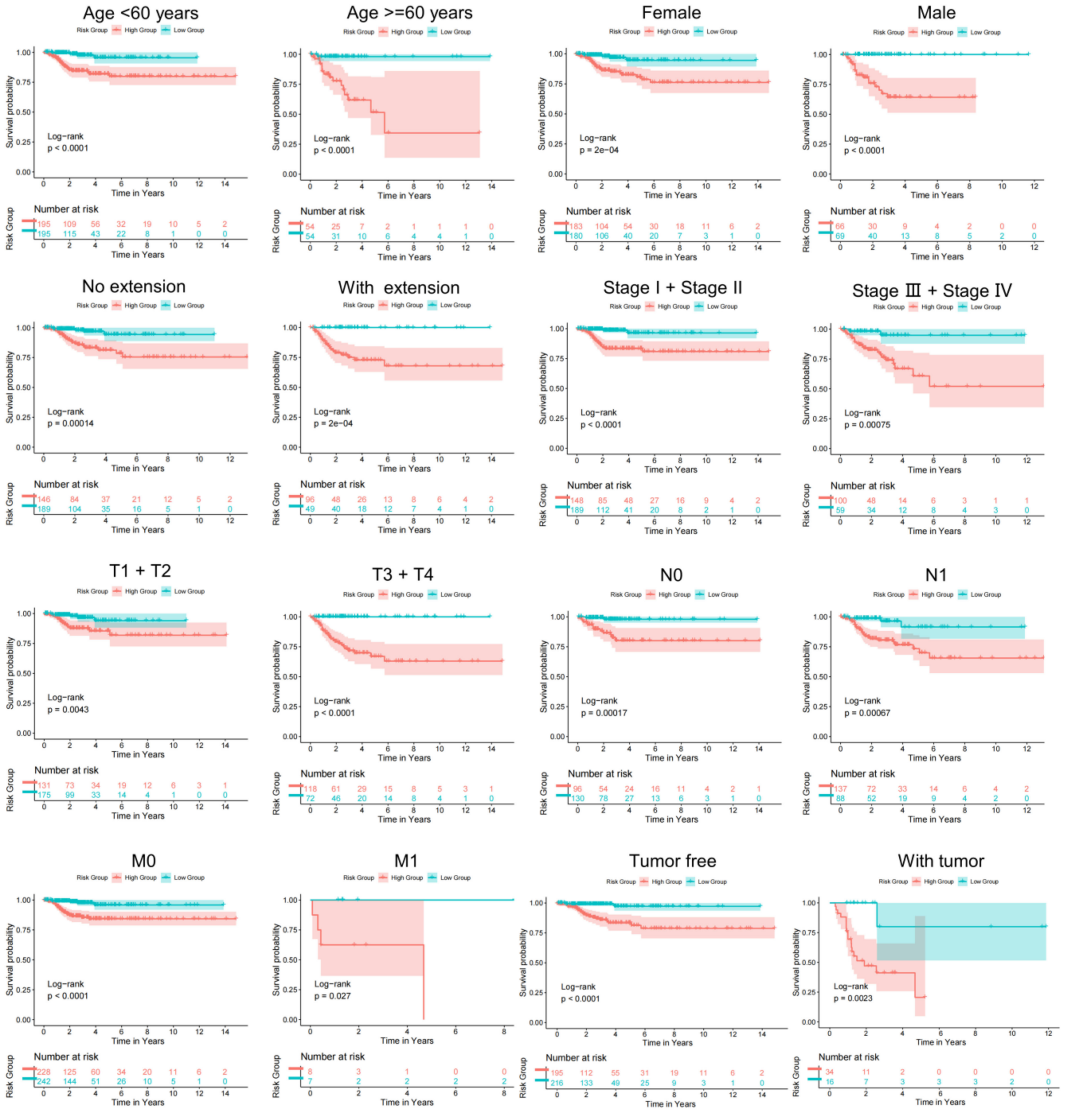
Subgroup KM analysis on the basis of various clinical features.

To determine the value of the model in depth, our research evaluated the correlation of TBSMRPM and important clinical features and found that the risk score was significantly correlated with stage, extra-glandular invasion, T stage, N stage, and tumor burden (**Figure 4**). Then, our study performed stratified analyses of clinical factors, age<60 years, age≥60 years, female, male, absence of extra-glandular invasion, presence of extra-glandular invasion, clinical stage I/II, clinical stage III/IV, T1/T2, T3/T4, N0, N1, M0, M1, absence of tumor burden, and presence of tumor burden, and other subgroup analyses showed significant variability in the DFS status-KM curves among different risk groups (**Figure 5**, **Supplementary Table 3**). These results suggest that TBSMRPM has excellent anticipation ability.

### 3.4 Validation and application of the prognostic model

#### 3.4.1 Integration of clinical factors and evaluation of nomogram performance

**Figure 6:**
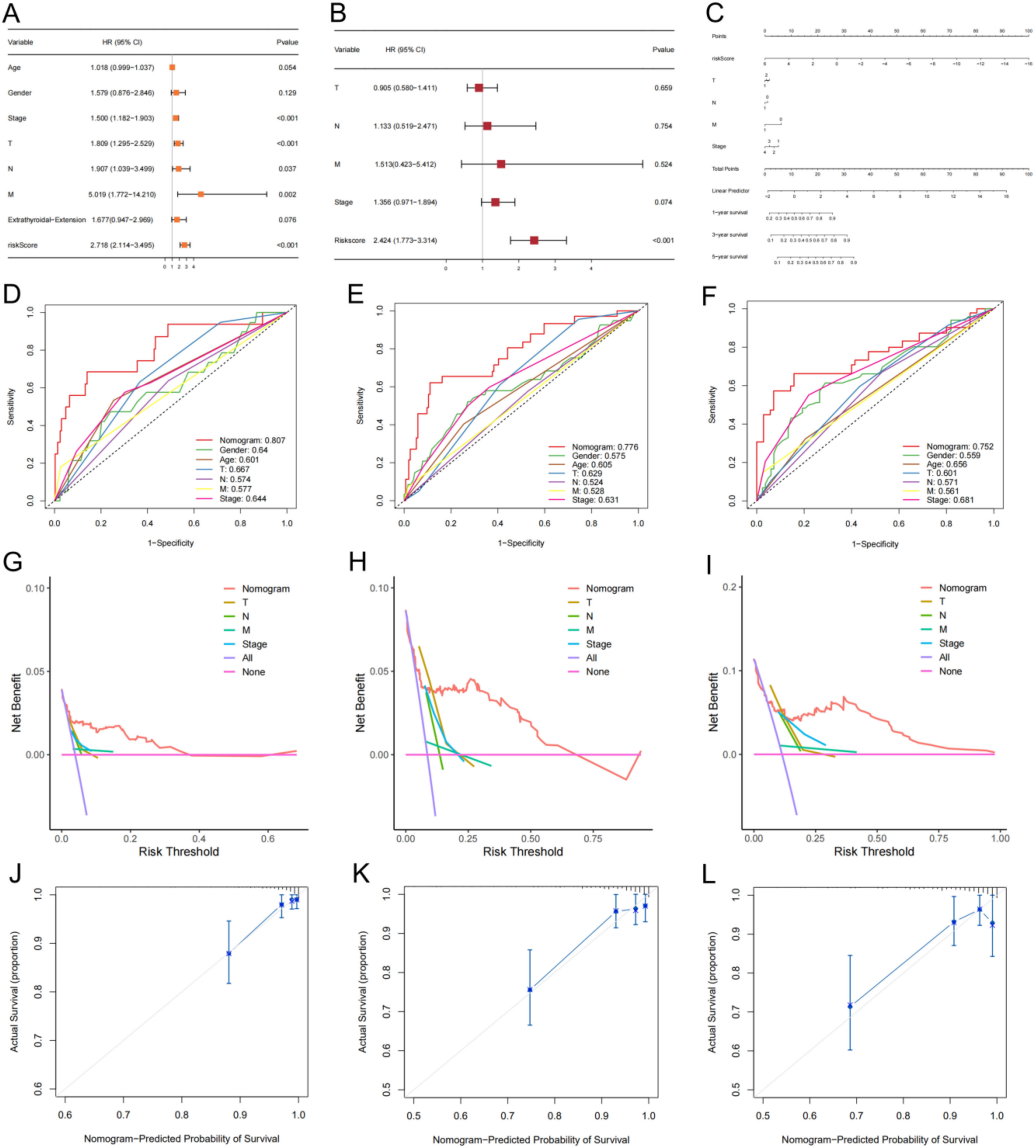
A nomogram model with clinical factors. **(A, B)** Univariate and multivariate Cox analysis results. **(C)** The nomogram for predicting DFS of thyroid cancer in total set. ROCs **(D-F)**, decision curves **(G-I)** and calibration curves **(J-L)** for the nomogram and other features used to predict DFS.

Our analysis results indicated that stage, T stage, N stage, M stage, and risk score were prognosis-related factors (**Figure 6A**). The risk score was established as an independent predictor of prognosis for DFS by multivariate Cox regression analysis (**Figure 6B**). Subsequently, we incorporated stage, T stage, N stage, M stage, and risk score into the model to predict DFS status of thyroid cancer patients (**Figure 6C**). ROC results indicated that the AUCs of the nomogram for predicting DFS status at 1, 3, and 5 years were 0.807 (**Figure 6D**), 0.776 (**Figure 6E**), and 0.752 (**Figure 6F**), respectively. Both decision curves (**Figures 6G, I**) and calibration curves (**Figures 6J–L**) indicated the ideality of the predictive modeling.

**Figure 7:**
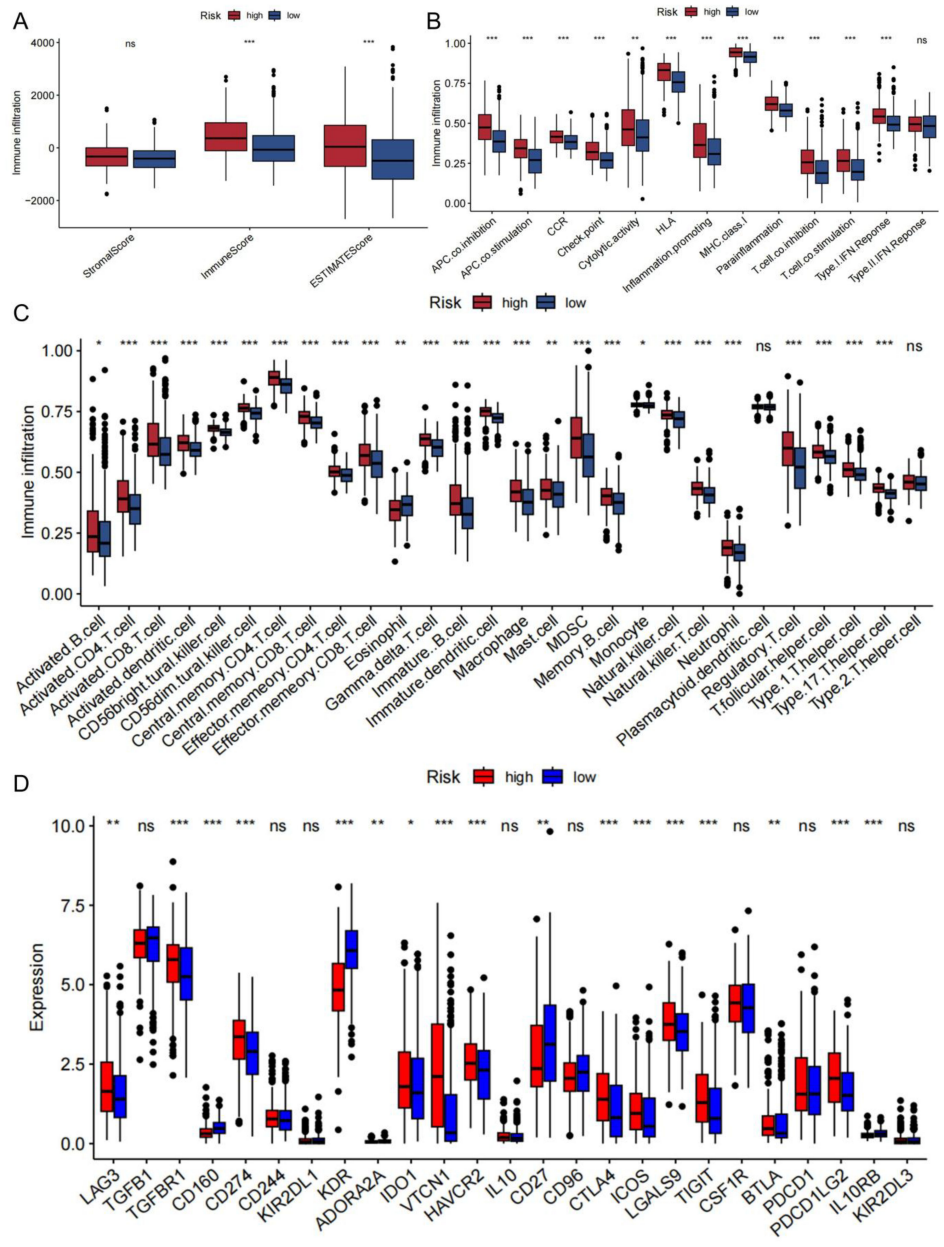
Immunocorrelation results of the two risk groups. **(A)** Correlations between the tumor microenvironment score and different risk groups. **(B)** Boxplot for differential analysis of immune pathway enrichment. **(C)** Boxplot presenting the significant differences in 28 types of immune cell enrichment between different risk groups. **(D)** Boxplot for differential analysis of immune checkpoint expression. ns, no significance, *P<0.05; **P<0.01, ***P<0.001.

The results of the seven immune algorithms indicated that the risk scores were negatively correlated with cells such as endothelial cells, CD8 T cells, and hemapoietic stem cells (coefficient < -0.3), and positively correlated with NKT cells, sebocytes, and macrophages M0 relationship (coefficient > 0.2) (**Supplementary Figure 3**). The ESTIMATE result indicated that the high-risk group corresponded to higher ESTIMATE scores (**Figure 7A**), which suggested a higher level of immune infiltration. The immune pathway analysis showed that the high-risk group was significantly enriched in Parainflammation, MHC class-I and HLA pathways (**Figure 7B**). Meanwhile, 12 immune pathways such as co-inhibition, APC co-stimulation, and CCR were significantly different between the TBSMRPM groups. The CD4 T cells, CD56 dim tural killer cells, etc., also showed significant differences between different risk groups, and a total of 26 immune cell infiltrations were significantly different between the two risk groups (**Figure 7C**). Differential expression analysis of immune checkpoints in different risk groups showed that the immune checkpoints more significantly expressed in the low-risk group included CD160, KDR, CD27, and so on. The more significantly expressed immune checkpoints in the high-risk group included LAG3, TGFBR1, LGALS9 and so on (**Figure 7D**).

#### 3.4.2 Correlation with tumor immune microenvironment and functional pathway enrichment

**Figure 8:**
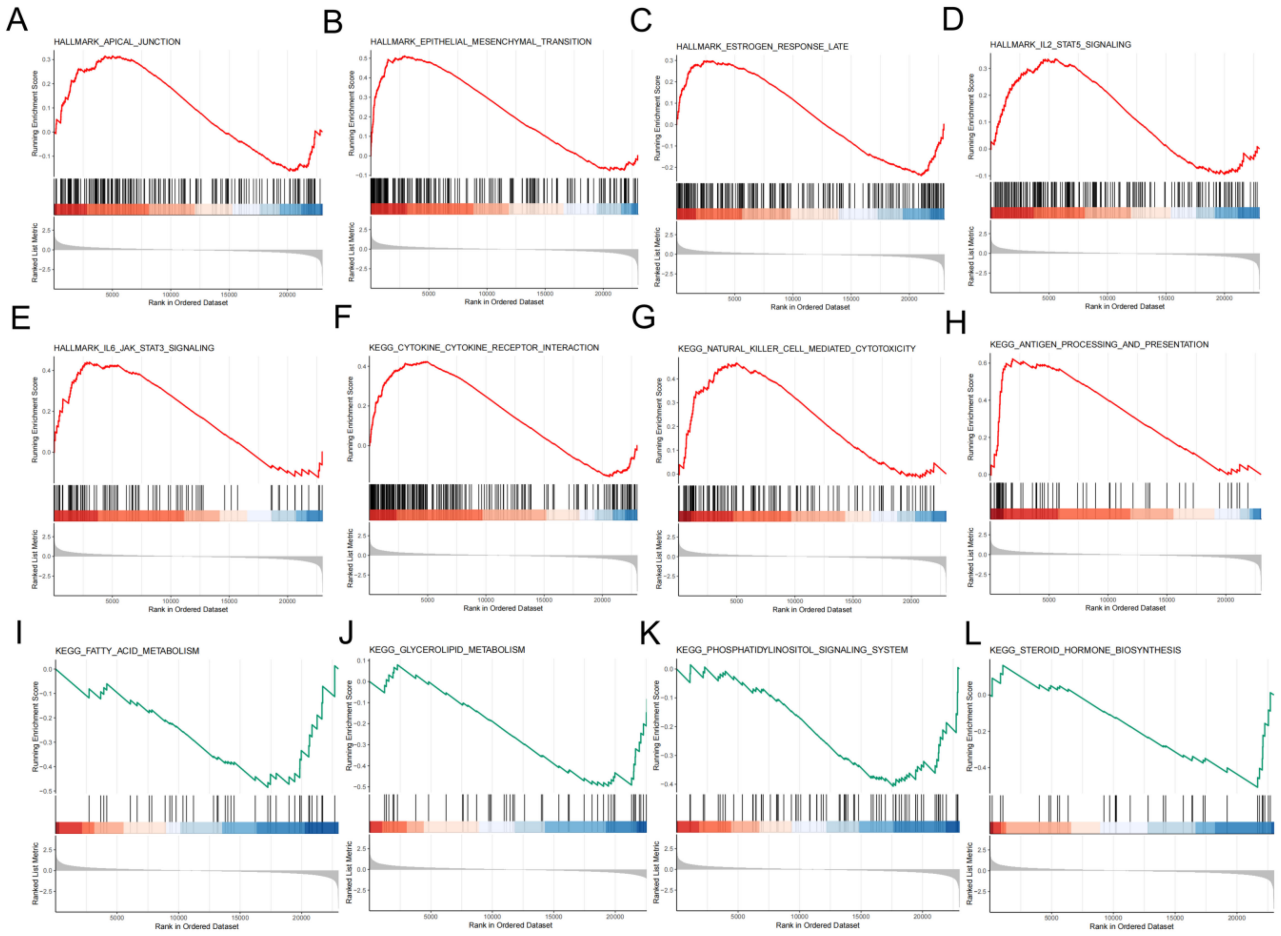
An in-depth exploration of the role of molecules. Significantly enriched signaling pathways in high-risk group **(A-G)** and low-risk group **(I-L)** in accordance to GSEA.

To reveal gene expression patterns associated with specific biological processes, pathways or functions, our study performed GSEA analysis and it showed that apical junction, estrogen response late, antigen processing and presentation signaling pathways and other pathways had important roles in the high-risk group (**Figures 8A–H**), which are important in the development and progression of malignant tumors. While signaling pathways such as fatty acid metabolism, glycerolipid metabolism, phosphatidylinositol signaling system, and steroid hormone biosynthesis were significant in the low-risk group (**Figures 8I–L**).

#### 3.4.3 Association with tumor mutational burden and prognostic implications

**Figure 9:**
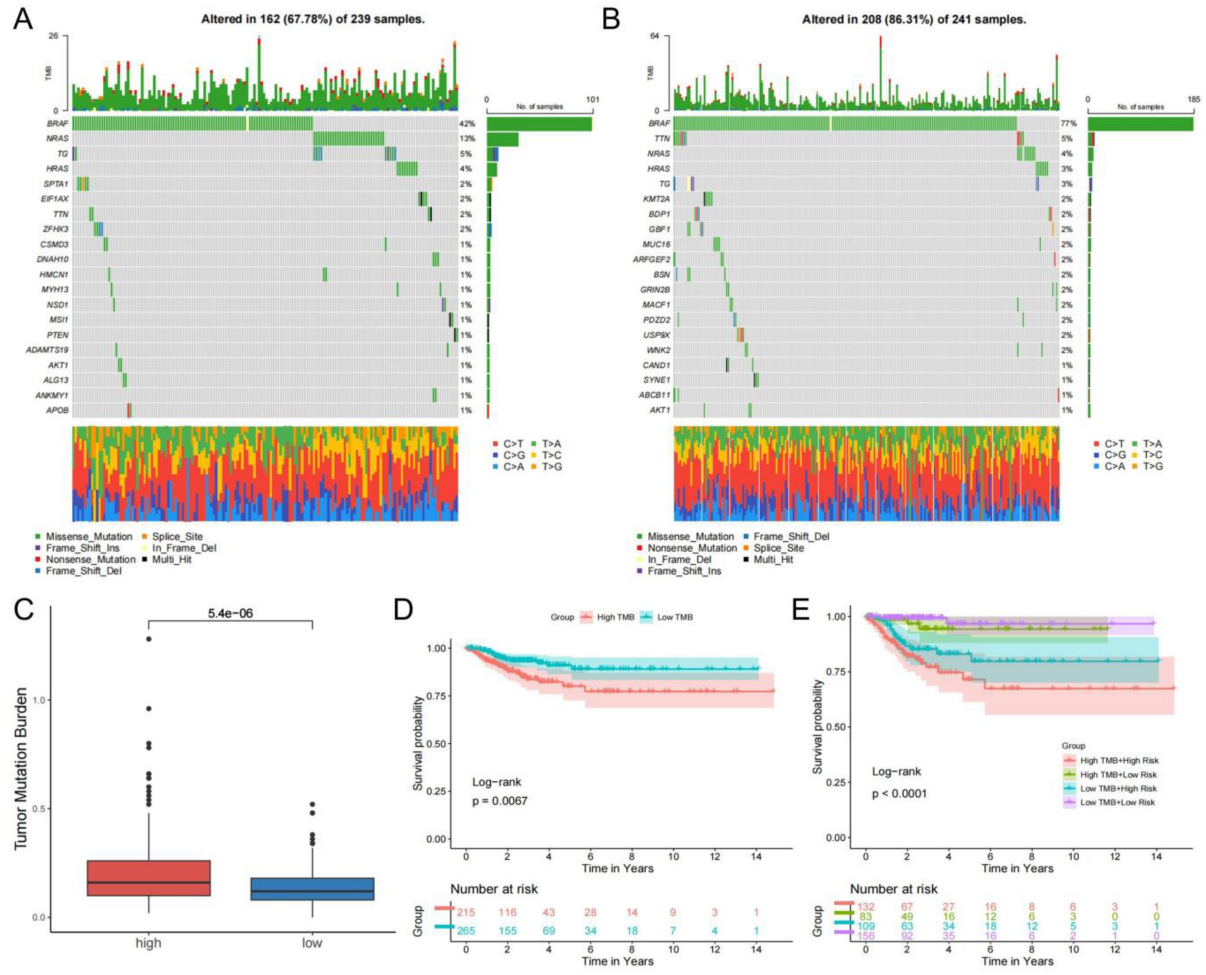
Differential analysis of TMB. The mutations landscape of low-risk group **(A)** and high-risk group **(B)**. **(C)** Relevance of TMB to risk groups. **(D)** Differences in DFS of TMB groups. **(E)** Comparison of KM curves for the combined TMB and risk groups.

The waterfall plot indicated that the rank order of frequency of somatic mutations in the low-risk group was BRAF (42%), NRAS (13%), TG (5%), HRAS (4%), and other genes were mutated in less than 4% of the genes (**Figure 9A**). The rank order of frequency of somatic mutations occurring in the high-risk group was BRAF (77%), TTN (5%), NRAS (4%), and other genes had a mutation frequency of less than 4% (**Figure 9B**). The somatic mutations in both groups were predominantly missense mutations (**Supplementary Figures 4A, B**). The box line plot visualized the significant variability of somatic mutations between the risk groups, and the high-risk group had higher TMB values (**Figure 9C**). Distinguishing the high and low TMB groups by the median somatic mutation scores of all patients showed a significant difference in KM analysis of DFS status between the two groups (**Figure 9D**, **Supplementary Table 3**). The KM curve analysis of the combined TMB group and risk group still showed a significant difference between the four groups (**Figure 9E**, **Supplementary Table 3**). In addition, **Supplementary Figure 4C** demonstrates that risk score and TMB were significantly correlated (R=0.25, *P*<0.0001). Our findings revealed that poor prognosis in the high-risk group may be associated with higher TMB.

#### 3.4.4 Prediction of drug sensitivity based on risk stratification

**Figure 10:**
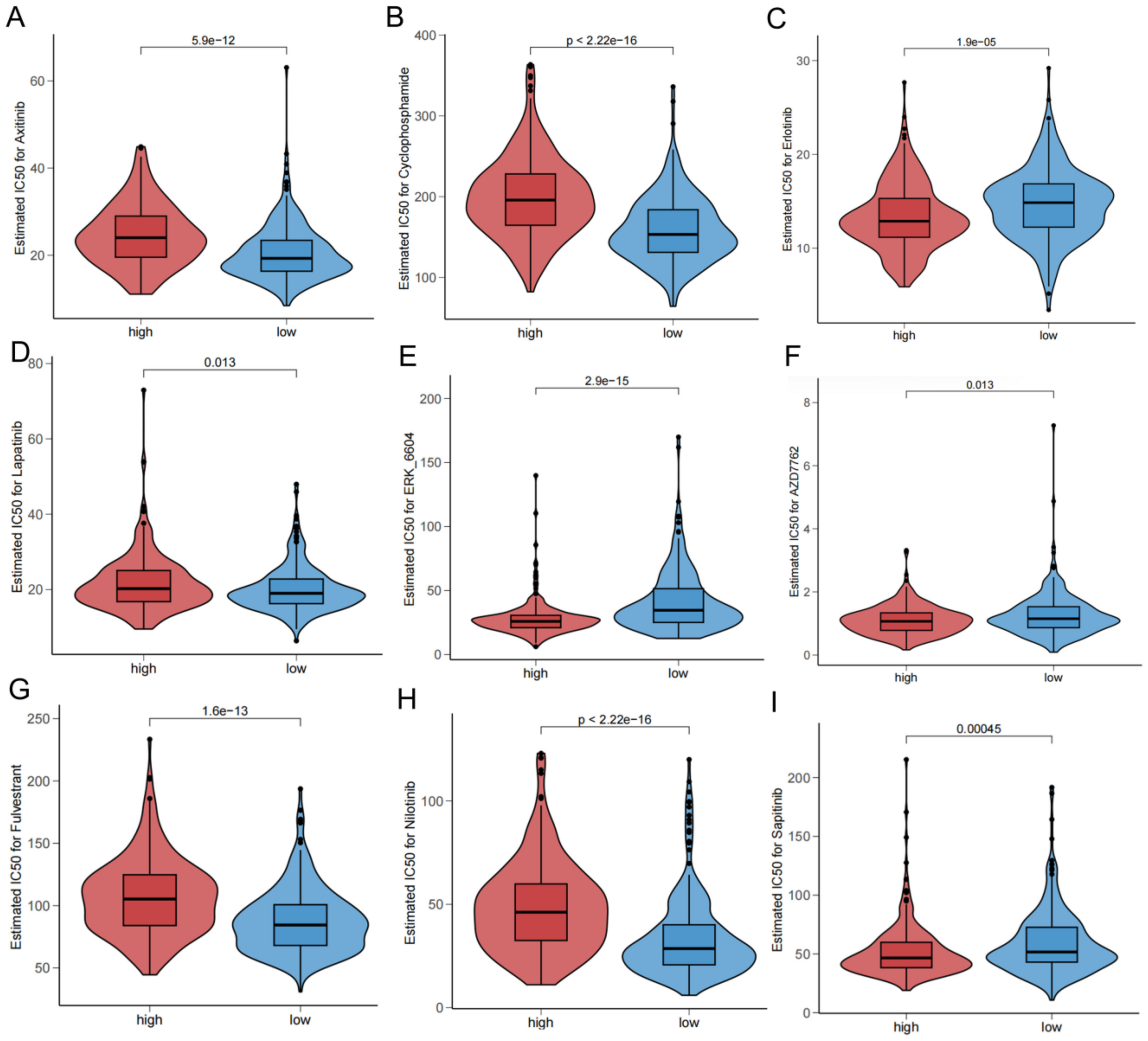
Drug sensitivity in different risk groups.

The drug sensitivity analysis revealed that Axitinib (**Figure 10A**), Cyclophosphamide (**Figure 10B**), Lapatinib (**Figure 10D**), fulvestrant (**Figure 10G**), nilotinib (**Figure 10H**) were more sensitive in the low-risk group, and Erlotinib (**Figure 10C**), ERK-6640 (**Figure 10E**), AZD7762 (**Figure 10F**), and Sapitinib (**Figure 10I**) were more sensitive in the high-risk group.

### 3.5 PCR for determining gene expression

**Figure 11:**
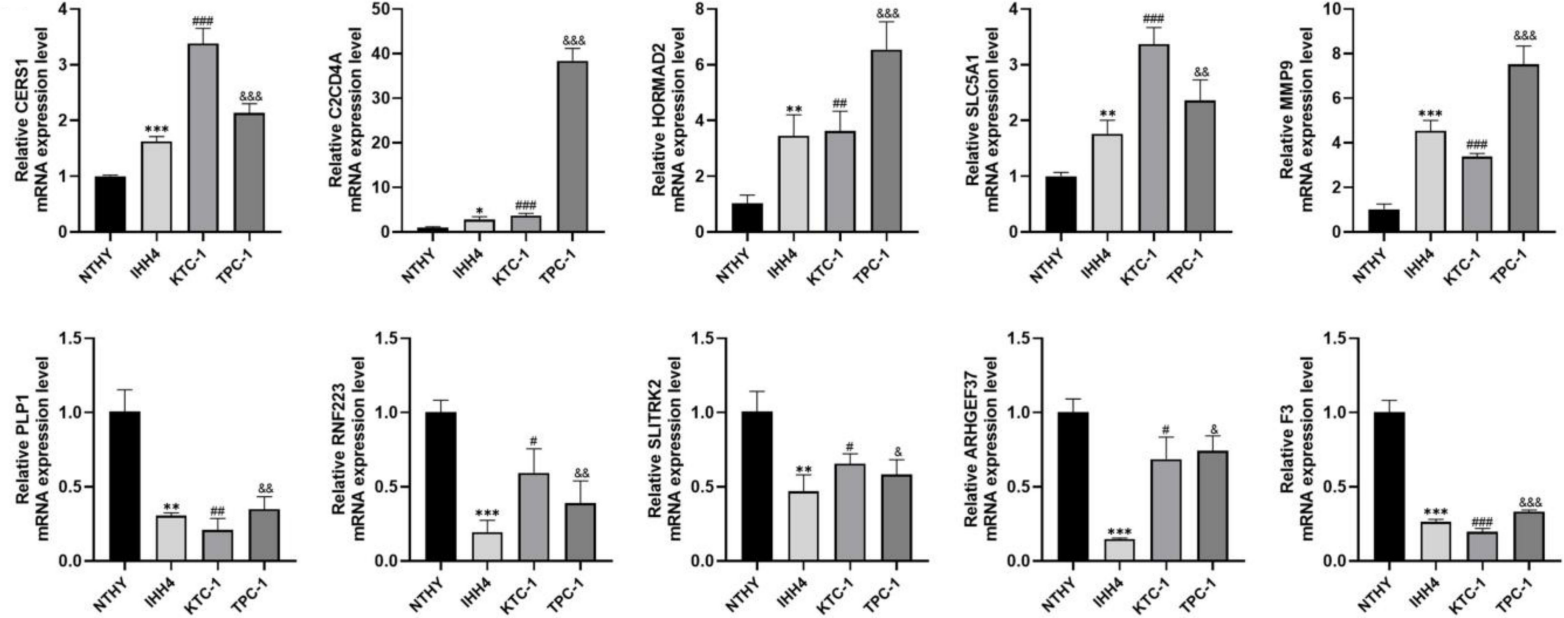
Expression of 10 genes in IHH4, KTC-1, TPC-1 human papillary thyroid cancer cell lines and human thyroid normal cell line Nthy ori-3-1. * indicates P<0.05, ** indicates P < 0.01, *** indicates P < 0.001; ^#^ indicates P<0.05, ^##^ indicates P<0.01, ^###^ indicates P<0.001, ^&^ indicates P<0.05, ^&&^ indicates P<0.01, ^&&&^ indicates P<0.001.

We examined the expression of 10 genes in IHH4, KTC-1, TPC-1 human papillary thyroid carcinoma cell lines and human thyroid normal cell line Nthy ori-3-1 by PCR analysis. The results showed that *C2CD4A*, *CERS1*, *MMP9*, *SLC5A1*, and *HORMAD2* were highly expressed in IHH4, KTC-1, and TPC-1 cell lines, and *SLITRK2*, *ARHGEF37*, *PLP1*, *RNF223*, and *F3* were lowly expressed in IHH4, KTC-1, and TPC-1 cell lines (**Figure 11**). The associations between the 10 genes and sex hormone metabolism are detailed in **Supplementary Table 8**.

## 4 Discussion

Numerous studies have shown that there is a potential, bidirectional pathogenic relationship between thyroid cancer and breast cancer, and that the development of one malignant tumor may increase the risk of developing the other (37–39). Of interest, although both malignancies are highly prevalent in the female population, the simultaneous occurrence of both diseases is more common in men (40). This suggests that sex hormones may serve as a vital role in the tumorigenesis and progression of thyroid cancer. However, studies on the prognostic relationship between sex hormones and thyroid cancer are still very limited (2, 41). In our research, we constructed a prognostic model for the first time on the basis of sex hormone metabolism-related genes in thyroid cancer and breast cancer, which was able to better predict the prognostic (DFS) profile of thyroid cancer patients.

We first screened for genes correlated with sex hormone metabolism. Notably, considering the potential bidirectional pathogenic relationship between thyroid and breast cancers, we could not ignore the possible indirect effects of genes with altered expression only in a single malignancy on the other tumor. Thus, we included genes related to sex hormone metabolism associated with thyroid or breast cancer together in our study. This idea is different from the study of Duan et al. (42). Subsequently, we classified thyroid cancer patients into two subtypes using consensus clustering. Since the core basis of consensus clustering lies in the similarity of expression patterns of “sex hormone metabolism-related genes”, the algorithm automatically clusters samples with more similar expression patterns into the same group. Therefore, the division of C1 and C2 inherently implies “distinguishable differences in the overall expression profiles of sex hormone-related genes”—this serves as the premise for the stable output of 2 subtypes by clustering. In addition, we utilized existing analyses (pathway enrichment, immune microenvironment, and prognosis association) to cross-validate the expression differences of sex hormone-related genes at the functional level. Furthermore, the significant differences in prognosis and immune infiltration between different subtypes can provide references for clinical staging. To further screen the more desirable prognostic genes and fit an excellent prognostic model, we performed univariate Cox, LASSO Cox and stepwise cox regression analyses after differential gene analysis of subtypes. The ROC results of both training and validation sets suggested a better predictive performance of the model. And the significant survival difference results in the clinical correlation study indicated the rationality of the TBSMRPM risk grouping based on TBSMRPM. We further established a nomogram model in accordance with clinical factors for the prognostic prediction.

In addition, understanding the tumor immune microenvironment contributes to the diagnosis and treatment of thyroid cancer (43). Various cells in the tumor microenvironment such as: endothelial cells, fibroblasts, and infiltrating immune cells serve as vital roles in the processes of tumorigenesis, invasion, and metastasis. In our results, activated dendritic cells, activated CD8+ T cells, Th1 cells, and macrophages were significantly infiltrated in the high-risk group, which may be linked to the adverse prognosis. Relevant research has shown that dendritic cells may result in the tumor progression by affecting the tumor angiogenesis and causing immune dysregulation (44), whereas significant infiltration of activated CD8+ T cells, Th1 cells suggests a higher risk of recurrence (45–47), and significant infiltration of macrophages in thyroid cancer is strongly linked to tumor invasion and short survival (48). The immune pathway analysis illustrated that the risk grouping was strongly associated with the MHC class I pathway. Angell et al. demonstrated that the absence of MHC class I expression was a vital mechanism of immune escape in thyroid malignancies, which highly overlapped with our findings (49). The immune checkpoint results demonstrated the strong correlation of risk groupings with some immune checkpoints. The study by Park et al. also confirmed that these differential genes associated with immune escape signaling were overexpressed in patients with advanced thyroid cancer, which could help to predict the recurrence of advanced thyroid cancer (47). In conclusion, our immune correlation analysis demonstrated the variability of immune function between different subtypes and risk groups in the consistent clustering. The different risk subgroups should be treated with different therapeutic strategies, and the rational formulation of immunotherapy can help to improve the efficacy of the treatment and prevent the development of drug resistance (50).

In the subsequent GSEA analysis, we found that signaling pathways such as fatty acid metabolism, glycerolipid metabolism, phosphatidylinositol signaling system, and steroid hormone biosynthesis were significantly enriched in the low-risk group. It is well known that sex hormones are important members of the steroid family, and their synthesis and metabolism are inextricably linked to these signaling pathways. Sun et al. found that increased fatty acid metabolism was linked to the decreased infiltration of T- and B-cells in male breast cancer and other cancer types, and that targeting fatty acid metabolism pathways may alleviate the immunosuppressive microenvironment in a variety of cancers (51). Lu et al. demonstrated the importance of lipid metabolism in thyroid cancer (52). Liu et al. experimentally verified that pyruvate carboxylase is closely associated with tumor aggressiveness in thyroid cancer by stimulating fatty acid synthesis (53). The phosphatidylinositol (PI) signaling system is a key cellular signaling pathway concerning the cellular processes such as cell growth, differentiation, survival, and intracellular communication, and dysregulation of the related signaling pathway is associated with thyroid cancer and breast cancer (54, 55). Furthermore, a small number of studies examining the association of other sex hormones with thyroid cancer, in addition to estrogen-related studies, have also yielded positive findings (56, 57).

After that, we performed somatic mutation and drug sensitivity analysis. And we found the high-risk group had a significantly higher frequency of BRAF gene mutations. The high-risk group had a higher TMB value and corresponded to the poor prognosis. Studies have confirmed the importance of the detection of BRAF gene mutations in the diagnosis, treatment and determination of prognosis of thyroid cancer (58). This is consistent with the results of our study. There are fewer studies related to other genes with higher mutation frequencies such as NRAS in our findings (59), which found that NRAS mutations were linked to the high risk of distant metastasis of thyroid cancer (60, 61). These findings suggest, to some extent, the potential role of this gene in thyroid cancer. Furthermore, we gained more insight into the correlation of drug sensitivity between different risk groups.

Ultimately, we screened 10 ideal prognosis-related genes. Among them, MMP9 has been shown to promote thyroid cancer incidence and progression in a large number of literatures (62, 63), which is consistent with our findings. HORMAD2 has been shown to inhibit the incidence and progression of thyroid cancer and its hypermethylation promotes the progression of thyroid cancer in a study by Lin et al. (64). Han et al. mentioned that the mRNA level of C2CD4A was dysregulated in the lung cancer (65). Rong et al. confirmed that C2CD4A was highly expressed in colorectal cancer tissues and contributed to tumor growth by inhibiting the P53 signaling pathway (66). Chen et al. verified that *CERS1* was highly expressed in colorectal cancer by PCR (67). Xu et al. found that CERS1 was significantly downregulated in non-small-cell lung cancer cell lines and brain metastatic tissue and its upregulation corresponded to better prognosis (68). Lei et al. proposed that RNF223 could promote pancreatic cancer growth and migration, and identified potential protein targets and metabolism-related pathways (69). Zhang et al. demonstrated that ARHGEF37 promotes hepatocellular carcinoma outgrowth and metastasis through activation of Cdc42 (70). SLC5A1 has been confirmed to be highly expressed in many malignant tumors by several studies, including our research, and a study on pancreatic cancer demonstrated that SLC5A1 regulates cancer cell growth through AMPK/mTOR signaling (71). However, studies on the association of these genes with thyroid cancer have rarely been reported and need to be further explored.

There are some limitations to this study. First, due to limited sample acquisition, we performed PCR validation only in papillary thyroid carcinoma. Second, the model established in this study lacks validation in the external cohorts. Third, the actual role of the screened genes in thyroid cancer lacks sufficient experimental validation. In conclusion, by screening differential genes and establishing a prognostic model, we mined potential novel biomarkers related to thyroid and breast cancers, which are highly suggestive for prognostic prediction of thyroid cancer. However, the actual clinical value of the model needs to be further validated in large cohort studies, and the actual roles and specific mechanisms of these potential biomarkers in malignant tumors need to be further determined by a large number of *in vivo* and ex vivo experiments.

## Data Availability

The original contributions presented in the study are included in the article/**Supplementary Material**. Further inquiries can be directed to the corresponding author.
