# Supplementary material for: Multi-disease transcriptomic analysis of sex hormone genes reveals a novel prognostic model for thyroid cancer with breast cancer correlations
Source: Front Oncol. 2025 Sep 2;15:1641195. doi: 10.3389/fonc.2025.1641195 (PMC12436100; doi:10.3389/fonc.2025.1641195)
Supplement: Supplementary file 1 [file DataSheet1.docx]

Supplementary Materials

# Supplementary Table 1. Demographic information of TC patients in the present study.

| Variables | Number |
| --- | --- |
| Total | 498 |
| Age |  |
| <60 | 390 |
| ≥60 | 108 |
| Gender |  |
| Female | 363 |
| Male | 135 |
| Stage |  |
| I | 140 |
| II | 166 |
| III | 172 |
| Ⅳ | 18 |
| NA | 2 |
| lymph node metastasis |  |
| Yes | 225 |
| No | 226 |
| NX | 47 |
| Metastasis |  |
| Yes | 15 |
| No | 470 |
| NX | 13 |
| TNM stage |  |
| I | 287 |
| II | 50 |
| III | 106 |
| Ⅳ | 53 |
| NA | 2 |
| Tumor burden |  |
| With tumor | 50 |
| Tumor free | 411 |
| NA | 37 |
| Extrathyroidal extension |  |
| Yes | 145 |
| No | 335 |
| NA | 14 |

# Supplementary Table 2. Univariate analysis of 159 differential genes.

| **Genes** | **HR** | **lower** | **upper** | **P value** |
| --- | --- | --- | --- | --- |
| CD38 | 0.922376413 | 0.666849281 | 1.275817897 | 0.625404034 |
| FKBP4 | 0.895252729 | 0.599293608 | 1.337370262 | 0.588961472 |
| BRCA1 | 1.327386241 | 0.85621093 | 2.057850666 | 0.205507525 |
| CALCOCO1 | 1.222084797 | 0.765444259 | 1.951143054 | 0.400804186 |
| ATP1A2 | 0.567407707 | 0.36985022 | 0.87049159 | 0.009455313 |
| CYP46A1 | 0.803715472 | 0.379029686 | 1.704242656 | 0.568820181 |
| DKK3 | 0.705445315 | 0.501180776 | 0.992961256 | 0.045450488 |
| GAL | 1.558933138 | 0.765171953 | 3.176112922 | 0.221397865 |
| FGF10 | 0.816535805 | 0.389771031 | 1.710570229 | 0.591139856 |
| ABCB11 | 0.131185515 | 0.000175441 | 98.0937314 | 0.547426374 |
| DHRS9 | 1.044366545 | 0.809327086 | 1.347664622 | 0.738599953 |
| PTGS2 | 1.052376921 | 0.858522586 | 1.290003552 | 0.623098835 |
| EDN1 | 0.853756099 | 0.641281832 | 1.136628919 | 0.278870162 |
| ADCYAP1R1 | 0.756146647 | 0.54191779 | 1.055063631 | 0.100053449 |
| PGR | 1.053034607 | 0.758909855 | 1.461150986 | 0.757157011 |
| HMOX1 | 0.976541784 | 0.763643208 | 1.248795047 | 0.849943571 |
| RHOXF1 | 0.927019216 | 0.293481402 | 2.928174055 | 0.897249393 |
| SFRP1 | 0.950833153 | 0.79256083 | 1.140712043 | 0.587314298 |
| LHB | 0.795389786 | 0.449570032 | 1.407222161 | 0.431625337 |
| TGFB1 | 1.113171069 | 0.781483631 | 1.5856376 | 0.552528198 |
| ATP1A3 | 0.227394026 | 0.004054615 | 12.75288598 | 0.470985552 |
| CAV1 | 0.833654232 | 0.676665623 | 1.027064706 | 0.087432494 |
| EZH2 | 3.396794175 | 1.94547879 | 5.930782039 | 1.71E-05 |
| GATA3 | 0.982583447 | 0.76631641 | 1.259884583 | 0.889823505 |
| SLC6A4 | 1.835079392 | 0.734111911 | 4.58719757 | 0.194037479 |
| AREG | 1.00484705 | 0.839913046 | 1.202169199 | 0.957844765 |
| CYP27B1 | 1.105011702 | 0.822684085 | 1.484228131 | 0.507106552 |
| ENO2 | 1.428730866 | 0.939948355 | 2.171685153 | 0.094904448 |
| ADTRP | 0.946702562 | 0.774324789 | 1.157454537 | 0.593281987 |
| PDE8B | 0.972648171 | 0.78695749 | 1.202154472 | 0.797505256 |
| NR3C1 | 0.848416872 | 0.561850443 | 1.281143759 | 0.434365142 |
| CLCN2 | 0.778468761 | 0.392969916 | 1.54213742 | 0.472752806 |
| TACR1 | 0.787956659 | 0.358532217 | 1.731715222 | 0.553061709 |
| IGFBP2 | 0.670674621 | 0.501836709 | 0.896316349 | 0.006939414 |
| KDM5B | 1.198100203 | 0.752297667 | 1.908079953 | 0.446529928 |
| SGK1 | 0.832967281 | 0.640664484 | 1.08299197 | 0.172363173 |
| KLF9 | 0.739949815 | 0.542240949 | 1.009746182 | 0.05758888 |
| SOCS2 | 0.506511732 | 0.354435553 | 0.7238386 | 0.000188323 |
| KIF18A | 1.005947697 | 0.627876415 | 1.61167189 | 0.980327096 |
| CRY2 | 0.717360801 | 0.507031589 | 1.01493976 | 0.060627127 |
| CAT |  | 0.589573349 | 0.996840877 | 0.047302639 |
| AKR1D1 | 4.752841336 | 0.470284355 | 48.03370672 | 0.186588655 |
| PMEPA1 | 0.811346477 | 0.565911195 | 1.163226867 | 0.255379477 |
| PCK1 | 1.805199664 | 0.557467883 | 5.845620754 | 0.324499504 |
| TRERF1 | 0.871499552 | 0.53769072 | 1.412543383 | 0.576706745 |
| SCGB2A1 | 1.045299781 | 0.855663587 | 1.276964041 | 0.664452307 |
| CITED1 | 1.06332352 | 0.875486112 | 1.291461844 | 0.535840968 |
| NR1D1 | 1.273789173 | 0.996195242 | 1.628735804 | 0.053660303 |
| ZFP36 | 0.79543634 | 0.646763913 | 0.978284284 | 0.030163714 |
| EPO | 0.562295941 | 0.032144158 | 9.836211238 | 0.693360244 |
| SLC34A1 | 8.96481547 | 0.602930739 | 133.2954372 | 0.111253153 |
| MAP1B | 0.767595606 | 0.529711563 | 1.112309143 | 0.162247044 |
| STARD3 | 0.941651205 | 0.54208153 | 1.635744704 | 0.831028713 |
| SERPINF1 | 1.015657191 | 0.823562122 | 1.252558249 | 0.884521812 |
| PCNA | 0.958428188 | 0.558150988 | 1.645763622 | 0.877669678 |
| GSTM3 | 0.907393609 | 0.704524917 | 1.168678554 | 0.451643175 |
| EDNRB | 0.767565864 | 0.631414588 | 0.933075298 | 0.007924626 |
| STXBP1 | 0.82041767 | 0.554172175 | 1.214577678 | 0.322742696 |
| MGARP | 0.63964997 | 0.318952755 | 1.282798401 | 0.208202532 |
| YAP1 | 0.830271331 | 0.599725913 | 1.149442551 | 0.262392598 |
| LHCGR | 0.402085262 | 0.100128659 | 1.614648195 | 0.198971258 |
| CYP1B1 | 0.976223183 | 0.851341697 | 1.119423266 | 0.730413643 |
| MSTN | 1.366671957 | 0.74234111 | 2.516083529 | 0.315785952 |
| SSTR1 | 0.212108799 | 0.076570527 | 0.587564748 | 0.002855391 |
| ESR2 | 0.123389893 | 0.009163576 | 1.661476465 | 0.114736883 |
| CYP11A1 | 0.700856408 | 0.394340806 | 1.245622306 | 0.225733738 |
| PARP1 | 1.105141048 | 0.668832282 | 1.826073248 | 0.696407851 |
| REN | 1.078739528 | 0.848629816 | 1.371244501 | 0.535811509 |
| AGTR1 | 0.559471251 | 0.384476068 | 0.814115903 | 0.002409311 |
| CCNA2 | 2.091325525 | 1.472859163 | 2.969491287 | 3.71E-05 |
| CRHBP | 0.833574696 | 0.631809318 | 1.099772911 | 0.197965436 |
| EGFR | 0.818874953 | 0.629787135 | 1.064734656 | 0.135775286 |
| CYP17A1 | 0.698255586 | 0.38787065 | 1.257019226 | 0.231155306 |
| NR3C2 | 1.038001161 | 0.714292084 | 1.508411522 | 0.844937738 |
| BMP6 | 0.602595877 | 0.407908083 | 0.89020494 | 0.010954519 |
| NODAL | 0.525922151 | 0.091337887 | 3.028251659 | 0.471857809 |
| FBXO32 | 1.177984619 | 0.847293618 | 1.637741312 | 0.329896421 |
| SLC6A1 | 0.767319743 | 0.383547821 | 1.535087817 | 0.454106045 |
| SLC34A2 | 1.040451831 | 0.940967865 | 1.150453754 | 0.439316539 |
| TGFBR2 | 0.680111375 | 0.519144046 | 0.890988707 | 0.005148309 |
| ITGA2 | 1.162079261 | 0.957388664 | 1.410532901 | 0.128643738 |
| GDF9 | 0.630456972 | 0.270925189 | 1.467106088 | 0.284391606 |
| CITED2 | 0.816859171 | 0.643403073 | 1.037077584 | 0.096708905 |
| TNFRSF11B | 0.80049786 | 0.673956689 | 0.950798227 | 0.011255639 |
| GJB2 | 0.922452837 | 0.7121813 | 1.19480705 | 0.540845454 |
| GALR1 | 0.1147013 | 0.00489645 | 2.686923698 | 0.17839354 |
| OR51E2 | 1.293953862 | 0.734453554 | 2.279676624 | 0.372468282 |
| FAM107A | 0.796294639 | 0.630434135 | 1.005791273 | 0.055939626 |
| AR | 0.943770523 | 0.714556208 | 1.246511875 | 0.68350282 |
| TACR3 | 0.545733744 | 0.028693661 | 10.37948119 | 0.686952467 |
| CYP7B1 | 0.750272956 | 0.560833368 | 1.003701884 | 0.052980729 |
| STAT5B | 0.798503291 | 0.498228273 | 1.279749747 | 0.349785965 |
| C1QTNF1 | 0.821027888 | 0.614791622 | 1.096447589 | 0.181513433 |
| NPAS4 | 0.010223121 | 3.10E-05 | 3.368668061 | 0.121290405 |
| LEP | 0.867749824 | 0.303608452 | 2.480134376 | 0.791206898 |
| ZNF366 | 0.632151126 | 0.417934413 | 0.956166885 | 0.029835662 |
| PER1 | 0.709670716 | 0.540273835 | 0.932180113 | 0.013713833 |
| CALR | 0.823598645 | 0.607762608 | 1.116084997 | 0.210698768 |
| NRIP1 | 1.022363984 | 0.826792485 | 1.264196439 | 0.838218963 |
| SSTR2 | 0.774663174 | 0.426335069 | 1.407585434 | 0.40205351 |
| ZNF703 | 0.520886906 | 0.319712764 | 0.848646657 | 0.008820263 |
| SMYD3 | 0.724335092 | 0.476791686 | 1.100399485 | 0.130648939 |
| ZFP36L1 | 1.171295435 | 0.857566888 | 1.599797071 | 0.320231086 |
| PTCH1 | 0.931082568 | 0.619664096 | 1.39900755 | 0.731051035 |
| PPARA | 1.060414666 | 0.686750124 | 1.637392151 | 0.791286686 |
| AKR1C3 | 0.658990743 | 0.459608526 | 0.944866716 | 0.023302776 |
| ESRRG | 0.581357671 | 0.381042047 | 0.886980174 | 0.011856557 |
| MME | 1.021914731 | 0.748461066 | 1.395275941 | 0.891476345 |
| CACNA1H | 0.694428088 | 0.493671124 | 0.976825149 | 0.036202368 |
| ARID5A | 0.729340545 | 0.500130411 | 1.063597851 | 0.101078373 |
| KANK2 | 0.989852366 | 0.668028881 | 1.46671459 | 0.959454901 |
| GPX1 | 0.853494937 | 0.519496919 | 1.402228927 | 0.531720182 |
| TXNIP | 0.878716935 | 0.657436038 | 1.174476917 | 0.382404076 |
| CD24 | 0.70658374 | 0.518206647 | 0.96343917 | 0.028135012 |
| SSTR3 | 0.951802718 | 0.712701009 | 1.271119869 | 0.737877455 |
| PAGR1 | 0.843628412 | 0.462330788 | 1.539393259 | 0.579481672 |
| TFPI | 0.633414081 | 0.396469545 | 1.01196524 | 0.056106525 |
| HOXA11 | 0.758942479 | 0.200007504 | 2.879860378 | 0.685191365 |
| LMO3 | 1.021468138 | 0.778280185 | 1.340644639 | 0.878313267 |
| F7 | 0.855820612 | 0.406434319 | 1.802084336 | 0.681949951 |
| TP63 | 0.95270777 | 0.736992039 | 1.231562957 | 0.711487016 |
| OPRK1 | 0.045476467 | 0.000303866 | 6.80599991 | 0.226488476 |
| APOB | 0.313826753 | 0.009695467 | 10.15806939 | 0.513602435 |
| ESR1 | 0.928016492 | 0.751970761 | 1.145276723 | 0.486382409 |
| SOX10 | 0.136842961 | 0.004694876 | 3.988602538 | 0.24770986 |
| DHRS2 | 1.017378472 | 0.710895131 | 1.455993873 | 0.924946499 |
| RETN | 1.048798196 | 0.862207591 | 1.275768929 | 0.633594498 |
| COL1A1 | 1.072553693 | 0.934384754 | 1.23115389 | 0.31952197 |
| CRYAB | 0.7654068 | 0.610683242 | 0.959331334 | 0.020324429 |
| CCND1 | 0.955671813 | 0.702494598 | 1.300093435 | 0.772783625 |
| BMP5 | 0.381289702 | 0.156623997 | 0.92822198 | 0.0336662 |
| SPP1 | 1.171669896 | 0.981597924 | 1.398546504 | 0.07937926 |
| NR4A3 | 0.85279848 | 0.692977415 | 1.049479004 | 0.132618464 |
| EGR1 | 0.780631088 | 0.6534489 | 0.932567022 | 0.006345164 |
| FLT3 | 1.075453078 | 0.61671864 | 1.875408408 | 0.79765346 |
| PTGFR | 1.082521823 | 0.41053162 | 2.854478046 | 0.872656209 |
| BMP2 | 0.808718066 | 0.621534504 | 1.052274502 | 0.113966983 |
| ALDH1A2 | 1.052010414 | 0.595315234 | 1.859058611 | 0.861442856 |
| FOXA1 | 1.361944976 | 0.913922098 | 2.029597625 | 0.129081598 |
| RARA | 1.164586592 | 0.718776025 | 1.886904798 | 0.536025097 |
| HMGCS2 | 0.525711874 | 0.221024568 | 1.25041744 | 0.145818478 |
| ANXA1 | 0.998238862 | 0.807439647 | 1.23412422 | 0.987005643 |
| AGT | 1.11717411 | 0.802783288 | 1.554688558 | 0.511087513 |
| RXRG | 1.078292925 | 0.929632404 | 1.25072623 | 0.319287387 |
| ADM | 1.144755017 | 0.908144215 | 1.443013156 | 0.25247336 |
| AKR1C2 | 0.71459268 | 0.430911769 | 1.185028433 | 0.192870716 |
| ZFP36L2 | 0.869741459 | 0.711608815 | 1.063014102 | 0.172849272 |
| WNT7A | 1.136818194 | 0.75918743 | 1.70228794 | 0.533605701 |
| MMP14 | 1.010011802 | 0.761489786 | 1.339642184 | 0.944885923 |
| FOXH1 | 1.175685748 | 0.212407924 | 6.507464287 | 0.852921876 |
| UCN | 1.276107126 | 0.944322185 | 1.724463773 | 0.112499078 |
| KRT19 | 1.068499372 | 0.918560431 | 1.242913226 | 0.390433114 |
| AQP4 | 0.951518831 | 0.801828272 | 1.129154604 | 0.56932011 |
| TH | 0.738465646 | 0.346954817 | 1.571765207 | 0.431484026 |
| OXTR | 0.89115239 | 0.491672629 | 1.615206004 | 0.70409641 |
| PENK | 0.984754205 | 0.75890103 | 1.277822544 | 0.907984351 |
| AKR1C1 | 0.516983033 | 0.310425217 | 0.860985004 | 0.011241149 |
| PDGFA | 0.940092051 | 0.577102094 | 1.531398126 | 0.804026738 |
| AQP1 | 0.732122719 | 0.561266739 | 0.954989205 | 0.021469248 |

# Supplementary Table 3. Summary Table of KM Curve Parameter Results.

| **Position** | **HR** | **95% CI** | **C-index** | **P value** |
| --- | --- | --- | --- | --- |
| **Figure 2C** | 0.263 | (0.134, 0.515) | 0.664 | <0.0001 |
| **Supplementary Figure 2B** |  |  |  |  |
| Training set | 10.570 | (3.208, 34.86) | 0.740 | <0.0001 |
| Test set | 7.688 | (1.775, 33.290) | 0.674 | 0.00058 |
| Total set | 9.124 | (3.617, 23.01) | 0.711 | <0.0001 |
| **Figure 5** |  |  |  |  |
| Age<60 | 7.033 | (2.466, 20.060) | 0.704 | <0.0001 |
| Age≥60 | 19.870 | (2.628, 150.300) | 0.729 | <0.0001 |
| Female | 5.085 | (1.956, 13.220) | 0.674 | 0.0002 |
| Male | - | - | 0.780 | <0.0001 |
| No extension | 5.387 | (2.039, 14.230) | 0.692 | 0.00014 |
| With extension | - | - | 0.696 | 0.0002 |
| Stage I+ Stage II | 8.967 | (2.673, 30.080) | 0.732 | <0.0001 |
| Stage III+ Stage Ⅳ | 8.094 | (1.905, 34.400) | 0.658 | 0.00075 |
| T1+T2 | 3.923 | (1.424, 10.800) | 0.674 | 0.0043 |
| T3+T4 | - | - | 0.705 | <0.0001 |
| N0 | 9.962 | (2.264, 43.840) | 0.732 | 0.00017 |
| N1 | 6.127 | (1.858, 20.200) | 0.663 | 0.00067 |
| M0 | 5.949 | (2.29, 15.450) | 0.697 | <0.0001 |
| M1 | - | - | 0.780 | 0.027 |
| Tumor free | 8.829 | (2.657, 29.340) | 0.705 | <0.0001 |
| With tumor | 11.870 | (1.577, 89.320) | 0.683 | 0.0023 |
| **Figure 9D** | 2.239 | (1.230, 4.075) | 0.603 | 0.0067 |
| **Figure 9E** |  |  | 0.741 | <0.0001 |
| Low TMB+High Risk | 10.293 | (2.353, 45.030) |  |  |
| High TMB+Low Risk | 2.767 | (0.462, 16.560) |  |  |
| High TMB+High Risk | 16.753 | (3.976, 70.590) |  |  |

# Supplementary Table 4. Univariate analysis of 838 differential genes.

| **Genes** | **HR** | **lower** | **upper** | **p value** |
| --- | --- | --- | --- | --- |
| HORMAD2 | 8.98172771 | 2.88986458 | 27.9152986 | 0.000148151 |
| LRRC70 | 0.352723587 | 0.199889268 | 0.62241425 | 0.000322763 |
| SCN4B | 0.659963742 | 0.520417816 | 0.836927807 | 0.00060643 |
| RNF223 | 1.860727933 | 1.282374413 | 2.699920092 | 0.001077493 |
| CA4 | 0.781307836 | 0.671338752 | 0.909290478 | 0.001429769 |
| DEPDC1B | 1.379119389 | 1.131210899 | 1.681357818 | 0.001475748 |
| SMOC2 | 0.709402743 | 0.574082251 | 0.876620468 | 0.001475904 |
| PEAR1 | 0.598019805 | 0.435310096 | 0.821546964 | 0.001507996 |
| FHL1 | 0.756704573 | 0.636697711 | 0.89933072 | 0.001554607 |
| TAL1 | 0.522234774 | 0.34663158 | 0.786798362 | 0.00189233 |
| GOLGA7B | 1.613951541 | 1.188617556 | 2.19148671 | 0.002161536 |
| AGTR1 | 0.559471251 | 0.384476068 | 0.814115903 | 0.002409311 |
| VSIG1 | 1.597930115 | 1.180419333 | 2.16311321 | 0.002417618 |
| MMP9 | 1.299021027 | 1.096514363 | 1.538927064 | 0.002482089 |
| BTNL8 | 0.050597542 | 0.007246838 | 0.353272857 | 0.002617809 |
| CASP12 | 0.407802067 | 0.227253262 | 0.731793789 | 0.002641488 |
| EGR2 | 0.764951398 | 0.642029667 | 0.91140748 | 0.00271882 |
| ANGPT1 | 0.605156186 | 0.435740108 | 0.84044136 | 0.002724001 |
| ABCA12 | 2.507669736 | 1.370789364 | 4.587435292 | 0.002850268 |
| IL17C | 4.240824553 | 1.624859153 | 11.06840113 | 0.003160306 |
| TFF3 | 0.833227244 | 0.738117827 | 0.940591889 | 0.003173961 |
| F2RL3 | 0.721210527 | 0.579749462 | 0.897188628 | 0.003347791 |
| GJB6 | 0.652854016 | 0.490557392 | 0.868845058 | 0.003454875 |
| GRIK4 | 0.537270646 | 0.35250415 | 0.818883259 | 0.003861915 |
| RGCC | 0.698713482 | 0.547820382 | 0.891168978 | 0.003874745 |
| ASPHD1 | 1.391955847 | 1.112012878 | 1.742372879 | 0.003892758 |
| PLA2R1 | 0.651342708 | 0.486830104 | 0.871448417 | 0.003897464 |
| SLC14A1 | 0.577917887 | 0.396270576 | 0.842830896 | 0.004397826 |
| LMOD1 | 0.695726691 | 0.541953474 | 0.893131332 | 0.004415702 |
| F3 | 0.75908466 | 0.627403724 | 0.918403093 | 0.004573888 |
| CDKN2B-AS1 | 15.34026449 | 2.297253544 | 102.4369796 | 0.004825095 |
| APOLD1 | 0.751758969 | 0.616388398 | 0.916859483 | 0.004849557 |
| CRABP1 | 0.837908302 | 0.739162552 | 0.949845633 | 0.00570518 |
| ARHGEF37 | 0.568023557 | 0.37924319 | 0.850775359 | 0.006069314 |
| EGR1 | 0.780631088 | 0.6534489 | 0.932567022 | 0.006345164 |
| LIX1 | 0.756341859 | 0.617011099 | 0.927135685 | 0.007182967 |
| RCAN1 | 0.674631324 | 0.505935273 | 0.899576383 | 0.007344677 |
| NMU | 1.16237586 | 1.041142211 | 1.29772631 | 0.007419864 |
| INA | 1.748507913 | 1.159329834 | 2.637109677 | 0.007695974 |
| EMID1 | 0.749472565 | 0.605912485 | 0.927046627 | 0.007855791 |
| EDNRB | 0.767565864 | 0.631414588 | 0.933075298 | 0.007924626 |
| LRP2 | 0.788400842 | 0.6608891 | 0.940514661 | 0.008258987 |
| HID1-AS1 | 0.514753977 | 0.314311731 | 0.843021849 | 0.008329174 |
| DGKI | 0.689164786 | 0.522485925 | 0.90901607 | 0.008408331 |
| GDF7 | 0.202488229 | 0.061475137 | 0.666960413 | 0.008641908 |
| GNA14 | 0.727455659 | 0.57339372 | 0.922911635 | 0.008776074 |
| BDKRB2 | 0.656416616 | 0.478016512 | 0.901397259 | 0.009281799 |
| AADACP1 | 0.520124998 | 0.317101048 | 0.853135035 | 0.009623362 |
| FLT1 | 0.743831553 | 0.594338427 | 0.930926479 | 0.009731893 |
| EGOT | 1.648541902 | 1.128267255 | 2.408729307 | 0.009774031 |
| THSD7B | 0.475029017 | 0.26929866 | 0.83792681 | 0.010152238 |
| C2CD4A | 1.228670802 | 1.049590387 | 1.438305799 | 0.010403004 |
| CHRM3 | 0.54860548 | 0.346513784 | 0.868559885 | 0.010434163 |
| SYT1 | 1.305247347 | 1.062756305 | 1.603068013 | 0.011072622 |
| TNFRSF11B | 0.80049786 | 0.673956689 | 0.950798227 | 0.011255639 |
| KCNJ13 | 0.57200918 | 0.37088692 | 0.882194771 | 0.011504616 |
| FCRLB | 1.38590293 | 1.075545347 | 1.785816782 | 0.011636262 |
| ESRRG | 0.581357671 | 0.381042047 | 0.886980174 | 0.011856557 |
| PLA2G2E | 1.372866911 | 1.072586213 | 1.757214043 | 0.011856621 |
| SLC10A4 | 0.48816647 | 0.279179124 | 0.853597143 | 0.011897283 |
| FHL5 | 0.613148341 | 0.417798994 | 0.899836748 | 0.012447042 |
| C8orf88 | 0.568144869 | 0.363396336 | 0.888254944 | 0.013150289 |
| LINC01010 | 3.191415911 | 1.273645595 | 7.996836447 | 0.013283864 |
| VTCN1 | 1.190035193 | 1.036657012 | 1.366106382 | 0.013460683 |
| MUC21 | 1.193283007 | 1.036348514 | 1.373982126 | 0.014039871 |
| RGS8 | 0.605747425 | 0.405916542 | 0.90395415 | 0.014113936 |
| SLC5A5 | 0.689900809 | 0.51208735 | 0.929456912 | 0.014645731 |
| TPO | 0.883538595 | 0.79983244 | 0.976004985 | 0.014759513 |
| DIRAS2 | 0.592151721 | 0.38822498 | 0.903197061 | 0.014989251 |
| MT-TP | 1.381541609 | 1.064749824 | 1.792587491 | 0.015012314 |
| SORBS2 | 0.733818471 | 0.571685478 | 0.941933231 | 0.015116916 |
| AQP9 | 1.475657016 | 1.074778318 | 2.026058389 | 0.016134783 |
| MPPED2 | 0.75431465 | 0.599143804 | 0.94967283 | 0.016421476 |
| NPR3 | 0.772677263 | 0.625303086 | 0.954785233 | 0.016918101 |
| TMC3 | 0.459246269 | 0.241805589 | 0.872217789 | 0.01742093 |
| SCN3A | 0.293194319 | 0.106299083 | 0.808689093 | 0.0177802 |
| FABP4 | 0.836488862 | 0.72162593 | 0.969634802 | 0.017829788 |
| KDR | 0.773962458 | 0.625278487 | 0.958001753 | 0.018564334 |
| QRFPR | 0.624494392 | 0.421917751 | 0.924334767 | 0.018611157 |
| BANCR | 0.449724839 | 0.231104205 | 0.875156865 | 0.018645598 |
| MT-CO3 | 1.510214304 | 1.066273231 | 2.138989499 | 0.020271345 |
| AKR1E2 | 0.50161393 | 0.280046981 | 0.898479726 | 0.020344501 |
| CDH16 | 0.809795012 | 0.677197275 | 0.968355879 | 0.020755108 |
| GLT8D2 | 0.741692547 | 0.575340678 | 0.956142778 | 0.021106914 |
| FOS | 0.837162791 | 0.719453554 | 0.974130344 | 0.021505687 |
| FOSB | 0.865241612 | 0.764668407 | 0.979042733 | 0.021680871 |
| CERS1 | 1.566088134 | 1.067081119 | 2.29844948 | 0.021925555 |
| ANGPTL1 | 0.784388267 | 0.637100787 | 0.965726251 | 0.022101208 |
| CDKN2A | 1.306250859 | 1.038952318 | 1.642319168 | 0.022190363 |
| PRTN3 | 1.561419214 | 1.065158871 | 2.288888566 | 0.022404396 |
| FNDC1 | 0.83521752 | 0.715494575 | 0.974973578 | 0.022546535 |
| UBTFL6 | 0.490822478 | 0.265594995 | 0.907045351 | 0.023126352 |
| BTNL9 | 0.776640175 | 0.624340142 | 0.966091911 | 0.023225299 |
| NOX5 | 0.602219185 | 0.388202179 | 0.934224398 | 0.023594786 |
| TFCP2L1 | 0.784224227 | 0.635158242 | 0.968274671 | 0.023840574 |
| PDE10A | 0.613129792 | 0.400930657 | 0.937638804 | 0.024004305 |
| DUSP13 | 1.369016486 | 1.041553447 | 1.799433477 | 0.02433152 |
| PLXNA4 | 0.75357755 | 0.587810131 | 0.966092781 | 0.025607408 |
| FCGBP | 0.771325991 | 0.614064872 | 0.968861454 | 0.025622572 |
| CRABP2 | 1.244174656 | 1.026021414 | 1.508711761 | 0.026342088 |
| SLC5A1 | 1.390967573 | 1.038664705 | 1.862767437 | 0.02679149 |
| MT-CYB | 1.464640472 | 1.044174681 | 2.054418433 | 0.027081581 |
| IGDCC4 | 0.746632197 | 0.576082973 | 0.967672479 | 0.027221002 |
| TG | 0.851823754 | 0.73865223 | 0.982334688 | 0.027452931 |
| PRKG1 | 0.685415987 | 0.489900839 | 0.958959524 | 0.027486218 |
| AVPR1A | 0.745265709 | 0.573151765 | 0.96906441 | 0.028198548 |
| WNT11 | 0.670208744 | 0.468010976 | 0.959763303 | 0.028953618 |
| AHNAK2 | 1.227247285 | 1.019727093 | 1.476999001 | 0.030260839 |
| SLC5A4 | 0.550865365 | 0.319732616 | 0.949082562 | 0.031694177 |
| SLITRK2 | 0.006575682 | 6.56E-05 | 0.659462521 | 0.03259443 |
| SERTM1 | 0.75536378 | 0.582246349 | 0.979953659 | 0.0346491 |
| MT-ND4 | 1.434984292 | 1.025905686 | 2.007182478 | 0.03491515 |
| HLF | 0.802232459 | 0.653407502 | 0.984954896 | 0.035312134 |
| PDZK1IP1 | 1.160462827 | 1.009989031 | 1.333355049 | 0.03570802 |
| FAM170B-AS1 | 0.415359289 | 0.182785549 | 0.943856557 | 0.035911099 |
| OCA2 | 0.746969109 | 0.568290158 | 0.981827403 | 0.03648838 |
| MT-CO2 | 1.504999911 | 1.025185793 | 2.209379752 | 0.036892767 |
| IL4I1 | 1.243660525 | 1.013100724 | 1.526690747 | 0.037125827 |
| ARMC4P1 | 0.597540373 | 0.367318701 | 0.972056409 | 0.038068014 |
| HOXD9 | 1.302512102 | 1.014273093 | 1.672663692 | 0.038357164 |
| CD52 | 1.24770993 | 1.011353002 | 1.539304344 | 0.03889311 |
| MT-ND4L | 1.330064833 | 1.014130533 | 1.744422834 | 0.039267553 |
| FAM167A | 0.853469371 | 0.733776404 | 0.99268655 | 0.03986218 |
| PLP1 | 1.197848485 | 1.007431614 | 1.424256468 | 0.040974504 |
| PKHD1L1 | 0.838536406 | 0.707881332 | 0.99330675 | 0.041582553 |
| ODF3L1 | 0.730491037 | 0.539569179 | 0.988968933 | 0.042181197 |
| PSG1 | 1.23090781 | 1.007322085 | 1.504120737 | 0.042225093 |
| IRS1 | 0.781498399 | 0.615507162 | 0.992254494 | 0.042991551 |
| LINC01257 | 0.426038251 | 0.185071334 | 0.980749354 | 0.044892831 |
| TNFSF18 | 1.327424483 | 1.006397329 | 1.750854961 | 0.044951556 |
| PDE7B | 0.779943324 | 0.61149832 | 0.99478865 | 0.045278975 |
| MT-ND3 | 1.407841723 | 1.005084381 | 1.971991959 | 0.046650387 |
| ALDH3B2 | 1.483201892 | 1.005832112 | 2.187132254 | 0.046667029 |
| RERGL | 0.712399887 | 0.509115922 | 0.996852735 | 0.047888827 |
| SLC26A4-AS1 | 0.895156762 | 0.801744631 | 0.999452442 | 0.048872248 |
| SUSD5 | 0.752405298 | 0.566899974 | 0.998613086 | 0.048887349 |
| NPY5R | 0.168333886 | 0.028117697 | 1.007774488 | 0.050999726 |
| GNRH2 | 0.507192632 | 0.256264468 | 1.003823775 | 0.051294597 |
| RAG1 | 0.557542842 | 0.308226715 | 1.00852394 | 0.053372305 |
| SH2D6 | 0.597219652 | 0.353686914 | 1.008437965 | 0.053789547 |
| SH3RF3-AS1 | 0.560235921 | 0.310813717 | 1.009814786 | 0.053920776 |
| CATIP-AS2 | 0.614262894 | 0.373780018 | 1.009467827 | 0.054507167 |
| LINC00415 | 0.584388679 | 0.337522513 | 1.011814368 | 0.055107444 |
| SLC28A3 | 1.346595396 | 0.992749214 | 1.826563179 | 0.055725282 |
| KCNAB1 | 0.850703777 | 0.720556928 | 1.004357724 | 0.056309027 |
| PIGR | 1.270640665 | 0.993379758 | 1.625287496 | 0.056510477 |
| TRAV13-1 | 1.298572773 | 0.992359481 | 1.699274588 | 0.056901436 |
| NR4A1 | 0.849458403 | 0.717881266 | 1.005151706 | 0.057417223 |
| NWD1 | 0.607410125 | 0.363091934 | 1.016125738 | 0.0575606 |
| PDK4 | 0.861604972 | 0.738676179 | 1.004991294 | 0.057884478 |
| ATP2C2 | 0.697184218 | 0.47979463 | 1.013070599 | 0.058510366 |
| CAPN6 | 0.755094903 | 0.564393838 | 1.010231287 | 0.058567622 |
| TGFBR3L | 1.657181683 | 0.980664103 | 2.800399365 | 0.059157443 |
| CSPG4P13 | 0.337043983 | 0.108444746 | 1.047525588 | 0.060147236 |
| UTS2R | 0.719075895 | 0.509811203 | 1.014238485 | 0.060190699 |
| KIT | 0.819570051 | 0.66563503 | 1.009104146 | 0.060850473 |
| HGD | 0.849829867 | 0.71687042 | 1.007449579 | 0.060866281 |
| SDR16C5 | 1.269890152 | 0.986944449 | 1.633953157 | 0.063199811 |
| FOXA2 | 0.81859287 | 0.662674987 | 1.011195985 | 0.06335512 |
| ARSI | 1.26777467 | 0.986239745 | 1.62967739 | 0.064051603 |
| ABCC11 | 0.712549344 | 0.496953654 | 1.021677904 | 0.065282707 |
| SLC5A8 | 0.875828809 | 0.760493253 | 1.008656027 | 0.065719563 |
| FAM189A1 | 0.776703318 | 0.593073299 | 1.017189689 | 0.066339442 |
| ATP10B | 1.216747757 | 0.986848379 | 1.500205233 | 0.066348561 |
| LINC00973 | 1.272922629 | 0.983843 | 1.646941656 | 0.066353105 |
| STC1 | 0.804923251 | 0.638072227 | 1.015404545 | 0.067103382 |
| RENBP | 1.28262367 | 0.982539684 | 1.674358303 | 0.067185767 |
| ETV7 | 1.272110366 | 0.982548327 | 1.647007826 | 0.067795469 |
| ATF3 | 0.866703037 | 0.743346538 | 1.010530239 | 0.067813916 |
| TCF7L1 | 0.800669117 | 0.630470461 | 1.016813752 | 0.06827077 |
| PROSER2-AS1 | 0.763925663 | 0.571813122 | 1.020582418 | 0.068438667 |
| MT-ND2 | 1.346669129 | 0.977550163 | 1.855165917 | 0.068600493 |
| CEACAM1 | 1.307357583 | 0.979270729 | 1.745363974 | 0.069082339 |
| PROX1 | 0.751935691 | 0.552056059 | 1.024184545 | 0.070545917 |
| FOXF2 | 0.700050554 | 0.474292463 | 1.033267059 | 0.07261933 |
| SLC6A14 | 1.195128137 | 0.983666011 | 1.452049017 | 0.072781606 |
| FOXP2 | 0.69016677 | 0.459718592 | 1.036134235 | 0.073656997 |
| CRLF2 | 1.187406246 | 0.983483903 | 1.433611257 | 0.073980786 |
| SLC26A4 | 0.894618594 | 0.791667207 | 1.010958166 | 0.074221955 |
| BMX | 0.702920536 | 0.476949414 | 1.035953217 | 0.074837338 |
| GDF10 | 0.769885678 | 0.577047199 | 1.027167204 | 0.07544534 |
| FAM111B | 1.275633889 | 0.973872236 | 1.670898666 | 0.077107832 |
| MMP12 | 1.205205973 | 0.979596501 | 1.482775241 | 0.077559318 |
| SNCG | 1.214856733 | 0.978690711 | 1.508011537 | 0.077620011 |
| AC078899.4 | 0.819440511 | 0.656735679 | 1.022455111 | 0.077845871 |
| SLC7A10 | 0.601162734 | 0.340123833 | 1.062544281 | 0.079911767 |
| BIRC7 | 1.128383716 | 0.985670234 | 1.291760434 | 0.079987898 |
| TMEM215 | 0.879270848 | 0.761060941 | 1.015841416 | 0.080706566 |
| TMEM252 | 0.473010855 | 0.204171904 | 1.095837697 | 0.080730527 |
| MMRN1 | 0.770596493 | 0.575102768 | 1.032544076 | 0.080905993 |
| PKNOX2 | 0.85487297 | 0.715953786 | 1.02074716 | 0.083094098 |
| GPNMB | 1.16586141 | 0.98006355 | 1.386882337 | 0.083166311 |
| RAB27B | 1.182570319 | 0.978164065 | 1.429691203 | 0.083284368 |
| ERBB4 | 0.726984262 | 0.506549857 | 1.043344715 | 0.083671127 |
| S100A1 | 1.33932753 | 0.961520956 | 1.865584126 | 0.084005798 |
| ZBTB16 | 0.792991888 | 0.608823453 | 1.032871073 | 0.085412558 |
| AGR3 | 0.756000307 | 0.549196216 | 1.040678081 | 0.086265863 |
| TBX2-AS1 | 0.788927654 | 0.60163819 | 1.034520171 | 0.086431149 |
| MTATP6P1 | 1.328981719 | 0.959678544 | 1.840400016 | 0.086860689 |
| IGHV3-49 | 1.082020626 | 0.988639333 | 1.184222189 | 0.086924696 |
| SPATA12 | 2.154802197 | 0.894280158 | 5.192078193 | 0.087091864 |
| EPHB3 | 1.219139773 | 0.971411663 | 1.530043175 | 0.087322232 |
| LRP1B | 0.703630707 | 0.469791141 | 1.053864428 | 0.088116221 |
| TRPV6 | 0.659757413 | 0.408811387 | 1.064744912 | 0.088556794 |
| HNF1B | 0.380426899 | 0.124927754 | 1.158466557 | 0.088931804 |
| SLC29A4 | 0.776734947 | 0.580061137 | 1.040092395 | 0.089870887 |
| USH1C | 0.555516833 | 0.28127704 | 1.097135235 | 0.090457921 |
| TMC3-AS1 | 0.212611857 | 0.035106896 | 1.287604622 | 0.092011842 |
| ABCA8 | 0.790795578 | 0.60094038 | 1.040631762 | 0.093810194 |
| HK3 | 1.258354834 | 0.96083889 | 1.647994168 | 0.09497719 |
| RXFP1 | 0.515632133 | 0.236814884 | 1.122718692 | 0.095236792 |
| GCGR | 1.185040614 | 0.970473644 | 1.447047292 | 0.095736755 |
| KIF19 | 0.690221135 | 0.44614619 | 1.067823119 | 0.095869286 |
| GPM6A | 0.766667123 | 0.560144247 | 1.049334134 | 0.097066736 |
| S100A4 | 1.163238327 | 0.972638571 | 1.391188304 | 0.09769945 |
| SLC16A2 | 0.825004696 | 0.656918272 | 1.036099584 | 0.097948483 |
| EFHC2 | 0.749374784 | 0.532261086 | 1.055050955 | 0.098341982 |
| FRMD5 | 1.182545936 | 0.968697234 | 1.443603679 | 0.099460444 |
| CDK2AP2P2 | 0.653058308 | 0.393285103 | 1.084417259 | 0.099609509 |
| HLA-DQB1 | 1.177839087 | 0.967080414 | 1.434529017 | 0.103695693 |
| GYPE | 0.625347231 | 0.355110699 | 1.101231702 | 0.103955376 |
| APOBEC3A | 1.362465762 | 0.937421075 | 1.980233859 | 0.104967847 |
| KCNE3 | 0.818378816 | 0.642196607 | 1.042895398 | 0.10514588 |
| WSCD2 | 0.871425883 | 0.737337179 | 1.029899335 | 0.106445731 |
| CFB | 1.385428996 | 0.930148905 | 2.063555084 | 0.108768741 |
| BTBD11 | 0.833943211 | 0.667905056 | 1.041257695 | 0.108921822 |
| SCGN | 0.698042659 | 0.449245225 | 1.084627119 | 0.109890816 |
| C15orf48 | 1.182937805 | 0.961883416 | 1.454793615 | 0.111439049 |
| AMBP | 0.393030441 | 0.124131887 | 1.244425836 | 0.112264634 |
| STYK1 | 1.645842984 | 0.889403248 | 3.04563665 | 0.112576434 |
| SLC25A15 | 0.798278356 | 0.604067847 | 1.054928412 | 0.113191419 |
| IVL | 1.124818242 | 0.972358328 | 1.30118295 | 0.113474443 |
| ADIG | 0.77232359 | 0.560697708 | 1.063824088 | 0.113814195 |
| MT-ATP8 | 1.176279651 | 0.96161524 | 1.438864279 | 0.11428075 |
| MT-ND1 | 1.271607726 | 0.943076869 | 1.714585802 | 0.115107366 |
| MT-ATP6 | 1.327305243 | 0.932383267 | 1.889501099 | 0.11608556 |
| NFE2L3 | 1.163585246 | 0.962465915 | 1.406730985 | 0.117622727 |
| LTA | 1.310019345 | 0.933454356 | 1.838494484 | 0.118355699 |
| LINC01484 | 1.797528509 | 0.860383611 | 3.755428044 | 0.118774014 |
| SEMA3D | 0.870103235 | 0.729833481 | 1.037332021 | 0.120823576 |
| H19 | 1.156818777 | 0.962001952 | 1.391088323 | 0.121563949 |
| FFAR4 | 1.511585506 | 0.892803102 | 2.559232532 | 0.124073854 |
| TMEM40 | 1.313578719 | 0.926736538 | 1.861898158 | 0.12540524 |
| TINCR | 1.168531254 | 0.957081219 | 1.426697406 | 0.126203679 |
| SAMD5 | 0.734165594 | 0.493823117 | 1.091482153 | 0.126681699 |
| EGR3 | 0.851429691 | 0.692138889 | 1.047380128 | 0.12802712 |
| ELMO1 | 0.834410506 | 0.66005938 | 1.054815541 | 0.130094263 |
| CD36 | 0.858914276 | 0.705114719 | 1.046260578 | 0.130852923 |
| CTNNA3 | 0.027106967 | 0.000250062 | 2.938422394 | 0.131268684 |
| CNTFR | 1.108592087 | 0.969639392 | 1.267457187 | 0.13136327 |
| DMBX1 | 1.161341593 | 0.956100822 | 1.410640242 | 0.131678399 |
| IGDCC3 | 0.780544006 | 0.565571527 | 1.077227046 | 0.131713436 |
| NR4A3 | 0.85279848 | 0.692977415 | 1.049479004 | 0.132618464 |
| FAM178B | 1.253098387 | 0.93367607 | 1.681799093 | 0.132878147 |
| C1QL2 | 1.149889269 | 0.958212817 | 1.37990779 | 0.133311952 |
| MT1F | 0.890980508 | 0.766262094 | 1.03599835 | 0.133534423 |
| SHANK2 | 0.770445022 | 0.547975651 | 1.083233409 | 0.133593938 |
| FN1 | 1.092149953 | 0.973261297 | 1.225561445 | 0.133859644 |
| TMPRSS6 | 1.093425254 | 0.972813904 | 1.228990234 | 0.134195906 |
| HLA-DQA2 | 1.120408195 | 0.965357352 | 1.300362524 | 0.134644694 |
| CACNA2D2 | 0.822798796 | 0.637158838 | 1.062526044 | 0.134896287 |
| AC008268.1 | 0.371795347 | 0.101535299 | 1.36141599 | 0.135156814 |
| CHRDL1 | 0.864649615 | 0.714182803 | 1.046817361 | 0.135986295 |
| ABHD11-AS1 | 1.170903741 | 0.951351403 | 1.441124244 | 0.13642691 |
| MYOC | 0.815076078 | 0.622409313 | 1.067382829 | 0.137267729 |
| BARX2 | 0.548447998 | 0.247070856 | 1.217445114 | 0.139846446 |
| DAW1 | 1.46122244 | 0.879402515 | 2.427979205 | 0.143213566 |
| HOXD-AS2 | 1.923592924 | 0.797732916 | 4.638406741 | 0.145184931 |
| KCNK17 | 0.850922944 | 0.684420786 | 1.057930838 | 0.146204074 |
| LOX | 1.157514393 | 0.950149275 | 1.410135867 | 0.146427426 |
| DUOX2 | 0.886549268 | 0.753529331 | 1.04305111 | 0.146559351 |
| TMPRSS11E | 1.129836867 | 0.958031795 | 1.332451963 | 0.146914515 |
| SCARNA7 | 1.266434875 | 0.918746874 | 1.745700952 | 0.14917556 |
| PRIMA1 | 0.726026286 | 0.469852515 | 1.121871547 | 0.149296882 |
| MT-RNR1 | 1.230471872 | 0.92770544 | 1.632049315 | 0.150087601 |
| ZSWIM5P2 | 0.373580348 | 0.097671181 | 1.428899244 | 0.150284198 |
| TNNT1 | 1.125775934 | 0.957533618 | 1.323579069 | 0.151423103 |
| IL1RN | 1.169653186 | 0.944179955 | 1.448970154 | 0.151498216 |
| ST6GALNAC5 | 1.119709912 | 0.959343742 | 1.306883271 | 0.151662376 |
| FAM83A-AS1 | 1.611126617 | 0.839296721 | 3.092742903 | 0.151735953 |
| KRT89P | 0.603275741 | 0.301680914 | 1.20637933 | 0.152910955 |
| JAKMIP2 | 1.977358162 | 0.776253889 | 5.036941335 | 0.152985784 |
| MIOX | 0.857538945 | 0.694415036 | 1.058982027 | 0.153399811 |
| KHDRBS2 | 0.835398207 | 0.65234213 | 1.069822311 | 0.154116729 |
| MYO1G | 1.132692081 | 0.953738336 | 1.345223636 | 0.155576584 |
| CCR9 | 1.429817702 | 0.870604912 | 2.348227803 | 0.157792007 |
| FAM83A | 1.156034532 | 0.945016093 | 1.414172572 | 0.158536464 |
| IGHE | 0.810519127 | 0.605143558 | 1.085595719 | 0.158807746 |
| SYNE1 | 0.810802008 | 0.605471928 | 1.085764452 | 0.159224508 |
| HSD3B7 | 1.338230276 | 0.890994245 | 2.009957171 | 0.160367816 |
| PLEKHG4B | 0.792900146 | 0.573258253 | 1.096697061 | 0.160849703 |
| S100A6 | 1.200898807 | 0.927011294 | 1.555706985 | 0.165709796 |
| TRAPPC3L | 0.677980645 | 0.390693825 | 1.176516559 | 0.166992446 |
| GOLGA8K | 0.242133193 | 0.032387483 | 1.810220436 | 0.16703865 |
| LRRN2 | 0.834313261 | 0.64505018 | 1.079107702 | 0.16759533 |
| AC141930.2 | 0.845253156 | 0.665520258 | 1.073525395 | 0.168109125 |
| IL36RN | 1.282622709 | 0.900022447 | 1.827866648 | 0.168462755 |
| CNTN2 | 3.160615229 | 0.609895822 | 16.37900812 | 0.17040419 |
| HLA-A | 1.270391084 | 0.902097939 | 1.789044665 | 0.170651422 |
| LAG3 | 1.187820106 | 0.92788833 | 1.520567247 | 0.171943789 |
| TRIM46 | 1.281341652 | 0.897472525 | 1.829400214 | 0.172394042 |
| AKR1B15 | 0.494232739 | 0.17959794 | 1.36007128 | 0.172404628 |
| TPTE2P1 | 0.883778977 | 0.739955894 | 1.055556537 | 0.172777881 |
| CCL24 | 1.239842573 | 0.909623674 | 1.689940192 | 0.1736687 |
| DHDH | 1.196649803 | 0.923255462 | 1.551001655 | 0.174913438 |
| NGEF | 1.131044731 | 0.946624249 | 1.351393847 | 0.175112664 |
| MYH15 | 0.176335101 | 0.014238149 | 2.18385606 | 0.176501795 |
| GALR1 | 0.1147013 | 0.00489645 | 2.686923698 | 0.17839354 |
| MUC1 | 1.11325194 | 0.951881672 | 1.301978931 | 0.17935098 |
| ZMAT4 | 0.882962864 | 0.735837454 | 1.059504942 | 0.180756217 |
| CPXM1 | 0.900797345 | 0.772680523 | 1.050156995 | 0.181964617 |
| COL25A1 | 0.772989332 | 0.529368095 | 1.128727841 | 0.182512375 |
| AOX1 | 0.83477083 | 0.639839629 | 1.089089058 | 0.183189048 |
| FOXJ1 | 0.864633524 | 0.697813014 | 1.071334462 | 0.183542444 |
| MTND6P3 | 0.671633561 | 0.373408714 | 1.208037262 | 0.183862881 |
| PYGM | 0.837820987 | 0.645291523 | 1.087793627 | 0.18408523 |
| FLRT1 | 0.833827776 | 0.637459828 | 1.090686394 | 0.184712816 |
| DNAI2 | 0.187984721 | 0.015798126 | 2.236863761 | 0.185901962 |
| KCTD16 | 0.522503167 | 0.199373439 | 1.36933767 | 0.18666033 |
| LCN2 | 1.086246579 | 0.960440399 | 1.228531861 | 0.187749455 |
| CD177 | 1.087327304 | 0.959462921 | 1.232231741 | 0.189637199 |
| LINC00113 | 1.265078598 | 0.888221646 | 1.801829381 | 0.192550651 |
| GLP1R | 0.861040181 | 0.687463596 | 1.078442842 | 0.192740286 |
| AKR1C2 | 0.71459268 | 0.430911769 | 1.185028433 | 0.192870716 |
| SLC26A7 | 0.917815624 | 0.806520873 | 1.044468343 | 0.193502096 |
| GPR158 | 1.364401118 | 0.85320173 | 2.181887759 | 0.194570745 |
| HLA-DMA | 1.196943678 | 0.910237205 | 1.573956944 | 0.19817418 |
| LHCGR | 0.402085262 | 0.100128659 | 1.614648195 | 0.198971258 |
| IGHV4-39 | 1.052204439 | 0.97357894 | 1.137179674 | 0.19906442 |
| KIF5C | 0.678167568 | 0.373402948 | 1.231675466 | 0.202110871 |
| CD74 | 1.172679334 | 0.917729327 | 1.498455786 | 0.20282024 |
| SMAD9-IT1 | 0.756865195 | 0.492073242 | 1.164145649 | 0.204764462 |
| PRSS21 | 1.459928112 | 0.812748294 | 2.622447946 | 0.205450454 |
| KCNK2 | 0.858692741 | 0.677663632 | 1.088081445 | 0.207256397 |
| COL9A3 | 0.906615354 | 0.777989207 | 1.056507459 | 0.209177736 |
| MTND4P12 | 1.076623073 | 0.959265941 | 1.208337742 | 0.209933805 |
| CWH43 | 0.881508693 | 0.723575954 | 1.073912935 | 0.210551526 |
| SERPIND1 | 0.684884661 | 0.378419473 | 1.239542446 | 0.211116783 |
| CECR2 | 0.663189273 | 0.347622747 | 1.265222185 | 0.212706066 |
| MT1G | 0.944951183 | 0.864116584 | 1.03334753 | 0.214605886 |
| KCNB1 | 1.433952391 | 0.810623285 | 2.536590666 | 0.215520855 |
| SLC25A47P1 | 1.087017712 | 0.952351763 | 1.240725908 | 0.216280123 |
| EREG | 1.159727879 | 0.916784129 | 1.467050652 | 0.216627498 |
| TRAV8-6 | 1.241570246 | 0.880091563 | 1.751518525 | 0.217784055 |
| CCL22 | 1.136464325 | 0.927248798 | 1.392885238 | 0.217828864 |
| RELN | 0.411371875 | 0.09993891 | 1.693302641 | 0.218543997 |
| GRIN2C | 0.892403653 | 0.744206596 | 1.070111827 | 0.219217131 |
| MCEMP1 | 1.143113427 | 0.92318949 | 1.415428058 | 0.219866099 |
| ZNF385B | 1.539073388 | 0.772791342 | 3.065183011 | 0.21993979 |
| TDRD6 | 0.764494624 | 0.497769693 | 1.174141452 | 0.219952951 |
| SOSTDC1 | 0.790838516 | 0.542940685 | 1.151922442 | 0.221364408 |
| SLC4A4 | 0.89420962 | 0.747404272 | 1.069850514 | 0.221692129 |
| CYP4F32P | 0.895068258 | 0.749259878 | 1.069251417 | 0.221742569 |
| FOXP3 | 1.193670785 | 0.897997566 | 1.586696888 | 0.222810072 |
| ANKRD36BP2 | 1.112806108 | 0.937035308 | 1.321548315 | 0.223018663 |
| DCSTAMP | 1.057638018 | 0.965322952 | 1.158781292 | 0.229137831 |
| IRX6 | 0.835251006 | 0.622509473 | 1.12069659 | 0.230046276 |
| MEIS3P2 | 0.8525993 | 0.65675755 | 1.106840059 | 0.231067621 |
| ITPR1 | 0.857761101 | 0.667167152 | 1.102803254 | 0.231417561 |
| BEAN1 | 1.205159655 | 0.887006018 | 1.637429469 | 0.232767385 |
| IGFL4 | 1.607597426 | 0.735575976 | 3.513395718 | 0.234004879 |
| OLR1 | 1.150060161 | 0.913406093 | 1.448028849 | 0.234271594 |
| SFTA2 | 1.150708595 | 0.911584168 | 1.45255953 | 0.237564216 |
| PI16 | 0.899376902 | 0.753941517 | 1.072866786 | 0.238625142 |
| LRRC37A11P | 0.299383032 | 0.039769472 | 2.253743798 | 0.24160489 |
| IGHV1-46 | 1.054339379 | 0.964851493 | 1.152127074 | 0.242288767 |
| LINC01539 | 0.629034063 | 0.288841424 | 1.369899951 | 0.243057179 |
| HLA-DPB1 | 1.143153709 | 0.912594478 | 1.431961767 | 0.244371843 |
| SH2D2A | 1.204012331 | 0.880659701 | 1.646090642 | 0.244615092 |
| LIPG | 0.894676438 | 0.741669942 | 1.079248172 | 0.244827711 |
| LINC01586 | 0.832588211 | 0.611251132 | 1.13407255 | 0.245232031 |
| IGLV1-40 | 1.047367761 | 0.968562203 | 1.132585211 | 0.24620995 |
| ADCY8 | 1.092694614 | 0.940499621 | 1.269518343 | 0.246714085 |
| CCL20 | 1.120952261 | 0.923457244 | 1.360684513 | 0.248225516 |
| SLC6A20 | 1.10805304 | 0.930579685 | 1.319372816 | 0.249280807 |
| GABRG1 | 0.610408803 | 0.262815614 | 1.417719825 | 0.250920733 |
| TNN | 0.814283035 | 0.573160813 | 1.156842628 | 0.25148789 |
| HLA-DOA | 1.105167536 | 0.931256903 | 1.311555682 | 0.252337664 |
| FRMPD3 | 0.854562971 | 0.652840575 | 1.118615936 | 0.252611884 |
| GP5 | 0.586377081 | 0.234634232 | 1.465421641 | 0.253356241 |
| IGLV2-14 | 1.045315415 | 0.968659153 | 1.128037982 | 0.254071453 |
| CEACAM6 | 1.078655314 | 0.946967287 | 1.22865626 | 0.254400571 |
| IGHA2 | 1.055703166 | 0.961710278 | 1.158882462 | 0.254554888 |
| VSTM2L | 1.123070921 | 0.919725457 | 1.371374777 | 0.254755184 |
| CYP26B1 | 0.859363331 | 0.660671792 | 1.117809697 | 0.258567846 |
| GOLGA8O | 0.582547638 | 0.227310773 | 1.492941778 | 0.260442292 |
| NME8 | 1.194263001 | 0.876388928 | 1.627432834 | 0.260873853 |
| KY | 0.835439696 | 0.610448907 | 1.143354469 | 0.261384203 |
| MT-CO1 | 1.224843969 | 0.859730702 | 1.745014741 | 0.261411395 |
| CST5 | 0.896137107 | 0.739834876 | 1.085460744 | 0.262120776 |
| TNFRSF18 | 1.132270352 | 0.911212571 | 1.40695617 | 0.262306331 |
| PDCD1LG2 | 1.191277106 | 0.876423215 | 1.619241844 | 0.263713938 |
| EYA4 | 0.757022032 | 0.464537812 | 1.23366138 | 0.26391114 |
| SRPX2 | 1.182325846 | 0.881130589 | 1.586478128 | 0.264246145 |
| COL11A1 | 1.091748683 | 0.935171337 | 1.274542043 | 0.266409037 |
| HCP5 | 1.175072347 | 0.883758697 | 1.562411804 | 0.267058936 |
| FER1L6 | 0.740631724 | 0.435719164 | 1.258919498 | 0.267306623 |
| NRSN1 | 1.204687596 | 0.866409585 | 1.675041724 | 0.268165229 |
| EBF4 | 0.896951144 | 0.739780711 | 1.087513289 | 0.268534549 |
| CAPN8 | 1.216902102 | 0.85759597 | 1.726746368 | 0.271539214 |
| IGHA1 | 1.046098771 | 0.96525985 | 1.133707819 | 0.272075323 |
| GOLGA8R | 0.698173531 | 0.367201037 | 1.327464332 | 0.2731149 |
| BEND6 | 1.198734005 | 0.866109169 | 1.659101723 | 0.274341714 |
| AP004782.1 | 0.455320083 | 0.110866482 | 1.869964424 | 0.275028953 |
| IGHD | 1.068588327 | 0.948546319 | 1.20382209 | 0.275221085 |
| FUT3 | 1.155130385 | 0.891328011 | 1.497009172 | 0.275604959 |
| FAM181A-AS1 | 0.642949392 | 0.290520727 | 1.422906811 | 0.275819204 |
| MTRNR2L6 | 0.454149931 | 0.109577674 | 1.882246196 | 0.276550818 |
| DNAAF1 | 0.778227413 | 0.495225592 | 1.222953569 | 0.276934508 |
| EBI3 | 1.140461111 | 0.899712865 | 1.445629596 | 0.277293634 |
| TSPAN18 | 0.888082188 | 0.716814615 | 1.100270497 | 0.277566207 |
| IGHV3-7 | 1.049383417 | 0.961919189 | 1.144800486 | 0.2776635 |
| MRO | 0.869401779 | 0.675272239 | 1.119340333 | 0.277696047 |
| OR13A1 | 1.38046694 | 0.770890371 | 2.472062231 | 0.278088483 |
| DNAJB13 | 0.472175386 | 0.119430307 | 1.86677571 | 0.284643667 |
| TNFRSF9 | 1.219906923 | 0.84698056 | 1.757033124 | 0.285608372 |
| MARCO | 1.087769957 | 0.931775862 | 1.269879945 | 0.286767882 |
| UGT3A2 | 1.14562735 | 0.889358926 | 1.475739419 | 0.29263965 |
| COLEC10 | 0.772144271 | 0.47550705 | 1.253833723 | 0.295822975 |
| TMEM92 | 1.104009328 | 0.916622983 | 1.329703291 | 0.297123756 |
| CYP4F22 | 1.936847941 | 0.558882101 | 6.712292166 | 0.297196138 |
| IGKV3-11 | 1.041337991 | 0.964940396 | 1.123784242 | 0.297438757 |
| SIGLEC12 | 1.250865652 | 0.820408601 | 1.907177566 | 0.298284802 |
| MTATP6P2 | 0.762980523 | 0.457365599 | 1.2728095 | 0.300163419 |
| MIR133A1HG | 0.583944674 | 0.210186342 | 1.622328919 | 0.302140432 |
| SPINK5 | 0.83856148 | 0.600126886 | 1.171727801 | 0.302305418 |
| HSD17B6 | 0.924813719 | 0.79690289 | 1.073255507 | 0.303416137 |
| IGHV6-1 | 1.050734754 | 0.95611609 | 1.154717021 | 0.303998989 |
| SLC1A2 | 0.494791164 | 0.129056873 | 1.896979915 | 0.304805545 |
| USP30-AS1 | 1.341866787 | 0.764911603 | 2.354005964 | 0.305159835 |
| TRHR | 0.761632792 | 0.451472469 | 1.284872387 | 0.307482848 |
| IGHV4-31 | 1.048509618 | 0.957254445 | 1.148464157 | 0.307905128 |
| IGHV3-33 | 1.046628263 | 0.958758601 | 1.142551129 | 0.308380772 |
| ALOX5 | 1.073037501 | 0.936587529 | 1.229366656 | 0.309689794 |
| ZNF536 | 0.752781819 | 0.433910488 | 1.30598472 | 0.312370327 |
| HLA-DPA1 | 1.105078008 | 0.90992643 | 1.342083671 | 0.31352932 |
| WNT4 | 0.88914083 | 0.706111248 | 1.119613116 | 0.317704922 |
| IGKV1-16 | 1.04821939 | 0.955709021 | 1.149684543 | 0.317804631 |
| COL1A1 | 1.072553693 | 0.934384754 | 1.23115389 | 0.31952197 |
| PRRG3 | 1.218806962 | 0.824057326 | 1.802654212 | 0.321738279 |
| MT-RNR2 | 1.158062905 | 0.865985779 | 1.548650942 | 0.322354051 |
| CDON | 0.870949844 | 0.661125903 | 1.147366375 | 0.325865069 |
| NEB | 0.829411999 | 0.569778717 | 1.207353389 | 0.328892164 |
| IGLV2-23 | 1.04035756 | 0.960881346 | 1.126407393 | 0.329168225 |
| TMEM176A | 1.112837803 | 0.897723883 | 1.379497636 | 0.329305472 |
| CDH2 | 0.928987315 | 0.800904477 | 1.077553511 | 0.330474673 |
| EDN3 | 0.939819047 | 0.829193104 | 1.065204036 | 0.331355849 |
| CHGB | 1.073826981 | 0.928577281 | 1.241796896 | 0.336748074 |
| TMEM178B | 0.910957097 | 0.753066892 | 1.101951023 | 0.336904143 |
| ADM2 | 0.897052659 | 0.718447973 | 1.1200581 | 0.337528148 |
| LINC00964 | 4.152182612 | 0.226171364 | 76.2281315 | 0.337647192 |
| CASP5 | 1.77912891 | 0.541862361 | 5.841519744 | 0.34221558 |
| JCHAIN | 1.046461635 | 0.952224674 | 1.150024761 | 0.345566342 |
| RPLP0P2 | 1.286893494 | 0.761848623 | 2.173784679 | 0.345674036 |
| CXCL17 | 1.066479288 | 0.932868745 | 1.21922626 | 0.345967984 |
| ADAM12 | 1.114471237 | 0.888828078 | 1.397397504 | 0.347754277 |
| MTND2P28 | 1.099334985 | 0.902118751 | 1.339665546 | 0.347820904 |
| S100A2 | 1.08314473 | 0.916330721 | 1.280326503 | 0.349282964 |
| BCHE | 0.886841398 | 0.689402948 | 1.140824344 | 0.349993514 |
| MT-TL1 | 1.098952344 | 0.901131806 | 1.340199343 | 0.351411451 |
| LRGUK | 0.551830283 | 0.157957355 | 1.927840981 | 0.351595096 |
| GTSF1 | 0.673950993 | 0.292668637 | 1.551959739 | 0.353819625 |
| IGLV3-10 | 1.047773935 | 0.949283788 | 1.156482638 | 0.354146478 |
| IGLV3-1 | 1.038967165 | 0.958065923 | 1.126699889 | 0.355364568 |
| GPC3 | 0.87397495 | 0.655238266 | 1.165732 | 0.35937966 |
| CXCL9 | 1.060422256 | 0.935072379 | 1.202575742 | 0.360692977 |
| NPTX1 | 1.232113089 | 0.787267473 | 1.928318795 | 0.361059938 |
| BMP8A | 0.941757645 | 0.827882085 | 1.07129684 | 0.361457786 |
| LAMB4 | 0.541446815 | 0.144969391 | 2.022252084 | 0.361490862 |
| DIO1 | 0.956561033 | 0.869302762 | 1.052578054 | 0.362828056 |
| MT-TC | 1.115227941 | 0.880689065 | 1.412227551 | 0.36530322 |
| GBP5 | 1.129106868 | 0.868070701 | 1.468638808 | 0.365345035 |
| IGKV1-27 | 1.042972782 | 0.95200679 | 1.142630741 | 0.366179996 |
| LRRN4 | 0.65674806 | 0.262673603 | 1.642030296 | 0.368511305 |
| CST4 | 0.924136367 | 0.778007406 | 1.097711947 | 0.368982365 |
| DPP6 | 0.8904231 | 0.691200333 | 1.147067295 | 0.369108204 |
| IGHV1-2 | 1.037417876 | 0.95746169 | 1.124051084 | 0.369349247 |
| IGHV3-21 | 1.038440927 | 0.956258063 | 1.127686762 | 0.369880881 |
| IGHV3-23 | 1.036150245 | 0.958601633 | 1.119972357 | 0.370932466 |
| C2CD4B | 1.093105642 | 0.899404543 | 1.328523359 | 0.371016992 |
| ERICH3 | 0.710516986 | 0.335860938 | 1.503105395 | 0.37134208 |
| GLDC | 0.882564606 | 0.670598167 | 1.161530588 | 0.372692109 |
| CXCL5 | 1.152801456 | 0.842999043 | 1.576456352 | 0.373224133 |
| GPA33 | 1.356785038 | 0.693022822 | 2.656284294 | 0.373378743 |
| CXCL10 | 1.073203627 | 0.918478193 | 1.253993871 | 0.373783217 |
| HLA-DQA1 | 1.088021933 | 0.902698132 | 1.311392684 | 0.375895274 |
| DERL3 | 1.115888384 | 0.875119107 | 1.422899896 | 0.376565335 |
| GOLGA8Q | 0.715601249 | 0.339252449 | 1.509451588 | 0.379546399 |
| ATP12A | 0.788837938 | 0.463509476 | 1.342508243 | 0.381957615 |
| SELE | 0.911710114 | 0.740912135 | 1.121881113 | 0.382477643 |
| PTPN7 | 1.121268773 | 0.865553244 | 1.452531859 | 0.386114299 |
| STXBP5L | 0.883120612 | 0.666224514 | 1.17062942 | 0.387382723 |
| AC067956.1 | 0.576375021 | 0.165090032 | 2.012284811 | 0.387718563 |
| SSR1P1 | 1.154180773 | 0.833292741 | 1.59863778 | 0.388291061 |
| KRT19 | 1.068499372 | 0.918560431 | 1.242913226 | 0.390433114 |
| KLK4 | 0.890925706 | 0.684242139 | 1.16004053 | 0.39110976 |
| SCUBE3 | 0.917309801 | 0.752958936 | 1.11753408 | 0.39154815 |
| CCDC178 | 1.242714429 | 0.755535609 | 2.044032252 | 0.392077669 |
| ENPP3 | 1.089570742 | 0.895043739 | 1.326375853 | 0.392598122 |
| IFNG | 1.167463727 | 0.816605944 | 1.669068862 | 0.395868264 |
| MROH2A | 1.098707888 | 0.883180684 | 1.366831323 | 0.398145531 |
| ARHGAP24 | 0.899065691 | 0.700588767 | 1.153771163 | 0.403130382 |
| IGHV3-74 | 1.041490968 | 0.946732307 | 1.145734046 | 0.403561148 |
| LAIR2 | 1.25255273 | 0.736756318 | 2.129453532 | 0.405595482 |
| HLA-DRA | 1.088743298 | 0.890539913 | 1.331059903 | 0.406948399 |
| SCUBE1 | 0.858128226 | 0.597230329 | 1.232998422 | 0.408031944 |
| SLC6A3 | 0.720939585 | 0.330757397 | 1.571405177 | 0.410476581 |
| OXCT2P1 | 0.77890544 | 0.429408038 | 1.412860569 | 0.410846229 |
| STAC | 1.097963918 | 0.878376246 | 1.372446912 | 0.411704423 |
| RNU4-2 | 0.925272848 | 0.768226246 | 1.114424099 | 0.413135635 |
| IGHV3-72 | 1.048899915 | 0.935383451 | 1.176192533 | 0.413964701 |
| IGHG3 | 1.033542082 | 0.954797651 | 1.118780754 | 0.414524083 |
| IGKC | 1.028940642 | 0.960784662 | 1.10193146 | 0.414558786 |
| PIM2 | 1.105252541 | 0.868456522 | 1.40661409 | 0.415938925 |
| MZB1 | 1.04812594 | 0.935052525 | 1.174873022 | 0.419658897 |
| DPP10 | 0.850769852 | 0.57409423 | 1.260784908 | 0.420655865 |
| HHIPL2 | 0.885618908 | 0.65858305 | 1.190921709 | 0.421528963 |
| IGHV3-11 | 1.036700485 | 0.94918817 | 1.132281174 | 0.423119269 |
| FCGR1A | 1.147171342 | 0.819793401 | 1.605285045 | 0.42319402 |
| IGLV2-11 | 1.033122793 | 0.953642692 | 1.119227059 | 0.42497357 |
| SIRPG | 1.109469737 | 0.858611234 | 1.433621002 | 0.426999471 |
| GOLGA8M | 0.86169568 | 0.596559953 | 1.244668605 | 0.42755152 |
| TMPRSS4 | 1.054878057 | 0.924395188 | 1.203779217 | 0.427764238 |
| IL21R-AS1 | 1.173533107 | 0.789899606 | 1.743487327 | 0.42820806 |
| RRM2P3 | 0.756076242 | 0.378547923 | 1.510116022 | 0.428254243 |
| PITX3 | 1.196630308 | 0.765879536 | 1.86964663 | 0.430440935 |
| HLA-DQB2 | 1.064944081 | 0.910351176 | 1.245789456 | 0.431704744 |
| ASIP | 0.839379052 | 0.541667378 | 1.300719263 | 0.433340661 |
| ADGRV1 | 0.829358871 | 0.51910532 | 1.325041587 | 0.433824956 |
| FASLG | 1.14861572 | 0.811678841 | 1.625418832 | 0.434128216 |
| PSMB9 | 1.110457546 | 0.853214406 | 1.445259191 | 0.435822754 |
| LRRC15 | 1.069118789 | 0.903477791 | 1.265127927 | 0.436476752 |
| IGHV3-30 | 1.033635594 | 0.95066447 | 1.123848186 | 0.438403943 |
| SLC34A2 | 1.040451831 | 0.940967865 | 1.150453754 | 0.439316539 |
| NCF4 | 1.124976991 | 0.834412931 | 1.516722935 | 0.43982714 |
| IGKV1-17 | 1.034261129 | 0.948397569 | 1.127898381 | 0.446168391 |
| CACNG4 | 1.070042375 | 0.898469146 | 1.274379526 | 0.447707405 |
| SLC4A1 | 1.43437081 | 0.564772085 | 3.642920175 | 0.448123749 |
| DLGAP2 | 0.771945944 | 0.395132834 | 1.508101802 | 0.448726702 |
| CPNE7 | 1.227490101 | 0.722149105 | 2.086455468 | 0.448878275 |
| IGHG2 | 1.030688074 | 0.953097574 | 1.11459512 | 0.449074637 |
| LGALS17A | 1.077284758 | 0.887881328 | 1.307091852 | 0.450498579 |
| SLC6A1 | 0.767319743 | 0.383547821 | 1.535087817 | 0.454106045 |
| ADAMTS14 | 1.091560661 | 0.866989403 | 1.374301315 | 0.455987305 |
| IGLV3-21 | 1.031618621 | 0.950433407 | 1.119738607 | 0.45666293 |
| STEAP1 | 1.101296311 | 0.854022689 | 1.420165506 | 0.457056072 |
| SFN | 1.050235715 | 0.922789248 | 1.19528382 | 0.457735757 |
| IGKV1-5 | 1.030309182 | 0.952076244 | 1.114970589 | 0.458644788 |
| RAC2 | 1.079402034 | 0.881613789 | 1.321563667 | 0.459380448 |
| TREM2 | 1.088800315 | 0.868868761 | 1.364401829 | 0.459909421 |
| KCNN4 | 1.05148752 | 0.920335477 | 1.201329334 | 0.460135161 |
| GPR84 | 1.172024596 | 0.768918382 | 1.786459637 | 0.460455311 |
| NOL4 | 0.833659338 | 0.51293872 | 1.354914076 | 0.462827735 |
| VSX1 | 1.204969988 | 0.731101075 | 1.985980764 | 0.464541712 |
| IGLC2 | 1.029436786 | 0.952424434 | 1.1126763 | 0.464604202 |
| KLK1 | 1.061450181 | 0.904518555 | 1.245609038 | 0.465035244 |
| SLC14A2 | 0.912272658 | 0.712455129 | 1.168131674 | 0.466665164 |
| MYBPH | 1.080381873 | 0.877148077 | 1.330704612 | 0.467133923 |
| IGKJ1 | 1.035637938 | 0.942306381 | 1.138213601 | 0.467397191 |
| IGHV1-18 | 1.030245999 | 0.95064273 | 1.116514948 | 0.467676731 |
| LAX1 | 1.097894666 | 0.852467622 | 1.413980621 | 0.469388017 |
| TRBV9 | 1.115928971 | 0.82843085 | 1.50320026 | 0.470516146 |
| PLEKHD1 | 0.875494651 | 0.609977187 | 1.256589428 | 0.470802125 |
| GZMH | 0.913926318 | 0.715137752 | 1.167972621 | 0.472003678 |
| CFTR | 0.657341127 | 0.208975884 | 2.067690059 | 0.473031482 |
| PROX1-AS1 | 0.757282016 | 0.353830249 | 1.620766039 | 0.473917754 |
| IGHV4-34 | 1.032536928 | 0.945678606 | 1.12737298 | 0.47511744 |
| SLITRK5 | 0.807711978 | 0.449288989 | 1.45206906 | 0.475479385 |
| PEG3 | 0.855651837 | 0.555083932 | 1.318971823 | 0.480152603 |
| ARHGEF38 | 1.084307678 | 0.865208731 | 1.358889595 | 0.482173642 |
| MEI1 | 1.128396285 | 0.803696661 | 1.584277051 | 0.485351029 |
| RP1 | 1.337591359 | 0.590442878 | 3.03018414 | 0.485709473 |
| CORIN | 1.205487666 | 0.712570162 | 2.039378843 | 0.486004217 |
| CNTN5 | 0.845878212 | 0.528172725 | 1.354689321 | 0.486061976 |
| IGLC3 | 1.028793915 | 0.949691164 | 1.114485382 | 0.486789635 |
| HLA-G | 1.055761476 | 0.905807176 | 1.230540366 | 0.487528734 |
| COL4A6 | 0.800898803 | 0.427121592 | 1.501771169 | 0.488821708 |
| STMN2 | 0.925142912 | 0.741115948 | 1.154865727 | 0.491717667 |
| NR2F1-AS1 | 1.119519066 | 0.811347785 | 1.544741924 | 0.491899892 |
| LTB | 1.05767075 | 0.900740736 | 1.241941627 | 0.493824723 |
| CCNA1 | 1.089307998 | 0.852490691 | 1.391911874 | 0.494006821 |
| IL2RG | 1.079006658 | 0.866201223 | 1.344093425 | 0.497497398 |
| TRAC | 1.060904759 | 0.893891921 | 1.259121914 | 0.498731417 |
| CHI3L1 | 1.035448708 | 0.935438898 | 1.146150784 | 0.501476256 |
| VN2R19P | 0.703408838 | 0.249963609 | 1.979424107 | 0.50510858 |
| CD79A | 1.037922167 | 0.930297084 | 1.157998282 | 0.505160022 |
| PRR18 | 0.735489823 | 0.297329913 | 1.819343618 | 0.506156011 |
| FMN2 | 1.234118196 | 0.661583325 | 2.302125315 | 0.508433459 |
| TBX22 | 0.905486522 | 0.673766311 | 1.216899433 | 0.510335374 |
| CD70 | 0.934088205 | 0.762109988 | 1.144875135 | 0.511330614 |
| IGKV2-30 | 1.035622401 | 0.932342445 | 1.150343164 | 0.513749188 |
| CCL17 | 1.047409808 | 0.910880871 | 1.20440262 | 0.515669885 |
| BCL2A1 | 1.077978315 | 0.859258996 | 1.35237135 | 0.516357405 |
| SNX20 | 1.104115679 | 0.818583004 | 1.489245963 | 0.516496215 |
| AMD1P4 | 0.841332886 | 0.499139466 | 1.418122733 | 0.516617323 |
| RNF183 | 1.091966805 | 0.836803904 | 1.424935398 | 0.517043369 |
| JPH3 | 1.145045805 | 0.758009078 | 1.729702102 | 0.519868367 |
| STAB2 | 0.633230916 | 0.157240906 | 2.550108639 | 0.520311706 |
| S100A8 | 1.075177876 | 0.861957008 | 1.341142834 | 0.520388433 |
| SPX | 0.930523762 | 0.746784691 | 1.159470035 | 0.521135203 |
| IGHV3-15 | 1.028291029 | 0.944114793 | 1.119972324 | 0.522021539 |
| NPPC | 1.067508443 | 0.873495431 | 1.304613893 | 0.523248416 |
| IGHV2-5 | 1.028915914 | 0.942667188 | 1.123055912 | 0.523363863 |
| SNORD3A | 0.930480341 | 0.743928254 | 1.163813391 | 0.527942238 |
| HLA-DRB5 | 1.058626226 | 0.886806249 | 1.263736568 | 0.528363388 |
| MAGEL2 | 0.879263676 | 0.587479817 | 1.315967954 | 0.531706409 |
| CHRM3-AS2 | 1.475029912 | 0.43452287 | 5.007131716 | 0.533083357 |
| LMX1B | 0.80925464 | 0.415778367 | 1.57510136 | 0.533367274 |
| WNT7A | 1.136818194 | 0.75918743 | 1.70228794 | 0.533605701 |
| IGKV1-12 | 1.030299154 | 0.93682082 | 1.133104992 | 0.538490801 |
| FBXW10 | 0.639517652 | 0.153830002 | 2.658667507 | 0.538604913 |
| FAM83E | 1.151998259 | 0.73369023 | 1.808801502 | 0.538754199 |
| NKG7 | 0.93200754 | 0.744216278 | 1.167184971 | 0.53964425 |
| CYSRT1 | 1.085583719 | 0.834843935 | 1.411631517 | 0.539985006 |
| B3GALT2 | 0.875729131 | 0.572651552 | 1.339211442 | 0.540352149 |
| ZBP1 | 1.113685915 | 0.788558705 | 1.57286491 | 0.540992831 |
| FCN1 | 1.100565576 | 0.808613718 | 1.497927329 | 0.54234647 |
| DCC | 1.78015297 | 0.273520188 | 11.58577953 | 0.546209281 |
| S100A10 | 1.062729999 | 0.871521789 | 1.295888486 | 0.547723613 |
| KCNIP1 | 0.783762374 | 0.353358704 | 1.738413268 | 0.548865411 |
| IGLV1-44 | 1.025971264 | 0.942900541 | 1.116360622 | 0.551727828 |
| IGKV3-15 | 1.02434245 | 0.946181432 | 1.1089601 | 0.552579379 |
| PLCD4 | 0.8389762 | 0.467332384 | 1.506167961 | 0.556471059 |
| IGHV4-28 | 1.034846628 | 0.923103387 | 1.160116578 | 0.556849872 |
| SPON1 | 1.056894159 | 0.878378377 | 1.271690302 | 0.55774295 |
| IGKV1-6 | 1.027949811 | 0.937419105 | 1.127223466 | 0.557838901 |
| INSL3 | 1.105229991 | 0.790397262 | 1.545467565 | 0.558615053 |
| IGLV2-8 | 1.02794528 | 0.937140803 | 1.127548278 | 0.559148034 |
| SPAG4 | 1.095180927 | 0.806547329 | 1.487105865 | 0.560218496 |
| NKD2 | 1.09910126 | 0.799739638 | 1.510521077 | 0.560251722 |
| FAM89B | 1.130784615 | 0.747319757 | 1.711013033 | 0.560805135 |
| IGHV5-51 | 1.025295239 | 0.942207656 | 1.115709813 | 0.562352137 |
| IGHGP | 1.021821962 | 0.949798128 | 1.099307413 | 0.562687236 |
| CD1B | 1.08433158 | 0.823100567 | 1.428470617 | 0.564818697 |
| IGHV3-53 | 1.031252285 | 0.928573903 | 1.145284476 | 0.565225804 |
| IGLV4-69 | 1.027520973 | 0.936024454 | 1.127961289 | 0.568303619 |
| AQP4 | 0.951518831 | 0.801828272 | 1.129154604 | 0.56932011 |
| TRBC2 | 1.051087751 | 0.88494223 | 1.248426647 | 0.57032262 |
| CD164L2 | 0.939458446 | 0.756037124 | 1.167379409 | 0.573083554 |
| CXCL11 | 1.054656784 | 0.875307921 | 1.270753875 | 0.57577557 |
| OSCAR | 1.078969178 | 0.826499525 | 1.408560381 | 0.576261398 |
| GNLY | 0.949695892 | 0.79164426 | 1.139302503 | 0.578391092 |
| SLC19A3 | 1.416111467 | 0.4150497 | 4.831642305 | 0.578468655 |
| UBASH3A | 1.116604781 | 0.754643302 | 1.652179557 | 0.581132869 |
| CATSPER1 | 1.067556416 | 0.845492011 | 1.347944967 | 0.582723841 |
| GZMA | 0.943187173 | 0.764160873 | 1.164155447 | 0.586001104 |
| SFRP1 | 0.950833153 | 0.79256083 | 1.140712043 | 0.587314298 |
| HCST | 1.093828369 | 0.791130062 | 1.512343617 | 0.587432294 |
| LY6G6C | 1.052349609 | 0.874109378 | 1.266934926 | 0.589950387 |
| IP6K3 | 0.943449181 | 0.763306012 | 1.166106834 | 0.590245944 |
| IGHV3-48 | 1.023912742 | 0.937690922 | 1.118062764 | 0.598522597 |
| IGKV3-20 | 1.019887656 | 0.947516643 | 1.097786344 | 0.600009763 |
| SLC7A4 | 0.888509914 | 0.571178459 | 1.382142226 | 0.600026701 |
| CIB4 | 0.941474377 | 0.751264306 | 1.179843095 | 0.600462103 |
| S100B | 1.041590858 | 0.894044381 | 1.213487315 | 0.601068895 |
| RGS17 | 1.093223093 | 0.782486603 | 1.527357434 | 0.601398036 |
| MYEOV | 1.078769749 | 0.811675522 | 1.433755411 | 0.601399801 |
| NEURL3 | 1.224587384 | 0.572403182 | 2.619856611 | 0.601572099 |
| IGHG4 | 1.021354859 | 0.942689049 | 1.106585198 | 0.605355043 |
| BPIFB1 | 0.894069504 | 0.582908195 | 1.371331342 | 0.607913847 |
| IGKV3D-20 | 1.026762459 | 0.928040822 | 1.135985749 | 0.608611713 |
| PTCHD3P2 | 1.080829698 | 0.801871535 | 1.456832902 | 0.609833873 |
| HLA-B | 1.074339755 | 0.814727055 | 1.41667802 | 0.611390787 |
| DOK2 | 1.07125853 | 0.821013941 | 1.397777531 | 0.612087713 |
| GABRB3 | 0.939437455 | 0.737682447 | 1.196372146 | 0.61253046 |
| FGF11 | 0.692280364 | 0.166559469 | 2.877363294 | 0.612887418 |
| NLRP7 | 0.714459301 | 0.193986261 | 2.631382703 | 0.613230697 |
| FTH1P20 | 1.083072932 | 0.790992794 | 1.483005893 | 0.618699763 |
| MT3 | 0.913925789 | 0.639227392 | 1.306671707 | 0.621684146 |
| CNTNAP2 | 1.093257 | 0.766322958 | 1.559669924 | 0.622840674 |
| TEKT4 | 0.899752614 | 0.59057471 | 1.370791455 | 0.622890671 |
| SLAMF7 | 1.045912332 | 0.874329324 | 1.251167697 | 0.623423573 |
| SLPI | 0.97220554 | 0.867910315 | 1.089033734 | 0.626362421 |
| IGKV1OR2-108 | 1.0323342 | 0.908020101 | 1.173667741 | 0.626903021 |
| NAT16 | 1.058775518 | 0.840943324 | 1.33303347 | 0.626990762 |
| KCTD8 | 1.061453436 | 0.834162908 | 1.350675492 | 0.627611016 |
| RPH3A | 0.777377531 | 0.280653756 | 2.153243324 | 0.628055187 |
| RETN | 1.048798196 | 0.862207591 | 1.275768929 | 0.633594498 |
| TNFRSF17 | 1.043753048 | 0.875188737 | 1.244783417 | 0.633712881 |
| IGLV7-43 | 1.026202957 | 0.921375591 | 1.142956813 | 0.638012987 |
| FSCN1P1 | 0.947582554 | 0.757087877 | 1.186008553 | 0.638219655 |
| NKX3-2 | 1.10600565 | 0.725861289 | 1.68523727 | 0.639143963 |
| CNFN | 1.067867351 | 0.81116551 | 1.405805184 | 0.639724509 |
| IGLV3-19 | 1.019375773 | 0.940600709 | 1.104748229 | 0.640026843 |
| IGLV1-47 | 1.02056354 | 0.93702498 | 1.11154981 | 0.640390501 |
| POLR2J3 | 1.078635442 | 0.785102937 | 1.481913214 | 0.640440518 |
| MTATP8P2 | 0.953838508 | 0.781828237 | 1.163692811 | 0.641355074 |
| TENM2 | 0.854924466 | 0.438772013 | 1.665775897 | 0.645114117 |
| ALOX15B | 0.967955927 | 0.842604156 | 1.111955916 | 0.645327546 |
| B3GNT3 | 1.027743465 | 0.914601283 | 1.154882078 | 0.645611327 |
| GOLGA8IP | 0.817608117 | 0.345281696 | 1.936051175 | 0.647055961 |
| FTH1P10 | 1.191040652 | 0.561134084 | 2.528055013 | 0.648906537 |
| ISG15 | 0.941757101 | 0.727016644 | 1.219925905 | 0.649497883 |
| MT1H | 0.977269269 | 0.8849327 | 1.079240518 | 0.649786923 |
| IGKV4-1 | 1.018299367 | 0.941452992 | 1.10141835 | 0.650573263 |
| KRT222 | 1.100443868 | 0.725658192 | 1.668797679 | 0.652329816 |
| ETNPPL | 0.925586245 | 0.660595161 | 1.296875829 | 0.653178121 |
| ABCC8 | 1.075829626 | 0.781941411 | 1.48017405 | 0.653439431 |
| XKR4 | 1.244162569 | 0.479085667 | 3.231030697 | 0.65367283 |
| IGHV4-59 | 1.019267675 | 0.937381319 | 1.108307337 | 0.655144871 |
| SIT1 | 1.055425884 | 0.830589154 | 1.3411249 | 0.658967851 |
| IGLV7-46 | 1.023788093 | 0.921968974 | 1.136851769 | 0.660030718 |
| XDH | 0.939651941 | 0.711949981 | 1.240179499 | 0.660201782 |
| IGHG1 | 1.015077651 | 0.949320833 | 1.085389261 | 0.661422473 |
| HLA-DRB1 | 1.048503266 | 0.847804948 | 1.296712295 | 0.66217128 |
| TMSB10P1 | 0.932031887 | 0.679376203 | 1.278648612 | 0.662610207 |
| CD7 | 1.054199736 | 0.829828715 | 1.339236716 | 0.665542937 |
| UNC5D | 0.915565016 | 0.6128981 | 1.367697663 | 0.666618081 |
| DACH2 | 0.896035495 | 0.541636383 | 1.482322152 | 0.669075372 |
| CD2 | 1.042599662 | 0.860772654 | 1.262835257 | 0.669633394 |
| TMEM92-AS1 | 1.117900782 | 0.666859132 | 1.874012212 | 0.672423736 |
| P2RY6 | 1.081430302 | 0.74920788 | 1.560970632 | 0.675909081 |
| HAGLR | 1.047366148 | 0.84273279 | 1.301688815 | 0.676492495 |
| LINGO2 | 0.878143278 | 0.474960274 | 1.623579189 | 0.678572668 |
| PHEX | 0.969979472 | 0.838950645 | 1.121472619 | 0.680593289 |
| AZU1 | 1.078231341 | 0.752088515 | 1.545805849 | 0.681934343 |
| IGLV6-57 | 1.018792175 | 0.931615949 | 1.114125942 | 0.683326168 |
| AR | 0.943770523 | 0.714556208 | 1.246511875 | 0.68350282 |
| SPOCD1 | 1.074857377 | 0.75453659 | 1.531162829 | 0.689260501 |
| TNFRSF13B | 1.09474317 | 0.700943388 | 1.709785167 | 0.690683022 |
| WNT16 | 0.857079958 | 0.398609555 | 1.842871161 | 0.692956816 |
| XCL2 | 0.943553016 | 0.706177453 | 1.260720362 | 0.694336429 |
| SPINK2 | 1.055695564 | 0.800806047 | 1.391714173 | 0.700665736 |
| IGHV1-24 | 1.018618208 | 0.926400336 | 1.120015843 | 0.703201922 |
| PNOC | 1.0639386 | 0.769833535 | 1.470402746 | 0.707339782 |
| CFAP47 | 0.750768718 | 0.161314671 | 3.494125275 | 0.714837985 |
| CST6 | 0.981223552 | 0.886306132 | 1.086305989 | 0.714988793 |
| CITED4 | 1.059603166 | 0.773079584 | 1.452319907 | 0.718907193 |
| GJA3 | 0.934230496 | 0.643624961 | 1.356048433 | 0.720449488 |
| HLA-DOB | 1.045112103 | 0.820184988 | 1.331723117 | 0.721206179 |
| MUC16 | 1.066443786 | 0.746697829 | 1.523109221 | 0.723529352 |
| ADCY5 | 0.902397243 | 0.510272113 | 1.595855945 | 0.724035688 |
| IGFN1 | 1.020826502 | 0.909255431 | 1.14608801 | 0.727050065 |
| FGF12 | 0.941592455 | 0.662894947 | 1.337461321 | 0.736796151 |
| PSTPIP1 | 1.060387159 | 0.752810765 | 1.493630244 | 0.737277395 |
| IGHV3-73 | 1.018223238 | 0.916007205 | 1.131845423 | 0.737941534 |
| PODNL1 | 1.046176409 | 0.802688916 | 1.36352335 | 0.738407858 |
| DHRS9 | 1.044366545 | 0.809327086 | 1.347664622 | 0.738599953 |
| COMP | 0.980019715 | 0.870240379 | 1.103647526 | 0.739161737 |
| LINC00892 | 0.877331934 | 0.405091897 | 1.900090642 | 0.739947784 |
| OR4D10 | 0.940350726 | 0.653555643 | 1.352998015 | 0.740403134 |
| FCRL5 | 1.056217699 | 0.762437678 | 1.463196088 | 0.742229178 |
| VGF | 0.967663027 | 0.794534232 | 1.178516541 | 0.743798779 |
| IGHM | 1.015698659 | 0.924602258 | 1.115770329 | 0.745260966 |
| C2 | 1.060456208 | 0.743146082 | 1.513252099 | 0.746265364 |
| PRRT4 | 0.95936165 | 0.745296765 | 1.234910466 | 0.747413773 |
| BATF | 1.036365616 | 0.832096196 | 1.290780676 | 0.74979312 |
| TRPM3 | 1.12070788 | 0.549768445 | 2.284573013 | 0.753817652 |
| CD3D | 1.039356104 | 0.814819764 | 1.325766948 | 0.755915785 |
| ANKRD20A8P | 1.074058287 | 0.678809332 | 1.699448063 | 0.760239822 |
| TUBA4B | 1.075561276 | 0.669955607 | 1.726729422 | 0.7629641 |
| DPY19L2P4 | 0.955328409 | 0.709527173 | 1.286282475 | 0.763321669 |
| RYR3 | 0.877661879 | 0.373652527 | 2.061515222 | 0.764549902 |
| MT-TF | 0.959203776 | 0.73003414 | 1.260313504 | 0.764925382 |
| IGKV1-9 | 1.013375237 | 0.927979249 | 1.106629671 | 0.767372359 |
| CTSE | 0.9794183 | 0.849012107 | 1.129854568 | 0.775440562 |
| IGFL2 | 1.0211836 | 0.882935861 | 1.181077801 | 0.777604607 |
| SLC26A3 | 0.910688286 | 0.474997202 | 1.746016926 | 0.778164776 |
| SFRP2 | 1.01405116 | 0.918491635 | 1.119552662 | 0.782309473 |
| VGLL1 | 1.027287739 | 0.848188688 | 1.244204402 | 0.782981924 |
| TDH | 0.950698378 | 0.662397439 | 1.364479015 | 0.783897515 |
| ALOX5AP | 1.031134431 | 0.827869708 | 1.284306219 | 0.784320908 |
| SERPINA1 | 1.01638568 | 0.902267291 | 1.144937715 | 0.789106681 |
| APOA1 | 0.974447134 | 0.800964771 | 1.185504347 | 0.795808867 |
| ATRNL1 | 0.945092558 | 0.609732147 | 1.464905447 | 0.800615013 |
| IRS4 | 1.126183144 | 0.441180289 | 2.874762325 | 0.803720797 |
| GCK | 0.927058921 | 0.507577579 | 1.69321554 | 0.805346024 |
| CAPSL | 0.975838681 | 0.802409849 | 1.186751549 | 0.806473334 |
| PCED1B-AS1 | 0.957310496 | 0.673298114 | 1.361125728 | 0.808034025 |
| SAA1 | 1.023649058 | 0.846251177 | 1.23823449 | 0.809773725 |
| NCF1 | 1.043177393 | 0.739320377 | 1.47191814 | 0.809836572 |
| ALX4 | 1.109297122 | 0.476303683 | 2.583520028 | 0.809965242 |
| NOS1 | 0.923555247 | 0.478879381 | 1.781146421 | 0.81241092 |
| GDF15 | 1.020519625 | 0.861332452 | 1.209126977 | 0.814401908 |
| COL11A2 | 1.023690503 | 0.838300437 | 1.250079565 | 0.818330268 |
| SOWAHD | 0.941327369 | 0.561658965 | 1.577642788 | 0.818487924 |
| IGLV8-61 | 1.011228351 | 0.919128776 | 1.11255659 | 0.818736949 |
| IGHV1-3 | 0.989810338 | 0.905874135 | 1.081523877 | 0.820786421 |
| SFTPB | 1.00979713 | 0.926447388 | 1.100645603 | 0.824459994 |
| CXXC4 | 0.970809084 | 0.743520879 | 1.267577419 | 0.827671507 |
| AC138649.1 | 1.024640577 | 0.819719239 | 1.280790132 | 0.830692844 |
| MMEL1 | 1.040197861 | 0.723238144 | 1.496065437 | 0.831683378 |
| DRC1 | 0.92122571 | 0.431770268 | 1.965528597 | 0.831942917 |
| CKM | 0.945250415 | 0.539014015 | 1.657653274 | 0.844245489 |
| HLA-DRB6 | 1.017636354 | 0.851974077 | 1.215510867 | 0.847079079 |
| FCRLA | 1.023832175 | 0.796762888 | 1.315613889 | 0.853937908 |
| ASXL3 | 0.953777737 | 0.571240376 | 1.592485422 | 0.856413179 |
| XCL1 | 1.032961281 | 0.726333833 | 1.46903388 | 0.856774709 |
| LIPI | 1.023507675 | 0.791508805 | 1.323507654 | 0.859376516 |
| CBLN4 | 1.019008926 | 0.826716832 | 1.25602764 | 0.859913089 |
| P2RX1 | 0.959842483 | 0.60487241 | 1.523127153 | 0.86188849 |
| SYNGR3 | 1.043566779 | 0.645105213 | 1.688145748 | 0.862045275 |
| OR5A1 | 1.084745584 | 0.425363347 | 2.766277327 | 0.864768615 |
| MTATP8P1 | 1.024213625 | 0.772921812 | 1.35720526 | 0.867701395 |
| EPHA5 | 0.95654421 | 0.56508448 | 1.619185907 | 0.868600403 |
| EHF | 1.015156956 | 0.844821475 | 1.219835996 | 0.872467249 |
| TMEM61 | 0.952078935 | 0.518896344 | 1.746888965 | 0.874000494 |
| LST1 | 1.021922561 | 0.779085676 | 1.34045042 | 0.875517932 |
| IL32 | 1.020068887 | 0.791585373 | 1.314501972 | 0.877944637 |
| CCL13 | 1.011297788 | 0.864419624 | 1.183132807 | 0.888414751 |
| IGKV2-24 | 1.007948571 | 0.900435122 | 1.128299304 | 0.890578796 |
| PIWIL1 | 1.011175119 | 0.859976471 | 1.188957089 | 0.893020508 |
| CYP2S1 | 1.011358499 | 0.857653029 | 1.192610506 | 0.893177861 |
| CNTNAP3 | 1.112260927 | 0.227086786 | 5.447804296 | 0.895578706 |
| COL10A1 | 1.010181239 | 0.864162364 | 1.180873153 | 0.898807735 |
| CST2 | 0.992101904 | 0.876048295 | 1.123529598 | 0.900581703 |
| TNNI2 | 1.023522623 | 0.708950092 | 1.477676034 | 0.901241351 |
| UPK2 | 1.018616155 | 0.758600484 | 1.367754033 | 0.902374843 |
| CSTA | 1.016552077 | 0.779462095 | 1.325758021 | 0.903565145 |
| SYT2 | 1.04627563 | 0.499581298 | 2.191220322 | 0.904530339 |
| PENK | 0.984754205 | 0.75890103 | 1.277822544 | 0.907984351 |
| MAP7D2 | 1.027547131 | 0.643249026 | 1.641437554 | 0.90946838 |
| HLA-F | 1.017765768 | 0.750626078 | 1.379977579 | 0.90974182 |
| HHIP | 0.916884608 | 0.195718609 | 4.295337019 | 0.912306756 |
| C1QA | 0.987036382 | 0.769842634 | 1.265506452 | 0.918037343 |
| PDLIM4 | 1.007287392 | 0.87682663 | 1.157159073 | 0.918281013 |
| JSRP1 | 1.012363577 | 0.789892148 | 1.297493606 | 0.922683617 |
| SULT2B1 | 0.987775544 | 0.767965727 | 1.270500091 | 0.923700595 |
| TNFAIP8L2 | 0.985809 | 0.734296811 | 1.323469434 | 0.924231486 |
| MTND4P24 | 1.009452446 | 0.830538228 | 1.226908294 | 0.924697751 |
| CSF2 | 1.010449632 | 0.814276046 | 1.253884925 | 0.924797769 |
| IGLV3-25 | 1.004263589 | 0.91552842 | 1.101599179 | 0.928175877 |
| SFTPA2 | 1.00686291 | 0.862917091 | 1.174820767 | 0.93075866 |
| TMSB4XP4 | 0.987512336 | 0.7380283 | 1.321332276 | 0.932597474 |
| FALEC | 0.99307621 | 0.776011446 | 1.270858006 | 0.955969306 |
| POU2AF1 | 1.005803556 | 0.809715376 | 1.249378267 | 0.958289132 |
| DIRAS1 | 0.992308436 | 0.730788047 | 1.347416718 | 0.960544562 |
| PIP5K1B | 0.990396163 | 0.67133682 | 1.461091557 | 0.961203617 |
| RNASE2 | 1.00702923 | 0.722682057 | 1.403255913 | 0.966994522 |
| GOLGA8T | 0.944566645 | 0.06063302 | 14.71485572 | 0.967530048 |
| MMP7 | 0.997497128 | 0.878317152 | 1.132848787 | 0.969208266 |
| ZNF683 | 1.0045784 | 0.786272329 | 1.283496474 | 0.970851825 |
| IGHV4-4 | 0.998456586 | 0.912915056 | 1.092013487 | 0.97303681 |
| CTNNA2 | 0.985199882 | 0.323553538 | 2.999870793 | 0.979061052 |
| DEFB1 | 1.002095585 | 0.833311872 | 1.20506571 | 0.982252129 |
| NCR3 | 1.00396704 | 0.686579812 | 1.468073777 | 0.983707411 |
| MSI1 | 1.001214954 | 0.802360013 | 1.249353616 | 0.991424202 |
| UNC5B-AS1 | 1.000575615 | 0.788445389 | 1.269779208 | 0.996223157 |

# Supplementary Table 5. The GSEA results regarding the TIPRGPI risk groups (High vs Low)

| NAME | SIZE | ES | NES | NOM p-val | FWER p-val | q-val |
| --- | --- | --- | --- | --- | --- | --- |
| HALLMARK_INTERFERON_GAMMA_RESPONSE | 199 | 0.630324087 | 2.673315674 | 1.00E-10 | 1.25E-09 | 7.11E-10 |
| HALLMARK_ALLOGRAFT_REJECTION | 196 | 0.616952864 | 2.609613214 | 1.00E-10 | 1.25E-09 | 7.11E-10 |
| HALLMARK_E2F_TARGETS | 200 | 0.518548404 | 2.205467136 | 1.00E-10 | 1.25E-09 | 7.11E-10 |
| HALLMARK_EPITHELIAL_MESENCHYMAL_TRANSITION | 200 | 0.512078609 | 2.177950092 | 1.00E-10 | 1.25E-09 | 7.11E-10 |
| HALLMARK_INTERFERON_ALPHA_RESPONSE | 97 | 0.609038868 | 2.313058342 | 3.45E-10 | 3.45E-09 | 1.96E-09 |
| HALLMARK_G2M_CHECKPOINT | 199 | 0.475438353 | 2.016417949 | 1.58E-08 | 1.31E-07 | 7.46E-08 |
| HALLMARK_INFLAMMATORY_RESPONSE | 199 | 0.44279433 | 1.877968887 | 7.33E-07 | 5.24E-06 | 2.98E-06 |
| HALLMARK_MYC_TARGETS_V1 | 199 | 0.421090266 | 1.785918122 | 4.90E-06 | 2.72E-05 | 1.55E-05 |
| HALLMARK_COMPLEMENT | 196 | 0.419090435 | 1.772686375 | 4.82E-06 | 2.72E-05 | 1.55E-05 |
| HALLMARK_COAGULATION | 126 | 0.477211275 | 1.879265482 | 9.80E-06 | 4.45E-05 | 2.53E-05 |
| HALLMARK_KRAS_SIGNALING_UP | 194 | 0.410367747 | 1.735599816 | 8.93E-06 | 4.45E-05 | 2.53E-05 |
| HALLMARK_APOPTOSIS | 159 | 0.404915942 | 1.660482026 | 0.000188201 | 0.000784169 | 0.000445738 |
| HALLMARK_IL6_JAK_STAT3_SIGNALING | 86 | 0.44084817 | 1.632752161 | 0.002458695 | 0.009456519 | 0.005375285 |
| HALLMARK_DNA_REPAIR | 148 | 0.363254906 | 1.463271456 | 0.004636959 | 0.016560569 | 0.009413376 |
| HALLMARK_IL2_STAT5_SIGNALING | 198 | 0.335662055 | 1.423580501 | 0.005322544 | 0.017741812 | 0.010084819 |
| HALLMARK_P53_PATHWAY | 199 | 0.332366538 | 1.409625135 | 0.007419762 | 0.02182283 | 0.012404556 |
| HALLMARK_MITOTIC_SPINDLE | 198 | 0.330460569 | 1.401520413 | 0.007319495 | 0.02182283 | 0.012404556 |
| HALLMARK_TNFA_SIGNALING_VIA_NFKB | 199 | 0.327946205 | 1.390877723 | 0.010844197 | 0.030122771 | 0.017122417 |
| HALLMARK_APICAL_JUNCTION | 196 | 0.315186779 | 1.333190314 | 0.015624038 | 0.041115889 | 0.023371137 |
| HALLMARK_KRAS_SIGNALING_DN | 177 | -0.347170002 | -1.32957919 | 0.023962247 | 0.059905616 | 0.034051614 |
| HALLMARK_ANGIOGENESIS | 35 | 0.473446877 | 1.466089941 | 0.034451534 | 0.082027462 | 0.046626136 |
| HALLMARK_ESTROGEN_RESPONSE_LATE | 198 | 0.298422088 | 1.265641615 | 0.036643763 | 0.083281279 | 0.047338832 |
| HALLMARK_HYPOXIA | 195 | -0.337800122 | -1.30815218 | 0.045069443 | 0.097977049 | 0.055692217 |

# Supplementary Table 6. Results of drug sensitive analysis.

| Variables | p value | Variables | p value | Variables | p value |
| --- | --- | --- | --- | --- | --- |
| RO.3306_1052 | 1.75E-28 | BPD.00008900_1998 | 6.71E-14 | Alisertib_1051 | 3.48E-07 |
| OF.1_1853 | 7.35E-25 | AZD1332_1463 | 8.27E-14 | JAK_8517_1739 | 4.12E-07 |
| AT13148_2170 | 1.47E-24 | AZD1208_1449 | 9.30E-14 | Pevonedistat_1529 | 6.49E-07 |
| Ipatasertib_1924 | 7.27E-24 | CDK9_5038_1709 | 9.48E-14 | Vinorelbine_2048 | 7.67E-07 |
| AGI.6780_1634 | 2.19E-23 | Paclitaxel_1080 | 1.13E-13 | Dabrafenib_1373 | 7.89E-07 |
| Zoledronate_1802 | 2.55E-23 | Fulvestrant_1816 | 1.60E-13 | VSP34_8731_1734 | 1.08E-06 |
| Rapamycin_1084 | 3.39E-23 | GSK591_2110 | 1.60E-13 | JAK1_8709_1718 | 1.11E-06 |
| AZD4547_1786 | 4.63E-23 | Cediranib_1922 | 1.60E-13 | Osimertinib_1919 | 2.00E-06 |
| GDC0810_1925 | 6.52E-23 | Alpelisib_1560 | 1.87E-13 | MK.1775_1179 | 2.66E-06 |
| PD173074_1049 | 8.10E-23 | Taselisib_1561 | 1.89E-13 | BMS.536924_1091 | 1.58E-05 |
| LY2109761_1852 | 2.03E-22 | Podophyllotoxin.bromide_1825 | 1.89E-13 | Erlotinib_1168 | 1.90E-05 |
| GSK1904529A_1093 | 3.60E-22 | GNE.317_1926 | 2.90E-13 | Ulixertinib_1908 | 7.64E-05 |
| I.BRD9_1928 | 3.62E-22 | BI.2536_1086 | 4.43E-13 | UMI.77_1939 | 9.14E-05 |
| LCL161_1557 | 4.37E-22 | Doramapimod_1042 | 5.08E-13 | WZ4003_1614 | 0.000104467 |
| Tamoxifen_1199 | 4.91E-22 | AGI.5198_1913 | 6.02E-13 | Mirin_1048 | 0.000281797 |
| AZD5991_1720 | 1.01E-21 | Talazoparib_1259 | 8.47E-13 | Bortezomib_1191 | 0.000285217 |
| Afuresertib_1912 | 1.61E-21 | IAP_5620_1428 | 1.39E-12 | SCH772984_1564 | 0.000365804 |
| BIBR.1532_2043 | 3.19E-21 | P22077_1933 | 2.46E-12 | Sapitinib_1549 | 0.000451199 |
| MIRA.1_1931 | 6.65E-21 | Nelarabine_1814 | 2.63E-12 | Entospletinib_1630 | 0.000582436 |
| Cyclophosphamide_1512 | 8.98E-21 | Olaparib_1017 | 3.73E-12 | IGF1R_3801_1738 | 0.000626822 |
| TAF1_5496_1732 | 1.05E-20 | Temozolomide_1375 | 3.73E-12 | VX.11e_2096 | 0.000974912 |
| MK.2206_1053 | 1.83E-20 | AZD8186_1918 | 4.50E-12 | Epirubicin_1511 | 0.001012318 |
| Sorafenib_1085 | 2.42E-20 | Ibrutinib_1799 | 4.79E-12 | AZD3759_1915 | 0.001030376 |
| KRAS..G12C..Inhibitor.12_1855 | 3.14E-20 | CZC24832_1615 | 5.20E-12 | AZD8055_1059 | 0.001198965 |
| Tozasertib_1096 | 4.57E-20 | Axitinib_1021 | 5.94E-12 | MK.8776_2046 | 0.001476117 |
| PFI3_1620 | 6.57E-20 | YK.4.279_1239 | 6.25E-12 | VE.822_1613 | 0.001554076 |
| Dihydrorotenone_1827 | 2.34E-19 | Linsitinib_1510 | 8.16E-12 | PD0325901_1060 | 0.002183096 |
| AZD5363_1916 | 2.49E-19 | Wee1.Inhibitor_1046 | 1.05E-11 | Dactinomycin_1911 | 0.002436528 |
| CDK9_5576_1708 | 6.70E-19 | AMG.319_2045 | 1.25E-11 | Foretinib_2040 | 0.00256518 |
| BDP.00009066_1866 | 9.96E-19 | ERK_2440_1713 | 1.49E-11 | SB505124_1194 | 0.002634507 |
| Uprosertib_1553 | 1.07E-18 | Daporinad_1248 | 2.37E-11 | PRT062607_1631 | 0.00314431 |
| LGK974_1598 | 2.93E-18 | Sabutoclax_1849 | 2.67E-11 | Dasatinib_1079 | 0.003448797 |
| Carmustine_1807 | 3.49E-18 | X5.Fluorouracil_1073 | 4.10E-11 | Teniposide_1809 | 0.011373302 |
| PAK_5339_1730 | 3.92E-18 | Trametinib_1372 | 5.62E-11 | AZD7762_1022 | 0.013209233 |
| Nilotinib_1013 | 8.41E-18 | Dactinomycin_1811 | 5.78E-11 | Lapatinib_1558 | 0.013441593 |
| ML323_1629 | 1.10E-17 | Vinblastine_1004 | 7.30E-11 | Camptothecin_1003 | 0.014913097 |
| Pyridostatin_2044 | 1.52E-17 | RVX.208_1625 | 7.64E-11 | Mitoxantrone_1810 | 0.015016062 |
| NVP.ADW742_1932 | 4.01E-17 | Ruxolitinib_1507 | 8.72E-11 | WIKI4_1940 | 0.020583952 |
| Fulvestrant_1200 | 6.39E-17 | AZD5438_1401 | 9.55E-11 | Luminespib_1559 | 0.020618018 |
| GSK2606414_1618 | 1.01E-16 | MIM1_1996 | 1.42E-10 | AZ960_1250 | 0.023469344 |
| EPZ004777_1237 | 1.23E-16 | Venetoclax_1909 | 1.54E-10 | Entinostat_1593 | 0.030306565 |
| Buparlisib_1873 | 2.40E-16 | Vincristine_1818 | 1.70E-10 | Staurosporine_1034 | 0.045457241 |
| PLX.4720_1036 | 2.73E-16 | Nutlin.3a...._1047 | 1.81E-10 | AZD6482_2169 | 0.046955959 |
| PCI.34051_1621 | 3.01E-16 | Oxaliplatin_1806 | 2.69E-10 | Irinotecan_1088 | 0.054693346 |
| GSK2578215A_1927 | 3.98E-16 | BMS.754807_2171 | 3.00E-10 | Afatinib_1032 | 0.082174808 |
| Dinaciclib_1180 | 5.20E-16 | JQ1_2172 | 3.81E-10 | KU.55933_1030 | 0.110999366 |
| AZD2014_1441 | 5.81E-16 | Navitoclax_1011 | 8.85E-10 | Ribociclib_1632 | 0.11654812 |
| EPZ5676_1563 | 9.20E-16 | NU7441_1038 | 9.35E-10 | Gefitinib_1010 | 0.116838441 |
| Uprosertib_2106 | 1.12E-15 | OTX015_1626 | 9.65E-10 | ULK1_4989_1733 | 0.145198187 |
| Selumetinib_1736 | 1.86E-15 | Palbociclib_1054 | 9.95E-10 | Cisplatin_1005 | 0.163942494 |
| ERK_6604_1714 | 2.93E-15 | Crizotinib_1083 | 1.04E-09 | Elephantin_1835 | 0.167364013 |
| Picolinici.acid_1635 | 3.51E-15 | ZM447439_1050 | 1.06E-09 | MG.132_1862 | 0.170644446 |
| Telomerase.Inhibitor.IX_1930 | 6.81E-15 | Ulixertinib_2047 | 3.21E-09 | PRIMA.1MET_1131 | 0.174960912 |
| IWP.2_1576 | 7.55E-15 | Cytarabine_1006 | 4.88E-09 | ABT737_1910 | 0.21064053 |
| MN.64_1854 | 8.45E-15 | Acetalax_1804 | 5.32E-09 | Sepantronium.bromide_1941 | 0.236937381 |
| Savolitinib_1936 | 1.11E-14 | Niraparib_1177 | 5.38E-09 | Fludarabine_1813 | 0.319328003 |
| VE821_2111 | 1.31E-14 | Sinularin_1838 | 1.47E-08 | SB216763_1025 | 0.333448324 |
| Eg5_9814_1712 | 1.34E-14 | Docetaxel_1007 | 1.87E-08 | Topotecan_1808 | 0.353753681 |
| BMS.345541_1249 | 1.68E-14 | Vorinostat_1012 | 3.39E-08 | AZD5582_1617 | 0.613501271 |
| Wnt.C59_1622 | 2.37E-14 | Leflunomide_1578 | 4.71E-08 | IRAK4_4710_1716 | 0.695254511 |
| AZ6102_2109 | 3.46E-14 | OSI.027_1594 | 4.71E-08 | AZD6738_1917 | 0.735701051 |
| Gallibiscoquinazole_1830 | 4.50E-14 | Oxaliplatin_1089 | 5.04E-08 | Obatoclax.Mesylate_1068 | 0.763086678 |
| Dactolisib_1057 | 4.70E-14 | GSK343_1627 | 5.59E-08 | Gemcitabine_1190 | 0.773077801 |
| AZD5153_1706 | 4.86E-14 | LJI308_2107 | 2.01E-07 | PF.4708671_1129 | 0.77403133 |
| I.BET.762_1624 | 5.71E-14 | Docetaxel_1819 | 2.85E-07 | XAV939_1268 | 0.850323956 |
| Pictilisib_1058 | 5.93E-14 | GSK269962A_1192 | 3.25E-07 | WEHI.539_1997 | 0.919636325 |

# Supplementary Table 7. Primer sequences for PCR.

| Primer | Primer sequence |
| --- | --- |
| C2CD4A F | 5′‐GGAGCCAAGTCTCGCACCACC‐3′ |
| C2CD4A R | 5′‐CCTCGTCCATCCCTGCTTCTTC‐3′ |
| CERS1 F | 5′‐TTCTACGACTGGACGCCG‐3′ |
| CERS1 R | 5′‐GGAGGAGACGATGAGGATGA‐3′ |
| PLP1 F | 5′‐TGCCTCTTTCTTCTTCCTTTAT‐3′ |
| PLP1 R | 5′‐CAGATGGTGGTCTTGTAGTCG‐3′ |
| RNF223 F | 5′‐ATCTTCAAGACACCCAAGGAGC‐3′ |
| RNF223 R | 5′‐CAGAAGGGGCAAGGTACAGC‐3′ |
| MMP9 F | 5′‐TGGGCTACGTGACCTATGACAT‐3′ |
| MMP9 R | 5′‐GCCCAGCCCACCTCCACTCCTC‐3′ |
| SLC5A1 F | 5′‐TCGTGGTCATCTCCCTCC‐3′ |
| SLC5A1 R | 5′‐GTTTCTATTTCAATGGTCTCCT‐3′ |
| HORMAD2 F | 5′‐CCACTGCTCAGCTTTCTCAC‐3′ |
| HORMAD2 R | 5′‐GTCATCCAAATGGCGTTCTC‐3′ |
| SLITRK2 F | 5′‐GCCTGGCTAGACACCATAAC‐3′ |
| SLITRK2 R | 5′‐CTGGAATCACTGGCACTTTT‐3′ |
| ARHGEF37 F | 5′‐CTACTGGACTTTGAGCGGGT‐3′ |
| ARHGEF37 R | 5′‐GCGAGTTGAGTGCCTGGTAT‐3′ |
| F3 F | 5′‐GACGAGATTGTGAAGGATGTG‐3′ |
| F3 R | 5′‐AAACTCTGAATTGTTGGCTGT‐3′ |
| GAPDH F | 5′‐GGCATCCTGGGCTACACT‐3′ |
| GAPDH R | 5′‐CCACCACCCTGTTGCTGTA‐3′ |

**Supplementary Table 8. Roles of 10 genes in sex hormone metabolism.**

| **Gene** | **Role in Sex Hormone Metabolism** | **Association Type** | **References** |
| --- | --- | --- | --- |
| CERS1 | Acts as a rate-limiting enzyme in sphingosine-1-phosphate (S1P) synthesis regulated by sex hormones (estrogen, testosterone), directly receiving sex hormone signaling regulation (activation by estrogen; direct or indirect regulation by testosterone after conversion). | Direct | (1) |
| MMP9 | 17β-estradiol (E2) promotes its expression by activating the Wnt/β-catenin pathway, participating in extracellular matrix remodeling in endometriosis; E2 can also rapidly activate MMP9 activity in the hippocampus to enhance memory consolidation; Androgen receptor (AR) drives its expression via the PIP5K1α/AKT axis, while MMP9 forms a positive feedback loop by enhancing AR function, thereby participating in the progression of metastatic prostate cancer. | Direct | (2-4) |
| SLC5A1 | Estriol (E3) targets and downregulates intestinal SLC5A1 expression, participating in the regulation of postprandial blood glucose homeostasis; 17β-estradiol (E2) positively regulates SLC5A1 expression in polymorphonuclear neutrophils (PMN), synergizing with other transporters to enhance glucose uptake and participate in the maintenance of energy homeostasis through cellular glycogen storage and ATP production. | Direct | (5, 6) |
| HORMAD2 | Highly expressed primarily in the testis (the testis is a key organ for sex hormone synthesis); its ectopic expression in lung tissue may be associated with lung cancer development, with no clear direct involvement in sex hormone synthesis or metabolic regulation identified. | Indirect | (7) |
| SLITRK2 | No direct mechanism involved in sex hormone metabolism has been clearly identified to date. | Not yet reported | — |
| ARHGEF37 | No direct mechanism involved in sex hormone metabolism has been clearly identified to date. | Not yet reported | — |
| PLP1 | Serves as a potential diagnostic biomarker for uterine fibroids; anti-progesterone receptor (PR) drug (Lonaprisan) can improve the pathological phenotype of Pelizaeus-Merzbacher disease (PMD) by reducing PLP1 expression, and is indirectly associated with the progesterone signaling pathway. | Indirect | (8, 9) |
| RNF223 | No direct mechanism involved in sex hormone metabolism has been clearly identified to date. | Not yet reported | — |
| F3 | No direct mechanism involved in sex hormone metabolism has been clearly identified to date. | Not yet reported | — |
| C2CD4A | Highly expressed in non-invasive gonadotroph pituitary adenomas (gonadotropins regulate gonadal sex hormone synthesis); its expression difference may be associated with adenoma phenotypes related to follicle-stimulating hormone (FSH) secretion. | Indirect | (10) |

**Supplementary Figures**


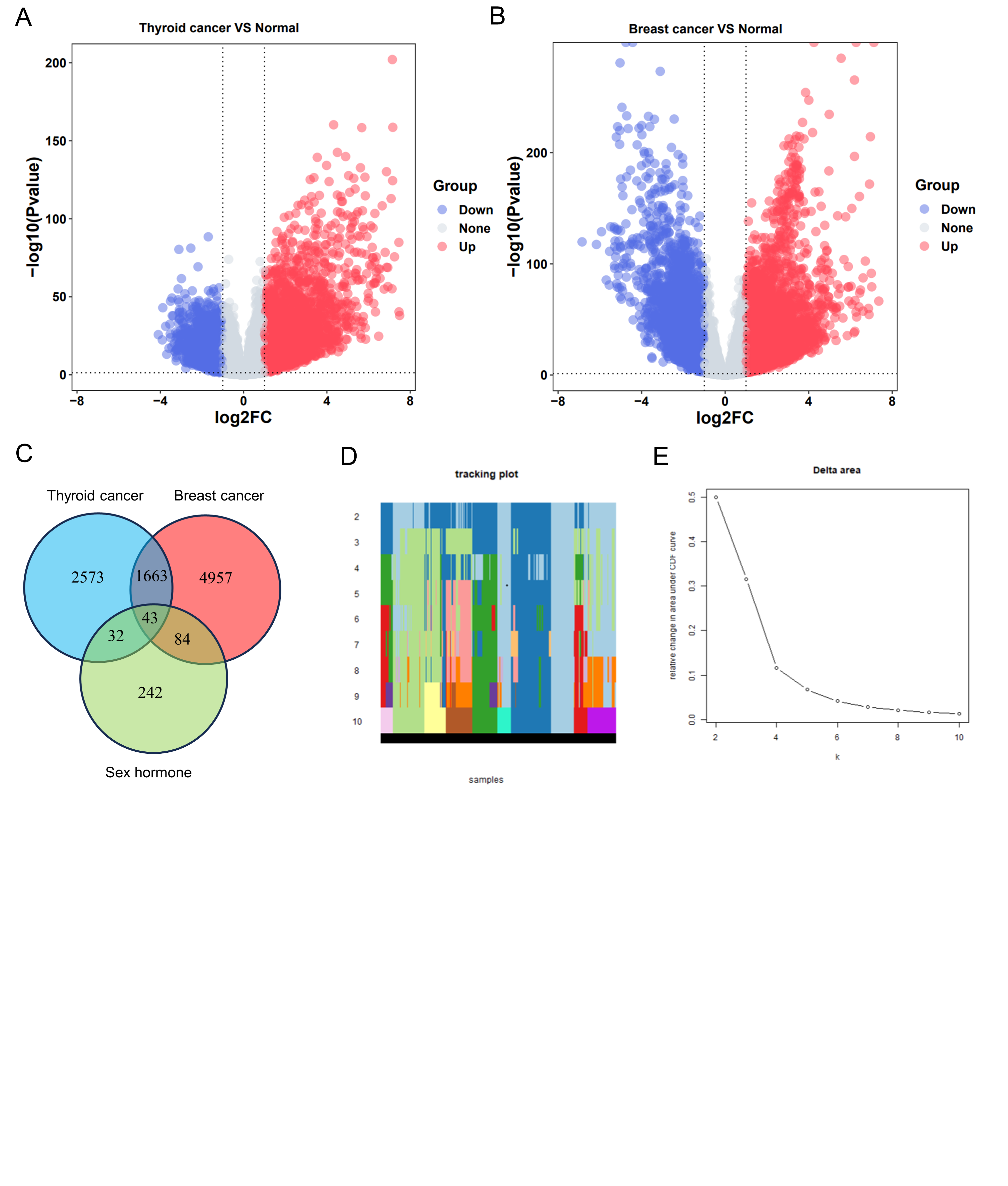


**Supplementary Figure 1. Construction of the consensus clustering.** (A) Volcano plot of the differentially expressed genes between thyroid cancer and normal tissues. (B) Volcano plot of the differentially expressed genes between breast cancer and normal tissues. (C) Wayne diagram for differential gene screening. (D) Tracking plot of the THCA samples (K = 2–10). (E) Relative change in area under the CDF curve from k 2–10.


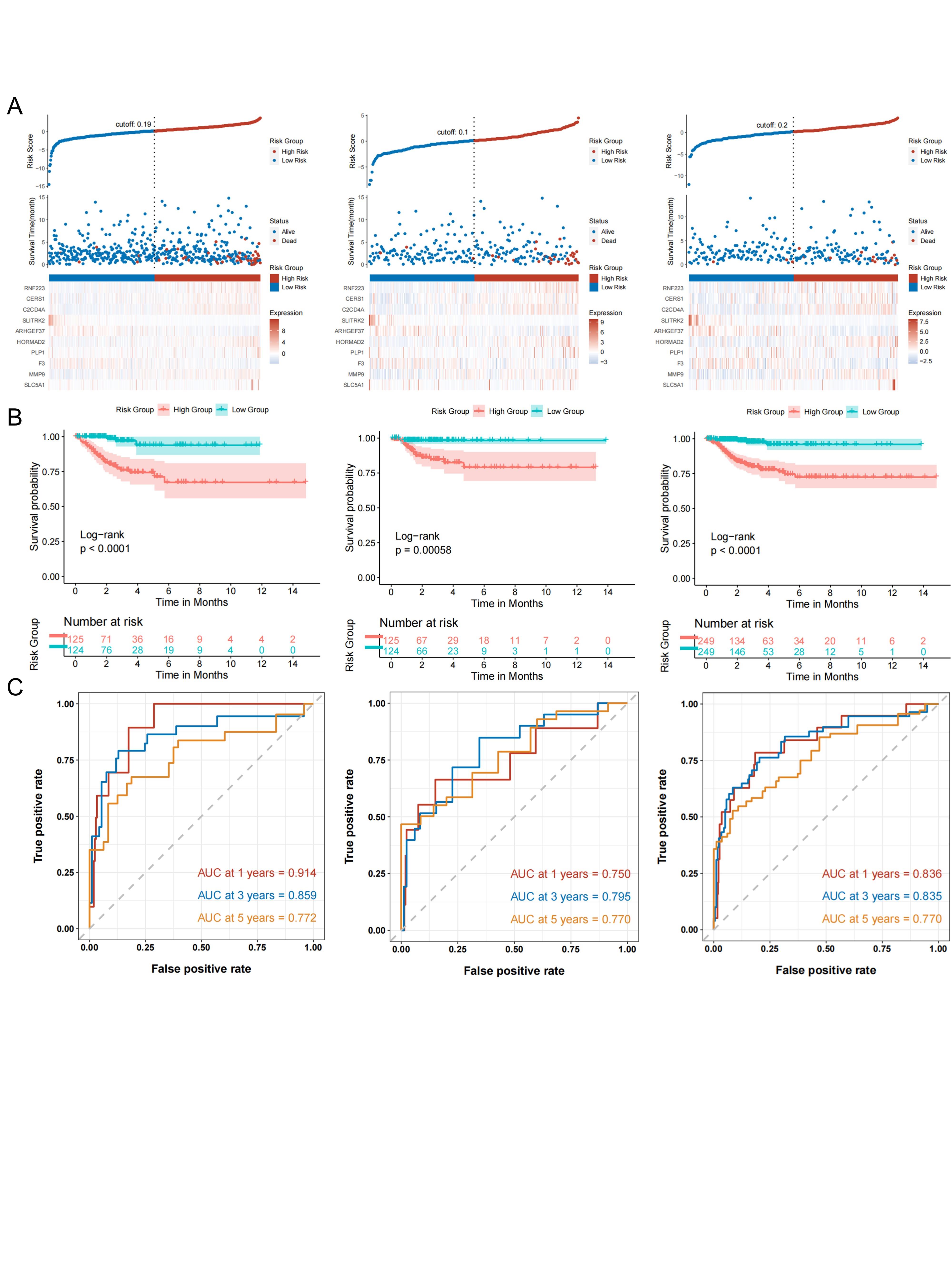


**Supplementary Figure 2. Construction of the consensus clustering.** (A) Risk factor linkage charts of the TCGA training set, test set and total set. (B) KM analyses of DFS of the risk groups in different sets. (C) The ROC analyses for estimating the predictive efficiency.


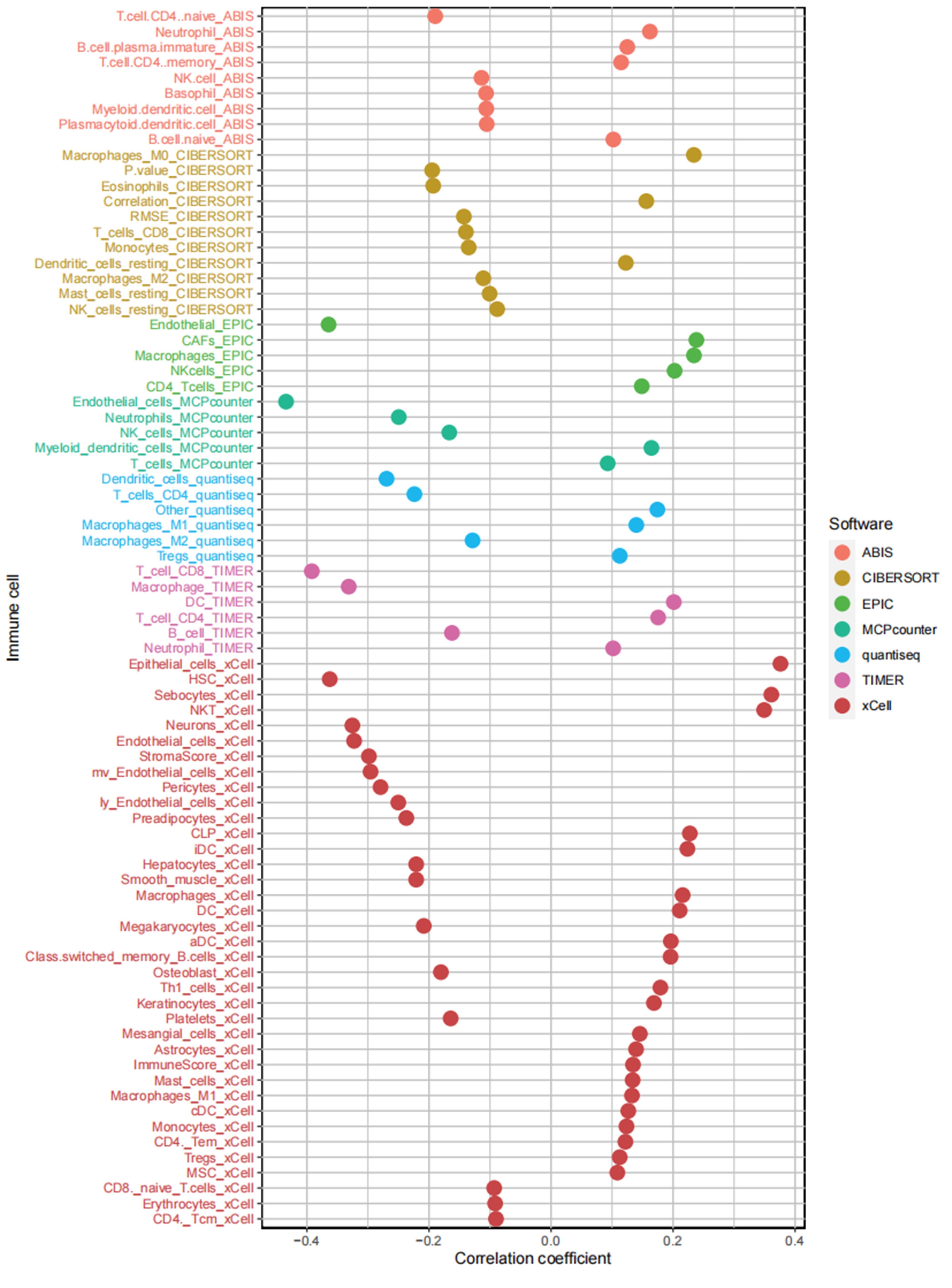


**Supplementary Figure 3. Immune cell correlation analysis based on 7 different algorithms.**

**
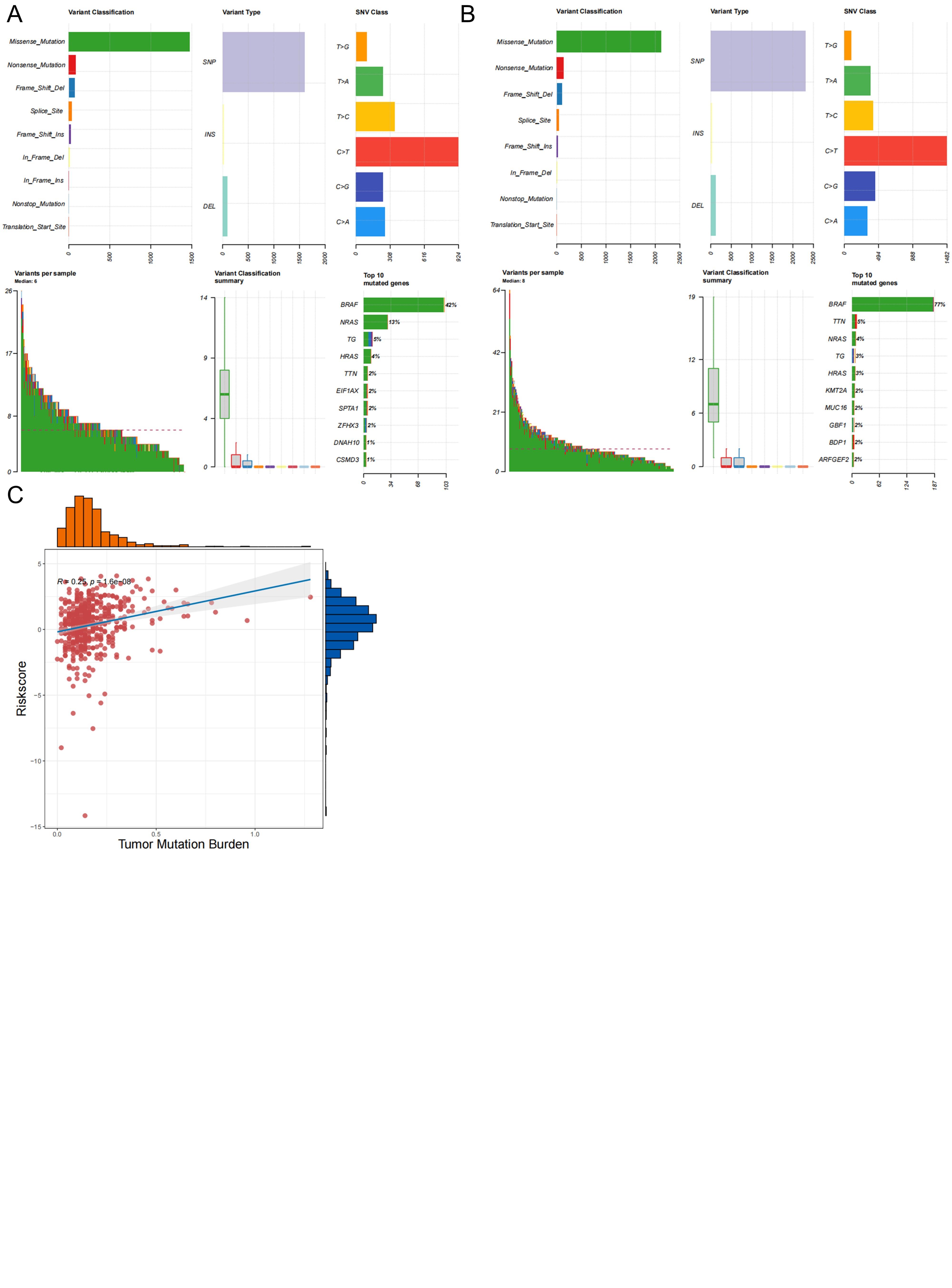
**

**Supplementary Figure 4. TMB differential profiling.** Variant classification, variant type and SNV classification of low-risk group(A) and high-risk group (B). (C) Correlation of risk scores and TMB.

**References:**

1. Terlizzi M, Colarusso C, Ferraro G, Falanga A, Monti MC, Somma P, De Rosa I, Panico L, Pinto A, Sorrentino R. Sex Differences in Sphingosine-1-Phosphate Levels Are Dependent on Ceramide Synthase 1 and Ceramidase in Lung Physiology and Tumor Conditions. Int J Mol Sci. 2023;24(13). eng. 4.9. doi:10.3390/ijms241310841. Cited in: Pubmed; PMID 37446018.

2. Gross KS, Lincoln CM, Anderson MM, Geiger GE, Frick KM. Extracellular matrix metalloproteinase-9 (MMP-9) is required in female mice for 17β-estradiol enhancement of hippocampal memory consolidation. Psychoneuroendocrinology. 2022;141:105773. eng. 3.6. doi:10.1016/j.psyneuen.2022.105773. Cited in: Pubmed; PMID 35490640.

3. Zhang L, Xiong W, Xiong Y, Liu H, Li N, Du Y, Liu Y. Intracellular Wnt/Beta-Catenin Signaling Underlying 17beta-Estradiol-Induced Matrix Metalloproteinase 9 Expression in Human Endometriosis. Biol Reprod. 2016;94(3):70. eng. 3.0. doi:10.1095/biolreprod.115.135574. Cited in: Pubmed; PMID 26888969.

4. Larsson P, Syed Khaja AS, Semenas J, Wang T, Sarwar M, Dizeyi N, Simoulis A, Hedblom A, Wai SN, Ødum N, Persson JL. The functional interlink between AR and MMP9/VEGF signaling axis is mediated through PIP5K1α/pAKT in prostate cancer. Int J Cancer. 2020;146(6):1686-1699. eng. 4.7. doi:10.1002/ijc.32607. Cited in: Pubmed; PMID 31381135.

5. Yamabe N, Kang KS, Lee W, Kim S-N, Zhu BT. Estriol blunts postprandial blood glucose rise in male rats through regulating intestinal glucose transporters. Am J Physiol Endocrinol Metab. 2015;308(5):E370-E379. eng. 3.1. doi:10.1152/ajpendo.00209.2013. Cited in: Pubmed; PMID 25516546.

6. Zhang Y, Tang M, Deng Q, Zhang Y, Zhao J, Zhu Y, Meng Y, Wang S, Liu Z, Guan Y, Li J, Du L. Estrogen-Driven Maintenance of GLUT1/GLUT4/SGLT1 under glucose starvation drives energy homeostasis in bovine PMNs. J Steroid Biochem Mol Biol. 2025;250:106716. eng. 2.5. doi:10.1016/j.jsbmb.2025.106716. Cited in: Pubmed; PMID 40043818.

7. Liu M, Chen J, Hu L, Shi X, Zhou Z, Hu Z, Sha J. HORMAD2/CT46.2, a novel cancer/testis gene, is ectopically expressed in lung cancer tissues. Mol Hum Reprod. 2012;18(12):599-604. eng. 3.5. doi:10.1093/molehr/gas033. Cited in: Pubmed; PMID 22893617.

8. Cai L, Liao Z, Li S, Wu R, Li J, Ren F, Zhang H. PLP1 may serve as a potential diagnostic biomarker of uterine fibroids. Front Genet. 2022;13:1045395. eng. 2.8. doi:10.3389/fgene.2022.1045395. Cited in: Pubmed; PMID 36386836.

9. Prukop T, Epplen DB, Nientiedt T, Wichert SP, Fledrich R, Stassart RM, Rossner MJ, Edgar JM, Werner HB, Nave K-A, Sereda MW. Progesterone antagonist therapy in a Pelizaeus-Merzbacher mouse model. Am J Hum Genet. 2014;94(4):533-546. eng. 8.1. doi:10.1016/j.ajhg.2014.03.001. Cited in: Pubmed; PMID 24680886.

10. Yuan L, Li P, Li J, Peng J, Zhouwen J, Ma S, Jia G, Jia W, Kang P. Identification and gene expression profiling of human gonadotrophic pituitary adenoma stem cells. Acta Neuropathol Commun. 2023;11(1):24. eng. 5.7. doi:10.1186/s40478-023-01517-w. Cited in: Pubmed; PMID 36750863.
